# Confirmatory factor analysis comparing incentivized experiments with self-report methods to elicit adolescent smoking and vaping social norms

**DOI:** 10.1038/s41598-020-72784-z

**Published:** 2020-09-25

**Authors:** Jennifer M. Murray, Erik O. Kimbrough, Erin L. Krupka, Abhijit Ramalingam, Rajnish Kumar, Joanna McHugh Power, Sharon Sanchez-Franco, Olga L. Sarmiento, Frank Kee, Ruth F. Hunter

**Affiliations:** 1 Centre for Public Health, Queen’s University Belfast, Institute of Clinical Sciences, Block B, Royal Victoria Hospital, Grosvenor Road, Belfast, BT12 6BA UK; 2 Smith Institute for Political Economy and Philosophy, Chapman University, One University Drive, Orange, CA 92866 USA; 3 School of Information, University of Michigan, 4322 North Quad, 105 S. State St., Ann Arbor, MI 48109-1285 USA; 4 Department of Economics, Appalachian State University, 416 Howard Street, ASU Box 32051, Boone, NC 28608 USA; 5 Queen’s Management School, Queen’s University Belfast, Riddel Hall, 185 Stranmillis Road, Belfast, BT9 5EE UK; 6 Department of Psychology, Maynooth University, Maynooth, Co. Kildare Ireland; 7 School of Medicine, University of the Andes, Carrera 1 No 18 A – 10, Bloque Q Piso 8, 57 Bogotá, Colombia; 8 Centre for Public Health, Queen’s University Belfast, Institute of Clinical Sciences, Block A, Royal Victoria Hospital, Grosvenor Road, Belfast, BT12 6BA UK

**Keywords:** Human behaviour, Psychology, Health care, Medical research

## Abstract

Many adolescent smoking prevention programmes target social norms, typically evaluated with self-report, susceptible to social desirability bias. An alternative approach with little application in public health are experimental norms elicitation methods. Using the Mechanisms of Networks and Norms Influence on Smoking in Schools (MECHANISMS) study baseline data, from 12–13 year old school pupils (n = 1656) in Northern Ireland and Bogotá (Colombia), we compare two methods of measuring *injunctive* and *descriptive* smoking and vaping norms: (1) incentivized experiments, using monetary payments to elicit norms; (2) self-report scales. Confirmatory factor analysis (CFA) examined whether the methods measured the same construct. Paths from exposures (country, sex, personality) to social norms, and associations of norms with (self-reported and objectively measured) smoking behavior/intentions were inspected in another structural model. Second-order CFA showed that latent variables representing experimental and survey norms measurements were measuring the same underlying construct of anti-smoking/vaping norms (Comparative Fit Index = 0.958, Tucker Lewis Index = 0.951, Root Mean Square Error of Approximation = 0.030, Standardized Root Mean Square Residual = 0.034). Adding covariates into a structural model showed significant paths from country to norms (second-order anti-smoking/vaping norms latent variable: standardized factor loading [β] = 0.30, standard error [SE] = 0.09, *p* < 0.001), and associations of norms with self-reported anti-smoking behavior (β = 0.40, SE = 0.04, *p* < 0.001), self-reported anti-smoking intentions (β = 0.42, SE = 0.06, *p* < 0.001), and objectively measured smoking behavior (β = − 0.20, SE = 0.06, *p* = 0.001). This paper offers evidence for the construct validity of behavioral economic methods of eliciting adolescent smoking and vaping norms. These methods seem to index the same underlying phenomena as commonly-used self-report scales.

## Introduction

Globally, tobacco smoking is still the most important preventable risk factor for chronic disease^1^. Smokers usually start during adolescence when the influence of social norms on behavior is most apparent^2^. Early prevention is critical because young smokers can develop serious chronic health problems and are more sensitive to nicotine addiction^3^. With the introduction of e-cigarettes into the market in the mid-2000s, and as a result of large-scale marketing, e-cigarettes have gained popularity in all age groups, and particularly amongst adolescents^4–7^. Whilst e-cigarettes are seen as a potential cessation aid amongst adults, for adolescents they are more typically used for experimentation, similar to conventional cigarettes, are associated with willingness to smoke, and may act as a “gateway” to smoking^4,8,9^. Therefore, the current study examines social norms for smoking and vaping together. Adolescence is a time when young people are susceptible to social influence and many take their cues from the norms of friends, family, and most importantly, peers^2^. Therefore, many programmes aimed at smoking prevention are anchored in social norms approaches or overtly use peer influencers, with the majority targeting children at the outset of adolescence (around 12–13 years)^10–12^. The Mechanisms of Networks and Norms Influence on Smoking in Schools (MECHANISMS) study aims to prevent smoking amongst adolescents and to investigate the mechanisms through which social norms for smoking and vaping are established and transmitted through social networks in schools^13^.

Social norms can be defined in terms of individuals’ beliefs regarding the actions and beliefs of others in a reference group, and an important distinction has been made between *injunctive* norms (doing what others think one should do) and *descriptive* norms (doing what others do)^14^. Survey-based measures of injunctive norms rely on participants’ self-reports regarding what others who are important to them (e.g. parents, friends, peers) think they “should (not)” do. Similarly, survey-based measures of descriptive norms ask respondents how frequently others who are important to them smoke. Such methods have the benefit of simplicity and clarity, but concerns about social desirability bias arise^15^ because a respondent may perceive that researchers do not approve of smoking, and may not wish to reveal that a parent smokes or would not disapprove of smoking. When considering the issue of social norms measurement for evaluating public health interventions, practical methods which can mitigate the impact of social desirability bias and contribute to understanding mechanisms, are required^14^.

One potential method for eliciting social norms derives from game theory, a branch of economics that has developed well-defined mathematical models describing cooperation and competition. Using incentivized experimental approaches to elicit social norms has gained some traction in behavioral economics^16^, but there has been little evidence of transfer into public health. In behavioral economics research, these methods have been applied to explain behaviors such as reciprocity, co-operation, pro-sociality, or honouring agreements in the presence of a verbal promise^17,18^. The MECHANISMS study applies incentivized experimental approaches to reduce social desirability bias when measuring social norms for adolescent smoking and vaping by asking respondents to guess how *peers* would answer, and providing them with monetary incentives to ‘match’ their own response to the most common response in their school year group. To measure injunctive norms, respondents are asked to guess how peers would rate the social appropriateness of “a parent smoking in front of young children”, for example. Respondents are told that they will be paid a fixed amount if their response “is the same as the most common response provided in your school year group”. This modal response is elicited as the social norm. Since respondents are asked to think about how others will respond, rather than providing personal opinions, the need for social desirability is mitigated^19^. The introduction of incentives to guess how most others are guessing, provides further reason to report beliefs truthfully.

Our experiments’ norm elicitation protocol (NEP) provides several additional advantages over a self-report survey. The underlying theoretical model hypothesizes that behavioral heterogeneity *within* a given setting is related to the degree to which individuals suffer disutility from norm violations or gain from norm adherence (i.e. individuals’ norm-following sensitivities), whilst behavioral heterogeneity *between* different settings is related to the fact that norms vary between settings^17^. Our NEP measures both normative beliefs and norm-following sensitivities to account for these effects. We also observe how strong the ‘norms’ are (whether a relatively large or small proportion of respondents provide the modal response), and whether there are multiple actions of comparable social appropriateness. While experimental methods of norms’ elicitation confer all of these advantages, self-report methods have the advantages of simplicity, low cost, and ease of distribution. Furthermore, the two methods focus on slightly different aspects of norms (the experiments inquire about the beliefs of the reference group whilst the self-report methods ask about influences amongst the respondent’s family, friends and peers, who may or may not be representative of a particular reference group). Thus, we propose that the two methods should be viewed as complementary. Identification of latent norms constructs, and an understanding of their relative ability to explain variance in intervention effects, will improve our ability to understand the active mechanisms in such interventions.

Most studies of norms based public health programmes have been conducted in high-income countries while studies in low-middle income countries (LMICs) are limited^10^. Meanwhile, the tobacco industry has started to strategically target LMICs as its markets are depleted elsewhere^20^. Our study includes data collected from pupils attending schools throughout Northern Ireland [NI] (a constituent country of the United Kingdom [UK], a high income country^21^, with approximately 2 million inhabitants^22^) and Bogotá (the capital city of Colombia, an upper middle income country^23^, with over 7 million inhabitants^24^), and aims to compare results between the two settings where the smoking rates, culture, and social norms are different. For example, current cigarette consumption amongst adolescents aged 11–16 years in NI, is 4% compared to 13.1% in Bogotá for adolescents aged 12–18 years^25,26^. Across the UK, current e-cigarette consumption was 4.9% in 2019 for adolescents aged 11–18 years, similar to rates for conventional cigarettes (5% of adolescents aged 11–15 years)^27,28^. In Colombia, it is estimated that by 2017 e-cigarette consumption among adolescents will have reached the same prevalence rates as cigarette consumption (9% of adolescents aged 13–15 years)^29^. In July 2009, Colombia adopted the World Health Organization’s Framework Convention on Tobacco Control^30^ into legislation, regulating advertising, packaging, sale to the underage population, and smoke-free public places. This was despite tobacco company opposition, reduced state capacity, historical political conditions (e.g. powerful alliances between the tobacco industry and government agricultural agencies, prevalence of tobacco plantations), and efforts to position tobacco as a post-conflict development strategy^31,32^. Thus, Latin American countries have historically been vulnerable to the effects of the tobacco epidemic, and smoking has been integrated into their culture and customs^32^. In the UK, the first tobacco harm reduction programme was introduced in 1972^33^, and whilst there has been a long history of anti-tobacco campaigning^34^, reliance on tobacco industry advice and research previously led to significant delays in introducing more comprehensive tobacco control policies before 1991^33^. Studying the measurement of social norms for adolescent smoking and vaping across such diverse settings will help to better characterize how they spread in schools and impact behavior. Therefore, it is important to understand potential differences in measurement properties of the instruments between the settings.

The current paper aims to compare and contrast the experimental and survey-based social norms measures which were collected as part of the MECHANISMS study.

Specific objectives include to:

1. Investigate the construct and factorial validity of the norms measures;
2. Examine whether the experimental and self-reported norms measures are determined by the same underlying latent construct;
3. Assess cross-country, sex, and personality differences on each latent variable and cross-country differences for individual norms items;
4. Investigate the relationship between the latent norms variables and self-reported anti-smoking behavior, self-reported anti-smoking intentions, and objectively measured smoking behavior.

## Methods

### Study design and participants

Fifteen schools (N = 7 in NI, N = 8 in Bogotá; participation = 90.8%, n = 1656/1824 pupils) took part in the MECHANISMS study between September 2018 and November 2019. We aimed to recruit all pupils in a single year group (aged 11–13 years/Year 9 in NI and 11–15 years/Year 7 in Bogotá, target age 12–13 years). During a single school semester, participants received one of two school-based smoking prevention programmes with proven effectiveness^11,35^. In a pre-post design, pupils participated in incentivized (monetary) norms elicitation experiments, whose design is rooted in the fields of behavioral economics and game theory^16,17,36^, and completed a self-report survey.

Ethics approval was granted from Queen's University Belfast on September 21, 2018 and from Universidad de los Andes, Bogotá Colombia on July 30, 2018. All participants and parents provided informed consent. The experimental protocol, and all data collection procedures, were carried out in accordance with institutional guidelines for research involving human participants. The baseline assessment consisted of two separate sessions with each class in the school year group in each school, during which participants completed an experiment and self-report survey. Experiments and surveys were delivered via the platform Qualtrics (Qualtrics, Provo, Utah, USA) and completed on iPads. Information on study procedures, the study flow diagram, baseline characteristics of participants, and a glossary of terms are available in supplement 1. Prior to implementation in Bogotá, all study instruments underwent a cultural adaptation process including translation into Spanish language and back translation, using the heuristic framework for cultural adaptation proposed by Barrera & Castro^37,38^.

### Incentivized experiments

The incentivized (game theory) experiments consisted of a series of incentivized tasks based on published designs in behavioral economics^16,17,36^. There were four parts to the experiment and the current paper uses data from Parts 1–3. Part 1 consisted of a Rule-Following (RF) task measuring each participant's sensitivity to the effects of social norms^17,36^. The task instructs participants that they have five minutes to allocate 50 balls across two buckets (one blue and one yellow) following an explicitly stated arbitrary rule ("The rule is to put the balls in the blue bucket"). Following the rule imposes explicit monetary costs directly proportional to the degree of rule-following. The central premise is that the more a participant cares intrinsically about rule-following the more willing he/she will be to incur the costs of doing so^36^. Individuals’ norms sensitivities were elicited as the number of balls allocated to the blue (rule-following) bucket.

Parts 2 and 3 of the experiment consisted of a series of incentivized coordination games which used methods employed by Krupka and Weber^16^ to elicit injunctive and descriptive social norms around smoking and vaping in the whole school year group. Participants were provided with financial incentives to *match* their ratings/estimates to other participants' in their school year group as opposed to providing personal opinions. Specifically, participants were informed that they would receive a payment if their response to a randomly selected question matched the most common answer provided in their school year group. Injunctive norms reflect shared beliefs among members of a population about what actions people *ought to* take^16^. Injunctive norms were assessed by asking participants to ‘coordinate’ with others in their school year group to rate the social appropriateness of a series of smoking- and vaping-related situations. Descriptive norms reflect shared beliefs among members of a population about what actions people *actually do* take^16^. Descriptive norms were assessed by asking participants to ‘coordinate’ with others in their school year group to estimate the proportion of their school year group who would be accepting of a close friend smoking or vaping. For each item, the ‘norm’ is elicited as the modal response in the year group. Table 1 shows the assessed smoking- and vaping-related scenarios and numerical coding of responses. More information on the theoretical underpinning of these methods, and full experimental protocols are provided in supplements 1 and 2.

**Table 1:** Smoking/vaping-related injunctive and descriptive social norms elicited in the experiment and self-report survey.

Variable name	Scenario/Question	Responses/Coding
**Experiment Part 1: Rule-following**		
Rule-following (BlueBucket)	Rule-following (individuals’ norms sensitivities): Number of balls allocated to the blue (rule-following) bucket	1 (least rule-following) to 50 (most rule-following)
**Experiment Part 2: Injunctive norms (α = 0.77)**^**a**^		
Part 2 Situation 2 (P2S2)	Parent smoking in their own home in front of children under age of 5	− 1 = Extremely socially inappropriate; − 0.6 = Very socially inappropriate; − 0.2 = Somewhat socially inappropriate; + 0.2 = Somewhat socially appropriate; + 0.6 = Very socially appropriate; + 1 = Extremely socially appropriate
Part 2 Situation 3 (P2S3)	An adult smoking in a car with children under the age of 16 in the car	*As per P2S2*
Part 2 Situation 4 (P2S4)	Someone selling cigarettes to a teenager who looks younger than 16 without requesting proof of age	*As per P2S2*
Part 2 Situation 5 (P2S5)	In a recent superhero movie the lead actor is seen smoking in the opening scene	*As per P2S2*
Part 2 Situation 6 (P2S6)	An older student from school is smoking outside school, for example, at a bus stop	*As per P2S2*
Part 2 Situation 7 (P2S7)	A pupil from school is using an e-cigarette while walking to school	*As per P2S2*
Part 2 Situation 8 (P2S8)	A pupil from school shares a photograph of him/herself using an e-cigarette on social media	*As per P2S2*
Part 2 Situation 9 (P2S9)	A pupil from school is chewing tobacco	*As per P2S2*
**Experiment Part 3: Descriptive norms (α = 0.85)**^**a**^		
Part 3 Question 1 (P3Q1)	The proportion of my peers who would be accepting of a close friend smoking	− 1 = None of my peers; − 0.6 = Only a few of my peers; − 0.2 = Some of my peers; + 0.2 = A lot of my peers; + 0.6 = Most of my peers; + 1 = All of my peers
Part 3 Question 2 (P3Q2)	The proportion of my peers who would be accepting of a close friend vaping	*As per P3Q1*
**Survey: Self-reported injunctive norms (α = 0.74)**^**b,c**^		
Injunctive Norms 1 (IN1)	Most of the people who are important to me think that I…	− 2 = Definitely should smoke; − 1 = Maybe should smoke; 0 = Don't know/neutral; + 1 = Maybe should not smoke; + 2 = Definitely should not smoke
Injunctive Norms 2 (IN2)	My mother thinks that I…	*As per IN1. Responses of “I don’t have a mother” were also set to 0*
Injunctive Norms 3 (IN3)	My father thinks that I…	*As per IN1. Responses of “I don’t have a father” were also set to 0*
Injunctive Norms 4 (IN4)	My brother(s) think(s) that I…	*As per IN1. Responses of “I don’t have a brother” were also set to 0*
Injunctive Norms 5 (IN5)	My sister(s) think(s) that I…	*As per IN1. Responses of “I don’t have a sister” were also set to 0*
Injunctive Norms 6 (IN6)	My friends think that I…	*As per IN1. Responses of “I don’t have a friend” were also set to 0*
Injunctive Norms 7 (IN7)	My best friend thinks that I…	*As per IN1. Responses of “I don’t have a best friend” were also set to 0*
**Survey: Self-reported descriptive norms (α = 0.54)**^**b,d**^		
Descriptive Norms 1 (DN1)	Does your best friend smoke?	1 = Very often; 2 = Often; 3 = Occasionally; 4 = Rarely; 5 = Never/Don't know. *Responses of “I don’t have a best friend” were also set to 5*
Descriptive Norms 2 (DN2)	Does your mother smoke?	*As per DN1. Responses of “I don’t have a mother” were also set to 5*
Descriptive Norms 3 (DN3)	Does your father smoke?	*As per DN1. Responses of “I don’t have a father” were also set to 5*
Descriptive Norms 4 (DN4)	Do any of your brothers smoke?	*As per DN1. Responses of “I don’t have a brother” were also set to 5*
Descriptive Norms 5 (DN5)	Do any of your sisters smoke?	*As per DN1. Responses of “I don’t have a sister” were also set to 5*
**Survey: Self-reported smoking behavior and intentions**^**b**^		
Past Smoking Behavior (SmokePast)	Now read the following statements carefully and tick the box next to the one which best describes you. (I have never smoked; I have only ever tried smoking once; I used to smoke sometimes but I never smoke a cigarette now; I sometimes smoke cigarettes now but I don’t smoke as many as one a week)	1 = Sometimes smoke; 2 = Previous smoker; 3 = Smoked once; 4 = Never smoked
Intentions (Intent)	If you DON’T currently smoke, do you intend to take up smoking in the next 6 months?	1 = I am a smoker; 2 = Definitely start smoking; 3 = Probably start smoking; 4 = Don't know; 5 = Probably remain; 6 = Definitely remain a non-smoker
**Smokerlyzer readings: Objectively measured smoking behavior**		
Carbon monoxide reading (COreading)	Objectively measured smoking behavior over the past 24 h captured using hand-held carbon monoxide monitors (PICOAdvantage Smokerlyzer, Bedfont) to measure expelled air carbon monoxide in parts per million (ppm) in a range of 0–150 ppm with an accuracy of 2 ppm/5% (whichever is greater)	Continuous variable (ppm)
**Survey: Self-reported sex and personality characteristics**^**b**^		
Sex	Participant sex	0 = Boy; 1 = Girl/Prefer not to say
Need to Belong (Belong)	Need to Belong Scale	Average of 10 items, each coded 1–5: 1 (lowest need to belong)-5 (greatest need to belong). Not available for two Colombian schools
Fear of Negative Evaluation (Negative)	Fear of Negative Evaluation Scale	Average of 12 items, each coded 1–5: 1 (lowest fear of negative evaluation)-5 (greatest fear of negative evaluation). Not available for two Colombian schools
Prosocial Behavior (Prosocial)	Prosocial Behavior Scale	Sum of five items, each coded 0–2: 0 (least prosocial)-10 (most prosocial)
Big 5 Openness (Big5Open)	Big 5 Personality Questionnaire (Openness subscale)	Average of 10 items, each coded 0–4: 0 (lowest openness)-4 (greatest openness)
Big 5 Extraversion (Big5Extra)	Big 5 Personality Questionnaire (Extraversion subscale)	Average of 10 items, each coded 0–4: 0 (least extraverted)-4 (most extraverted)
Big 5 Agreeableness (Big5Agree)	Big 5 Personality Questionnaire (Agreeableness subscale)	Average of 10 items, each coded 0–4: 0 (least agreeable)-4 (most agreeable)
Big 5 Conscientiousness (Big5Cons)	Big 5 Personality Questionnaire (Conscientiousness subscale)	Average of 10 items, each coded 0–4: 0 (least conscientious)-4 (most conscientious)
Big 5 Stability (Big5Stab)	Big 5 Personality Questionnaire (Stability subscale)	Average of 10 items, each coded 0–4: 0 (least stability)-4 (most stability)

^a^Responses to experimental items were numerically coded to run between − 1 and + 1 following procedures adopted in Krupka and Weber^16^.

^b^All items on the survey were coded such that higher numerical values represented greater anti-smoking norms, greater anti-smoking behavior or intentions, or higher values of the personality traits (Need to Belong, Fear of Negative Evaluation, Pro-social Behavior, Big 5 Personality Questionnaire).

^c^Responses to survey injunctive norms items were numerically coded to run between − 2 and + 2 following Cremers et al.^92^.

^d^Responses to survey descriptive norms items were numerically coded to run between + 1 and + 5 following Cremers et al.^92^.

### Self-report survey

A survey was used to collect socio-demographic and personal characteristics, social networks data, past and present smoking behavior and intentions, psychosocial constructs and wellbeing. All survey items were previously validated and adopted from studies conducted with children of a similar age^13^. The current paper uses data collected from seven items measuring injunctive social norms for smoking^39^, five items measuring descriptive social norms for smoking^39^, one item measuring past smoking behavior^40,41^, one item measuring smoking intentions over the next six months^42^, a ten-item Need to Belong scale^43,44^, a 12-item Fear of Negative Evaluation Scale^44–46^, a five-item Pro-social Behavior Scale^44,47^, and the five subscales of the “Big 5” Personality Questionnaire^48,49^ (Table 1). Pupils also had their smoking behavior in the last 24 h measured using a hand-held carbon monoxide monitor (PICOAdvantage Smokerlyzer, Bedfont)^50^. This is an electrochemical sensor which measures expelled air carbon monoxide in parts per million (ppm) in a range of 0–150 ppm with an accuracy of 2 ppm/5% (whichever is greater)^50^. A pupil was considered to have engaged in smoking behavior if they provided a reading of > 9 ppm in line with previous research^35,51^. We analysed objective smoking behavior as a continuous variable (expelled air carbon monoxide in ppm)^35^.

### Statistical analysis

The statistical analysis was guided by the following specific objectives:

1. To conduct confirmatory factor analysis (CFA) to explore the construct and factorial validity of the norms measures;
2. To investigate whether the experimental and self-reported norms measures are determined by the same underlying latent construct using second-order CFA;
3. To assess cross-country, sex, and personality differences on each latent variable using multiple indicators multiple causes (MIMIC) modelling^52,53^, and cross-country differences for individual items using differential item functioning (DIF) analysis;
4. To investigate the relationship between the DIF-adjusted latent “anti-smoking/vaping norms” variables and self-reported anti-smoking behavior, self-reported anti-smoking intentions, and objectively measured smoking behavior, using structural equation modelling (SEM).

Analyses were conducted using Stata 13 (StataCorp)^54^ and R version 3.6.1^55^. Means and standard deviations were computed and histograms were graphed to visualize distributions of all variables. Cronbach's alpha coefficients were computed for: (1) experimentally derived injunctive norms; (2) experimentally derived descriptive norms; (3) survey injunctive norms; (4) survey descriptive norms. As a preliminary step, we examined whether individual norms items from the experiments (Part 2 Situations 2–9, Part 3 Questions 1–2) and survey (Injunctive Norms 1–7, Descriptive Norms 1–5) were showing theoretically expected inter-relationships and associations with self-reported anti-smoking behavior, intentions, and objectively measured smoking behavior. Spearman's rank-order correlations were computed, examining the association between individual norms items from the experiments and survey, and associations between self-reported anti-smoking behavior, intentions, and objectively measured smoking behavior. Individual norms items were examined for an association with self-reported anti-smoking behavior, self-reported anti-smoking intentions, and objectively measured smoking behavior, using mixed-effects regressions. Rule-following was compared between NI and Colombia using a cluster-adjusted t-test with number of balls allocated to the blue bucket in the RF task as the outcome and participant school as the cluster variable. This was carried out using Stata’s ‘clttest’ command.

**Figure 1:**
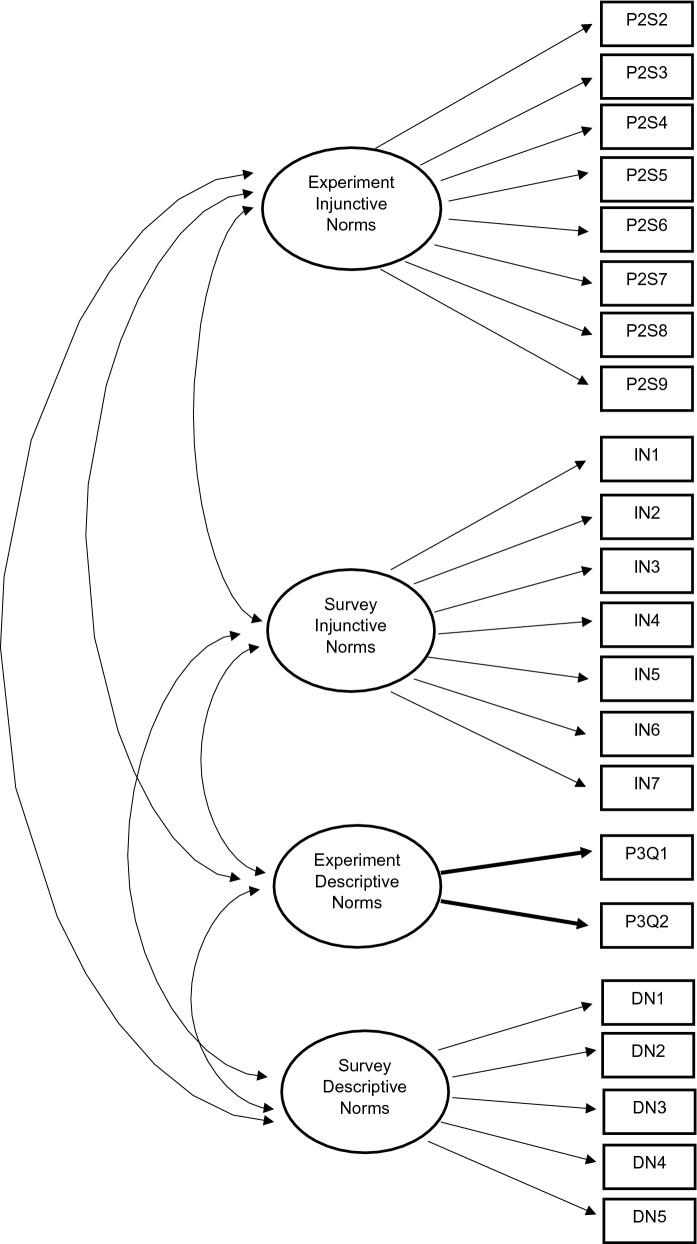
Theoretic first-order measurement model with four correlated latent variables.

**Figure 2:**
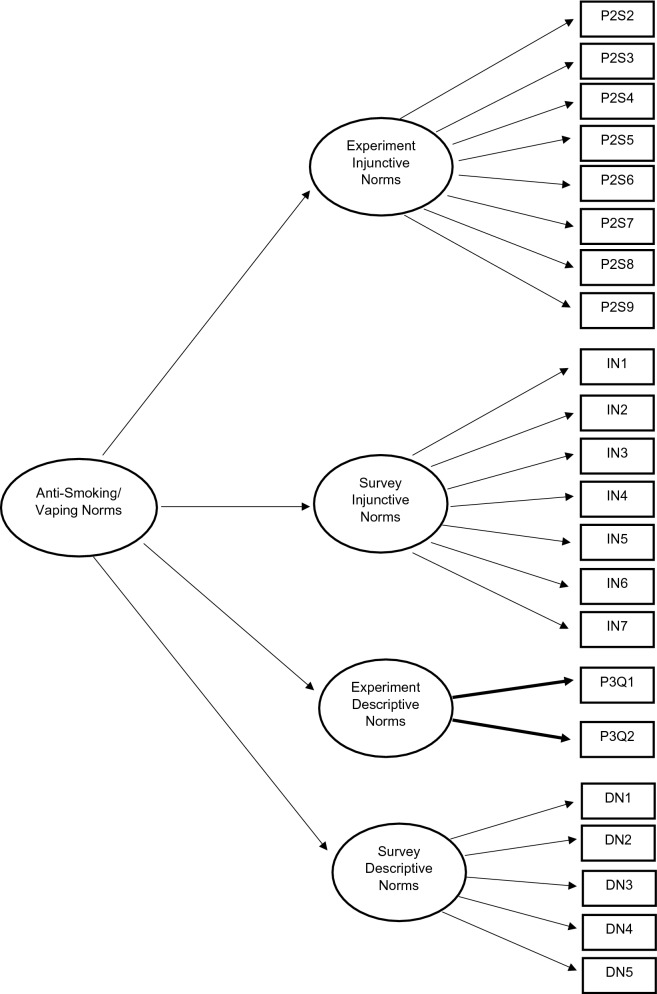
Theoretic second-order measurement model with four first-order latent variables.

CFA is a statistical technique to determine whether measures of a construct are consistent with a researcher’s understanding of the nature of the construct, or factor, by testing whether the data fits a hypothesized measurement model^56^. To assess factorial and construct validity, separate CFAs were conducted for: experimental injunctive norms (model 1); survey injunctive norms (model 2); experimental descriptive norms (model 3); survey descriptive norms (model 4; objective 1). To compare the experimental and survey measurements, a CFA model was conducted containing four correlated first-order latent variables (model 5; Fig. 1). A final CFA model was derived, similar to model 5, in which the covariance between the first-order latent variables was described by an overall second-order latent construct labelled “Anti-Smoking/Vaping Norms” (model 6; Fig. 2; objective 2)^57^. Since our experimentally derived measure of descriptive norms consisted only of two items, convergence was achieved by constraining the loadings of both indicators to be equal^58^.

CFAs were conducted using the lavaan package in R^59^. To reduce bias in standard errors which threatens maximum likelihood estimation^60–62^, robust standard errors were computed (Huber White)^63,64^. This estimator was favoured over the categorical estimators since all indicators had more than five response categories^62,65,66^. It also permitted imputation of missing data using full information maximum likelihood. The Little (1988) test was used to determine whether data for individual items were missing completely at random (MCAR) using Stata’s ‘mcartest’ command with 200 iterations in the expectation maximization algorithm^67^. A *p* value of < 0.0001 was obtained, indicating that the data were not MCAR, which justified imputing missing data^68^. All analyses were repeated without imputation of missing data (i.e. complete cases). Percentages of missing data requiring imputation for individual items ranged from 3.7–3.9% (experimentally derived injunctive norms), 4.2–4.5% (survey injunctive norms), 3.7–3.9% (experimentally derived descriptive norms), and 4.16–4.22% (survey descriptive norms). For the most part, missing data occurred if a participant was present in school on one of the days (for the experiment or the survey), but absent on the other day (n = 123/1636 = 7.5% of participants).

Model fit was assessed in relation to several goodness-of-fit indices. The chi-square statistic can be used to assess the absolute fit of the model to the data, assuming correct model specification^57,69^. A non-significant result (*p* > 0.05) indicates good model fit. However, it can be overly influenced by sample size, correlations, variance unrelated to the model, and multivariate non-normality^69,70^. Comparative Fit Index (CFI) values of ≥ 0.96, Tucker-Lewis Index (TLI) values of ≥ 0.95, Root Mean Square Error of Approximation (RMSEA) values of ≤ 0.06, and Standardized Root Mean Square Residual (SRMR) values of ≤ 0.09 indicate good model fit^69,71^. A number of parsimony based fit indices were also extracted including the Akaike Information Criterion (AIC), Bayesian Information Criterion (BIC) and adjusted BIC. Lower values on these indices indicate a more parsimonious model^69^. Measurement models were modified to improve factorial validity by reference to modification indices (MIs)^72^. Modifications were made only where substantively appropriate, and with strong theoretical justification^69,73,74^. Subsequent analyses were based on the second-order measurement model. Supplement 3 shows syntax for all analyses.

MIMIC models can be used to examine differences on latent variables by regressing them onto an observed grouping variable. Individual items can then be tested for DIF by regressing them onto the grouping variable whilst controlling for differences at the latent variable level^52,53,75^. These techniques were used to compare the norms measurements, and to assess measurement invariance, between NI and Colombia (objective 3). Baseline MIMIC models included a measurement model and a structural model: (1) the second-order latent variable regressed onto an observed country variable (0 = NI, 1 = Colombia); (2) the four first-order latent variables regressed simultaneously onto the observed country variable. This showed whether mean values on the overall latent constructs differed between the two countries. DIF occurs when an item has different measurement properties for one group versus another, irrespective of mean differences on the overall latent construct^76^. To determine which indicators showed DIF, direct paths between country and each observed indicator were constrained to 0, whilst controlling for country differences on the four first-order latent constructs. MIs were inspected along with expected parameter changes (EPCs) and DIF was determined to be present for an item if MI > 3.84 and EPC ≥ 0.10^77^. This novel approach to assessing DIF has been adopted from a recent study^78^. In the case of low power (< 0.80), if these conditions were not met, the result was determined as inconclusive.

MIMIC models were also used to determine whether mean values on the overall first- and second-order latent constructs (adjusted for country differences on first-order latent variables and DIF) differed according to sex, personality characteristics (Need to Belong, Fear of Negative Evaluation, Pro-social Behavior, Big 5 personality subscales), and rule-following (number of balls allocated to the blue bucket in the RF task). We also examined, and found no evidence for, DIF according to participant sex (results not reported).

The DIF-adjusted second-order measurement model was investigated for associations with observed self-reported anti-smoking behavior, intentions, and objectively measured smoking behavior, using SEM (objective 4). The structural part of these models included either self-reported anti-smoking behavior, self-reported anti-smoking intentions, or objectively measured smoking behavior as the observed outcome variable regressed onto: (1) the second-order latent variable; (2) the four first-order latent variables simultaneously. Path coefficients were inspected (*p* < 0.05 provided evidence for a significant association).

### Ethical statements

This study complies with all relevant ethical regulations.

## Results

Descriptive statistics are shown in Table 2 and supplement 4. Mean responses for all experimental items are < 0, indicating there were already anti-smoking norms established at baseline. Details on the methods and results of the correlational analyses and mixed-effects regressions are discussed in supplement 5. Individual items from the experiments and survey showed theoretically expected inter-relationships and associations with self-reported anti-smoking behavior and intentions (e.g. higher anti-smoking/vaping norm responses were associated with greater anti-smoking behavior and intentions). Theoretically expected inter-relationships were observed between self-reported anti-smoking behavior and intentions, and objectively measured smoking behavior. These models also indicate that (1) pupils who were more rule-following in the RF task were more likely to report higher anti-smoking behavior and intentions; (2) Colombian pupils were more likely to report lower anti-smoking behavior or intentions and to show higher levels of expelled air carbon monoxide in their Smokerlyzer readings (which accords with intercountry differences in smoking prevalence among adolescents). A cluster-adjusted t-test showed there were no between-country differences in rule-following (number of balls allocated to the blue bucket in the RF task, *p* = 0.19).

**Table 2:** Baseline summary statistics, means and standard deviations.

	Northern Ireland (N = 7)	Colombia (N = 8)	All schools (N = 15)
Experiment, n	696	880	1576
Survey, n	701	872	1573
**Experiment Part 1**: Balls allocated to blue (rule-following) bucket	28.8 (19.2)	31.6 (16.9)	30.4 (18.0)
**Experiment Part 2 (injunctive social norms)**^**a**^			
P2S2	− 0.8 (0.3)	− 0.9 (0.2)	− 0.9 (0.3)
P2S3	− 0.7 (0.4)	− 0.7 (0.3)	− 0.7 (0.4)
P2S4	− 0.9 (0.3)	− 0.9 (0.3)	− 0.9 (0.3)
P2S5	− 0.3 (0.4)	− 0.5 (0.4)	− 0.4 (0.4)
P2S6	− 0.6 (0.4)	− 0.5 (0.4)	− 0.6 (0.4)
P2S7	− 0.5 (0.4)	− 0.6 (0.4)	− 0.5 (0.4)
P2S8	− 0.5 (0.4)	− 0.5 (0.4)	− 0.5 (0.4)
P2S9	− 0.8 (0.4)	− 0.8 (0.3)	− 0.8 (0.3)
**Experiment Part 3 (descriptive social norms)**^**b**^			
P3Q1	− 0.5 (0.5)	− 0.5 (0.5)	− 0.5 (0.5)
P3Q2	− 0.3 (0.6)	− 0.5 (0.5)	− 0.4 (0.5)
**Survey**: Smoking behavior^c^	3.8 (0.6)	3.7 (0.7)	3.8 (0.6)
**Survey**: Smoking intentions^d^	5.7 (0.8)	5.5 (1.2)	5.6 (1.1)
**Survey: Injunctive social norms**^**e**^			
IN1	1.7 (0.7)	1.8 (0.6)	1.8 (0.7)
IN2	1.9 (0.3)	1.9 (0.4)	1.9 (0.4)
IN3	1.8 (0.6)	1.7 (0.7)	1.7 (0.7)
IN4	1.4 (0.9)	1.4 (0.9)	1.4 (0.9)
IN5	1.4 (0.9)	1.4 (0.9)	1.4 (0.9)
IN6	1.5 (0.9)	1.3 (1.0)	1.4 (0.9)
IN7	1.7 (0.7)	1.5 (0.9)	1.6 (0.8)
**Survey: Descriptive social norms**^**f**^			
DN1	4.8 (0.8)	4.8 (0.7)	4.8 (0.7)
DN2	4.2 (1.4)	4.6 (1.0)	4.4 (1.2)
DN3	4.2 (1.4)	4.4 (1.2)	4.3 (1.3)
DN4	4.7 (0.9)	4.7 (0.8)	4.7 (0.9)
DN5	4.8 (0.7)	4.8 (0.7)	4.8 (0.7)
**Survey: Sex and psycho-social variables**			
Sex, n(%)^g^			
Boys	335 (47.8%)	436 (50.0%)	771 (49.0%)
Girls	355 (50.6%)	431 (49.4%)	786 (50.0%)
Prefer not to say	11 (1.6%)	5 (0.6%)	16 (1.0%)
Need to Belong Scale (1–5)^h^	3.1 (0.6)	2.8 (0.6)	3.0 (0.6)
Fear of Negative Evaluation (1–5)^i^	2.9 (0.7)	2.6 (0.6)	2.7 (0.7)
Pro-social Behavior (0–10)^j^	8.1 (2.1)	7.3 (2.1)	7.6 (2.1)
Big 5 (Openness; 0–4)^k^	2.4 (0.6)	2.7 (0.7)	2.6 (0.7)
Big 5 (Extraversion; 0–4)^k^	2.6 (0.8)	2.7 (0.7)	2.6 (0.7)
Big 5 (Agreeableness; 0–4)^k^	1.9 (0.8)	2.6 (0.7)	2.6 (0.7)
Big 5 (Conscientiousness; 0–4)^k^	2.7 (0.7)	2.4 (0.6)	2.4 (0.7)
Big 5 (Stability; 0–4)^k^	1.9 (0.8)	2.1 (0.7)	2.0 (0.7)
**Smokerlyzer readings:** Objective smoking behavior (carbon monoxide, ppm)^l^	1.5 (1.4)	3.4 (1.5)	2.5 (1.7)

^a^− 1 = Extremely socially inappropriate; − 0.6 = Very socially inappropriate; − 0.2 = Somewhat socially inappropriate; 0.2 = Somewhat socially appropriate; 0.6 = Very socially appropriate; 1 = Extremely socially appropriate.

^b^− 1 = None of my peers; − 0.6 = Only a few of my peers; − 0.2 = Some of my peers; + 0.2 = A lot of my peers; + 0.6 = Most of my peers; + 1 = All of my peers.

^c^1 = Sometimes smoke; 2 = Previous smoker; 3 = Smoked once; 4 = Never smoked.

^d^1 = I am a smoker; 2 = Definitely start smoking; 3 = Probably start smoking; 4 = Don't know; 5 = Probably remain; 6 = Definitely remain a non-smoker.

^e^− 2 = Definitely should smoke; − 1 = Maybe should smoke; 0 = Don't know/neutral; + 1 = Maybe should not smoke; + 2 = Definitely should not smoke. "I don't have…" responses set to 0.

^f^1 = Very often; 2 = Often; 3 = Occasionally; 4 = Rarely; 5 = Never/Don't know. “I don’t have…” responses set to 5.

^g^In all analyses, sex is coded (0 = Boy; 1 = Girl/Prefer not to say).

^h^Average of 10 items, coded 1–5. Not available for two Colombian schools (excluded from analysis).

^i^Average of 12 items, coded 1–5. Not available for two Colombian schools (excluded from analysis).

^j^Sum of five items, coded 0–2.

^k^Average of 10 items, coded 0–4.

^l^Not available for one Northern Irish school and two Colombian schools (excluded from analysis).

**Figure 3:**
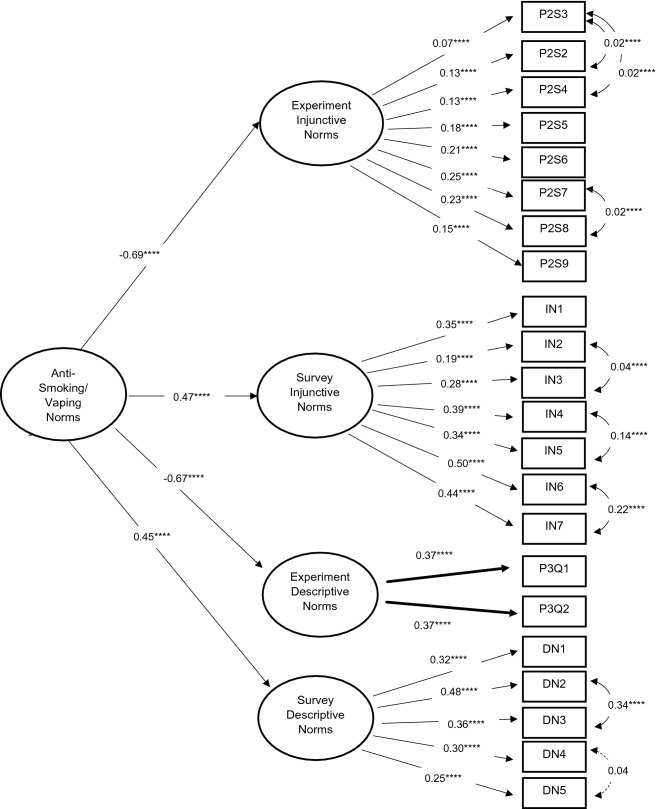
Second-order measurement model with four first-order latent variables, standardized factor loadings, *p < 0.10; **p < 0.05; ***p < 0.01; ****p ≤ 0.001.

Goodness-of-Fit statistics for our CFA models are shown in supplement 6. Although chi-square tests were significant for almost all of the models (*p* < 0.05), we did not reject models on this basis as it can be overly influenced by sample size, correlations, variance unrelated to the model, and multivariate non-normality^69,70^. CFI values ranged from 0.958–1.000 and TLI values ranged from 0.947–1.017. RMSEA values ranged from 0.000–0.059 and SRMR values ranged from 0.000–0.034. Therefore, all models demonstrated a good or satisfactory fit (objective 1). Inspection of model fit indices indicated there was almost an identical fit between the first-order measurement model with four correlated latent variables (model 5; Fig. 1) and the second-order measurement model (model 6; Fig. 2). Subsequent analyses were based on the second-order measurement model. Diagrams showing final model structures and standardized factor loadings are provided in supplement 6 (Fig. 3 shows results for the final second-order measurement model).

The second-order measurement model showed negative standardized factor loadings for the paths connecting the first-order latent constructs representing experimentally derived norms to the underlying second-order latent construct (injunctive norms: standardized factor loading [β] = − 0.69, standard error [SE] = 0.11, *p* < 0.001; descriptive norms: β = − 0.67, SE = 0.10, *p* < 0.001), and positive standardized factor loadings for the paths connecting the first-order latent constructs representing survey norms to the underlying second-order latent construct (injunctive norms: β = 0.47, SE = 0.08, *p* < 0.001; descriptive norms: β = 0.45, SE = 0.10, *p* < 0.001). This is as expected since the experiment and survey items were coded in the opposite directions intuitively. Thus, higher values on the second-order latent variable represent greater anti-smoking/vaping norms. Therefore, we concluded that our hypothesized measurement models showed good fit to the data, and our four first-order latent variables were measuring the same overall second-order latent variable of “Anti-Smoking/Vaping Norms” (objective 2).

Baseline MIMIC models indicated that there was an association between country and the second-order latent variable measuring anti-smoking/vaping norms (β = 0.30, SE = 0.09, *p* < 0.001). Therefore, Colombian pupils reported greater anti-smoking norms overall. There were significant intercountry differences for the first-order latent variables measuring experimentally derived injunctive norms (β = − 0.21, SE = 0.08, *p* = 0.007), survey injunctive norms (β = − 0.21, SE = 0.07, *p* = 0.004), experimentally derived descriptive norms (β = − 0.31, SE = 0.07, *p* < 0.001), and survey descriptive norms (β = 0.30, SE = 0.12, *p* = 0.008) (Table 3). Therefore, Colombian pupils were more likely to give lower social appropriateness ratings in their experiment injunctive norms responses, and to rate that a lower proportion of their school year group would be accepting of a close friend smoking/vaping in their experiment descriptive norms responses. Colombian pupils were also more likely to think that people who are important to them (e.g. parents, siblings) would be more accepting of their own smoking behavior in their survey injunctive norms responses, and more likely to think that people who are important to them smoke less frequently in their survey descriptive norms responses.

**Table 3:** Effects of country on first-order norms latent variables, second-order norms latent variables and observed indicators, standardized regression coefficients.

Latent variable/Observed indicator	Baseline MIMIC model		DIF corrected model	
	Β (SE)	*p* value	Β (SE)	*p* value
**Second-order latent variables**				
Anti-Smoking/Vaping Norms	0.30 (0.09)	< 0.001	–	–
**First-order latent variables**				
Expt. Inj. Norms	− 0.21 (0.08)	0.007	− 0.11 (0.07)	0.15
Sur. Inj. Norms	− 0.21 (0.07)	0.004	− 0.35 (0.08)	< 0.001
Expt. Desc. Norms	− 0.31 (0.07)	< 0.001	− 0.31 (0.07)	< 0.001
Sur. Desc. Norms	0.30 (0.12)	0.008	0.03 (0.09)	0.75
**Indicators**^**a**^				
P2S2	–	–	− 0.10 (0.01)	< 0.001
P2S5	–	–	− 0.14 (0.02)	< 0.001
IN1	–	–	0.15 (0.03)	< 0.001
IN4	–	–	0.15 (0.04)	0.001
DN2	–	–	0.38 (0.06)	< 0.001
DN3	–	–	0.22 (0.07)	0.001

*MIMIC* multiple indicators multiple causes, *DIF* differential item functioning.

^a^Controlling for country differences on the underlying first-order latent variable (0 = Northern Ireland, 1 = Colombia).

Controlling for differences on the latent variables, there was evidence that the following items may be exhibiting DIF: Part 2 Situation 2, Part 2 Situation 5, Part 2 Situation 8, Injunctive Norms 1, Injunctive Norms 3, Injunctive Norms 4, Injunctive Norms 7, Descriptive Norms 2, Descriptive Norms 4, and Descriptive Norms 5. Results were inconclusive for Injunctive Norms 5, Injunctive Norms 6, Descriptive Norms 1, and Descriptive Norms 3 due to low power (supplement 7). There was no further evidence of DIF with the paths from country to the following indicators freely estimated: Part 2 Situation 2, Part 2 Situation 5, Injunctive Norms 1, Injunctive Norms 4, Descriptive Norms 2, and Descriptive Norms 3 (Table 3). After adjusting for DIF, the path from country to the first-order latent variable measuring experimental injunctive norms was no longer statistically significant (*p* = 0.15) suggesting that between-country differences on this latent variable were due to the items Part 2 Situation 2 and Part 2 Situation 5. After adjusting for DIF, the path from country to the first-order latent variable measuring survey descriptive norms was no longer statistically significant (*p* = 0.75) suggesting that between-country differences on this latent variable were due to the items Descriptive Norms 2 and Descriptive Norms 3.

Results of MIMIC models examining associations between sex, personality characteristics, and rule-following with latent norms variables are reported in supplement 8. For the second-order latent construct there were significant positive associations with the following variables: Need to Belong (*p* = 0.003), Pro-Social Behavior (*p* < 0.001), Openness (*p* < 0.001), Extraversion (*p* = 0.03), Agreeableness (*p* < 0.001), Conscientiousness (*p* < 0.001), and Stability (*p* < 0.001). Thus, higher levels on these personality variables were associated with higher anti-smoking/vaping norms. Results are also presented for associations with first-order latent norms constructs (objective 3).

The results of SEM models are shown in Table 4. Higher anti-smoking/vaping norms (on the second-order latent variable) were associated with higher self-reported anti-smoking behavior (β = 0.40, SE = 0.04, *p* < 0.001), higher self-reported anti-smoking intentions (β = 0.42, SE = 0.06, *p* < 0.001), and lower objectively measured smoking behavior (β = − 0.20, SE = 0.06, *p* = 0.001). These models also show a negative association between country and self-reported anti-smoking behavior and intentions suggesting that Colombian pupils were more likely to report higher levels of past/current smoking behavior or greater intentions to take up smoking in the next six months compared to NI pupils. There was also a positive association between country and objective smoking behavior suggesting that Colombian pupils showed higher levels of expelled air carbon monoxide in their Smokerlyzer readings (objective 4).

**Table 4:** DIF-adjusted models predicting self-reported anti-smoking behavior, self-reported anti-smoking intentions, and objectively measured smoking behavior.

Parameter	Outcome variable					
	Anti-smoking behavior		Anti-smoking intentions		Objective smoking behavior^a^	
	Β (SE)	*p* value	Β (SE)	*p* value	Β (SE)	*p* value
**Second-order latent norm variables as predictor**						
Outcome variable						
Anti-Smoking/Vaping Norms (second-order latent)	0.40 (0.04)	< 0.001	0.42 (0.06)	< 0.001	− 0.20 (0.06)	0.001
Country (observed)	− 0.11 (0.03)	0.001	− 0.26 (0.05)	< 0.001	1.83 (0.08)	< 0.001
**First-order latent norms variables as predictors**						
Outcome variable						
Expt. Inj. Norms (first-order latent)	0.004 (0.02)	0.83	− 0.007 (0.03)	0.81	0.02 (0.05)	0.67
Sur. Inj. Norms (first-order latent)	0.07 (0.03)	0.01	0.17 (0.04)	< 0.001	− 0.02 (0.05)	0.63
Expt. Desc. Norms (first-order latent)	− 0.01 (0.02)	0.47	− 0.002 (0.03)	0.94	0.10 (0.04)	0.02
Sur. Desc. Norms (first-order latent)	0.31 (0.04)	< 0.001	0.28 (0.05)	< 0.001	− 0.05 (0.06)	0.44
Country (observed)	− 0.11 (0.03)	0.001	− 0.21 (0.05)	< 0.001	1.85 (0.08)	< 0.001
Expt. Inj. Norms (first-order latent)						
Country (observed)	− 0.11 (0.07)	0.14	− 0.11 (0.07)	0.15	0.03 (0.08)	0.75
Sur. Inj. Norms (first-order latent)						
Country (observed)	− 0.35 (0.08)	< 0.001	− 0.35 (0.08)	< 0.001	− 0.37 (0.08)	< 0.001
Expt. Desc. Norms (first-order latent)						
Country (observed)	− 0.31 (0.07)	< 0.001	− 0.31 (0.07)	< 0.001	− 0.21 (0.08)	0.005
Sur. Desc. Norms (first-order latent)						
Country (observed)	0.06 (0.09)	0.47	0.04 (0.09)	0.65	0.18 (0.10)	0.09

^a^Objective smoking behavior readings not available for one Northern Irish school and two Colombian schools (excluded from analysis).

## Discussion

Using CFA, our results provide evidence supporting the construct and factorial validity of the two different measurement instruments that were used to elicit social norms for adolescent smoking and vaping as part of the MECHANISMS smoking prevention study (incentivized experiments and a self-report survey; objective 1). Second-order measurement models established that experimental and survey measures of injunctive and descriptive norms were measuring the same underlying second-order latent variable (objective 2). SEM models verified that there was a positive association between higher anti-smoking/vaping norms (the second-order latent variable) and higher self-reported anti-smoking behavior and intentions, and lower objectively measured smoking behavior (objective 4). Therefore our experimental and survey norms measures showed comparable explanatory power related to smoking behavior and intentions following cultural adaptation of the instruments. These findings suggest that our experimental measures of social norms capture the same phenomena as the commonly used self-report survey.

Baseline MIMIC models showed that experimentally elicited *injunctive* norms against smoking/vaping (measured among the school year group), were stronger in Colombia than in NI, but survey responses revealed weaker anti-smoking/vaping injunctive norms in the form of Colombian pupils’ beliefs about their parents, siblings, etc. than among pupils in NI. Colombian pupils also showed weaker *descriptive* anti-smoking/vaping norms in both the experimental and survey measurements. Controlling for latent variable differences, the DIF analyses revealed which individual items were exhibiting measurement invariance for Colombia versus NI (objective 3). After controlling for differences on individual items, differences in experimentally derived injunctive norms and survey descriptive norms were non-significant at the latent variable level. The analysis indicates that the higher anti-smoking/vaping injunctive norms observed for Colombian pupils in their experimental responses were due entirely to differences in the items Part 2 Situation 2 and Part 2 Situation 5. The higher anti-smoking/vaping descriptive norms observed for Colombian pupils in their survey responses were also due entirely to differences in the items Descriptive Norms 2 and Descriptive Norms 3.

Differences in experimental injunctive norms were due to Colombian pupils providing lower social appropriateness ratings for items Part 2 Situation 2 (a parent smoking in their own home in front of children under the age of 5) and Part 2 Situation 5 (in a recent superhero movie the lead actor is seen smoking in the opening scene). Following the implementation of the tobacco control policy in Colombia, it has been found that smoke-free environments have a high acceptability rate among the Bogotá population (85% acceptance)^79^. Therefore, our Colombian participants were potentially showing an awareness of a cultural de-normalization of indoor smoking as a result of this smoke-free environment tobacco control policy when answering Part 2 Situation 2^80^. By comparison, in 2016, one in eight young people reported living in a household with an adult who smokes inside the home in NI^25^. Our NI participants also reported seeing their mothers and fathers smoke more frequently than Colombian pupils, making it more likely that they see adults smoking indoors.

Regarding Part 2 Situation 5, there is considerable literature illustrating how celebrities can impact public health through their influence on knowledge, attitudes and decision-making^81^, and studies have shown a positive association between exposure to movie smoking and adolescent smoking rates^82,83^. In Colombia, the tobacco control policy includes a complete ban on tobacco advertisements, sponsorships, and promotions, and has a high level of implementation in television, cinemas and banners^79^. Moreover, non-paid tobacco product placement in films is not common in Latin America^84^. By comparison, previous research shows high rates of exposure to smoking in television and movies amongst the UK population^85–87^. In 2018, over 80% of adolescents (aged 11–18 years) reported seeing smoking in movies, whilst approximately 70% reported seeing smoking on television^86^.

Colombian pupils provided lower anti-smoking/vaping norms ratings at the latent variable level in their responses to experimental descriptive norms items compared to pupils in NI. Pupils in NI were more likely to estimate that a greater proportion of their school year group would be accepting of a close friend vaping than Colombian pupils (Part 3 Question 2). However, responses to the equivalent smoking item were similar between the two countries. A 2019 report from Public Health England shows that the number of 13–15 year olds who have never smoked but who have tried vaping is increasing in the UK^88^. Adolescents may be drawn towards e-cigarette use due to perceptions that they are safer and healthier than conventional cigarettes, product features (e.g. different flavourings), and marketing^4^. The market for e-cigarettes in Colombia is relatively new (since 2015), and they are not clearly regulated^79^. There is limited evidence regarding the knowledge and access amongst our target population. The UK is one of 20 countries worldwide that classifies certain types of e-cigarettes as medicinal^89^. Potentially, vaping is regarded as more acceptable in the UK as a result.

For the survey injunctive norms scale, the DIF analysis indicated that ratings for the items Injunctive Norms 1 (most of the people who are important to me think that I,… definitely should smoke,…definitely should not smoke) and Injunctive Norms 4 (my brother(s) think(s) that I,… definitely should smoke,… definitely should not smoke) were higher for Colombian versus NI pupils, in the opposite direction to differences at the latent variable level. Potentially the remaining items of the scale, enquiring individually about parents, sisters and friends, do not fully capture the range of individuals Colombian pupils consider to be "important to me". Future researchers may wish to consider expanding this scale to account for all potential influences and cultural differences regarding the socialization of adolescents. Cross-country differences at the latent variable level for survey descriptive norms items became non-significant when the models were adjusted for DIF on items Descriptive Norms 2 and Descriptive Norms 3. Colombian pupils were more likely to report seeing their mothers and fathers smoke less frequently than pupils in NI. In 2018, 14.7% of the UK population aged 18 years and above smoked cigarettes (15.5% NI)^90^. In our NI sample, 17.5% of participants reported having mothers who smoked often or very often (19.6% for fathers). Possibly, smoking rates amongst the parents of our NI participants were higher than the NI adult population in general.

### Strengths and limitations

Strengths of this paper include the large sample size and use of data from schools in two settings with varying normative, cultural and health behavioral traits following a rigorous cultural adaptation of all study instruments. We also examined measurement invariance across relevant subgroups (i.e. between countries) using MIMIC models and DIF analysis, and examined associations with both self-reported and objective measures of smoking behavior. This paper has several limitations. We did not cross-validate our CFA models on an independent sample. However, due to the complexity of our models, we were reluctant to decrease power for our analysis by reducing the sample size. We examined whether data were MCAR (finding evidence that the data were not MCAR) prior to imputing missing data, and are confident that the approach was appropriate^68^. Our results also remained unchanged when repeating analyses without imputing missing data. The MECHANISMS study is funded as a proof of concept study involving a relatively small sample of schools in each country. Therefore, we are cautious in generalizing our findings to other schools in NI and Bogotá (Colombia). There was low power for some items in the DIF analysis. One of our first-order latent variables is measured by two items as our study’s assessment of experimentally derived descriptive norms only consisted of two items. Finally, results should be interpreted with caution due to multiple testing.

### Implications for future research

This paper shows that incentivized experimental methods from the field of behavioral economics^16,17,36^ can be used to measure social norms for smoking and vaping behaviors amongst adolescents in two different settings. It has been proposed that such measures are less prone to bias, providing rich information regarding the distribution of acceptable actions (i.e. norms) and individuals’ norm-following sensitivities that can better explain behavioral heterogeneity *within* and *between* different settings^14,16,36^. Our MIMIC models and DIF analyses indicated when items operated differently from the rest of their scale (e.g. item Injunctive Norms 1 and Injunctive Norms 4). Future researchers may wish to consider amending/deleting such items or expanding the scale before conducting research with children from diverse backgrounds. Our MIMIC models also showed positive associations between personality variables (need to belong, pro-social behavior, openness, extraversion, agreeableness, conscientiousness, and emotional stability) and greater perceived anti-smoking/vaping norms. Therefore, when designing interventions attempting to leverage peer influence to promote smoking prevention amongst adolescents (e.g. the ASSIST programme), interventionists may wish to consider whether certain personality types may be more (or less) suited to transmit anti-smoking/vaping norms^91^. Future research should investigate whether these findings translate to larger, more diverse samples, and different countries.

## Conclusions

The MECHANISMS study was conducted with 11–13 year old school pupils in NI (UK) and 11–15 year olds in Bogotá (Colombia) over a single school semester in 15 schools. This paper contributes evidence supporting the construct validity of incentivized experimental and self-report methods of eliciting injunctive and descriptive social norms for adolescent smoking and vaping behaviors. A second-order CFA model confirmed that the experimental and survey norms measures were measuring the same underlying latent construct of anti-smoking/vaping norms. Thus, we propose that the two methods could be used as complementary measures, to provide a richer understanding of the mechanisms through which social norms influence health-related attitudes and behavior. MIMIC modelling and DIF analyses showed that our norms measurements reflected differences between relevant subgroups of participants (i.e. between two settings varying in smoking rates, culture, and norms). Future research should investigate whether these results vary across repeated measurements and whether they apply in different countries.

## Supplementary information

Supplementary Information.

## Data Availability

The datasets generated during and/or analysed during the current study are available from the corresponding author on reasonable request.

## Code availability

The code used to generate the results reported in the current study are included in the supplementary files.
