# Supplementary material for: Confirmatory factor analysis comparing incentivized experiments with self-report methods to elicit adolescent smoking and vaping social norms
Source: Sci Rep. 2020 Sep 25;10:15818. doi: 10.1038/s41598-020-72784-z (PMC7519107; doi:10.1038/s41598-020-72784-z)
Supplement: Supplementary file 1 — Supplementary Information. [file 41598_2020_72784_MOESM1_ESM.docx]

**Title: Confirmatory factor analysis comparing incentivized experiments with self-report methods to elicit adolescent smoking and vaping social norms.**

Jennifer M. Murray*, PhD, Erik O. Kimbrough, PhD, Erin L. Krupka, PhD, Abhijit Ramalingam, PhD, Rajnish Kumar, PhD, Joanna McHugh Power, PhD, Sharon Sanchez-Franco, MPH, Olga L. Sarmiento, MD, Frank Kee῀, MD, Ruth F. Hunter*῀, PhD

Dr. Jennifer M. Murray, [Jennifer.Murray@qub.ac.uk](mailto:Jennifer.Murray@qub.ac.uk), Centre for Public Health, Institute of Clinical Sciences, Block B, Queen's University Belfast, Royal Victoria Hospital, Grosvenor Road, Belfast, BT12 6BA, 02890958955, Queen's University Belfast, Belfast, UK

Prof. Erik O. Kimbrough, [ekimbrou@chapman.edu](mailto:ekimbrou@chapman.edu), Smith Institute for Political Economy and Philosophy, Chapman University, One University Drive, Orange, CA 92866, Chapman University, Orange, California, United States

Prof. Erin L. Krupka, [ekrupka@umich.edu](mailto:ekrupka@umich.edu), School of Information, University of Michigan, 4322 North Quad, 105 S. State St., Ann Arbor, MI 48109-1285, University of Michigan, Ann Arbor, Michigan, United States

Prof. Abhijit Ramalingam, [abhi.ramalingam@gmail.com](mailto:abhi.ramalingam@gmail.com), Department of Economics, Appalachian State University, 416 Howard Street, ASU Box 32051, Boone, NC 28608, Appalachian State University, Boone, North Carolina, United States

Dr. Rajnish Kumar, [rajnish.kumar@qub.ac.uk](mailto:rajnish.kumar@qub.ac.uk), Queen's Management School, Queen's University Belfast, Riddel Hall, 185 Stranmillis Road, Belfast, BT9 5EE, Queen's University Belfast, Belfast, UK

Dr. Joanna McHugh Power, [Joanna.MchughPower@mu.ie](mailto:Joanna.MchughPower@mu.ie), Department of Psychology, Maynooth University, Maynooth, Co. Kildare, Ireland

Ms. Sharon Sanchez-Franco, sc.sanchez@uniandes.edu.co, School of Medicine, University of the Andes, Carrera 1 No 18 A – 10, Bloque Q Piso 8, Bogotá, Colombia, Postal Code: 57, University of the Andes, Bogotá, Colombia

Prof. Olga L. Sarmiento, [osarmien@uniandes.edu.co](mailto:osarmien@uniandes.edu.co), School of Medicine, University of the Andes, Carrera 1 No 18 A – 10, Bloque Q Piso 8, Bogotá, Colombia, Postal Code: 57, University of the Andes, Bogotá, Colombia

Prof. Frank Kee, [f.kee@qub.ac.uk](mailto:f.kee@qub.ac.uk), Centre for Public Health, Institute of Clinical Sciences, Block A, Queen's University Belfast, Royal Victoria Hospital, Grosvenor Road, Belfast, BT12 6BA, Queen's University Belfast, Belfast, UK

Dr. Ruth F. Hunter, [ruth.hunter@qub.ac.uk](mailto:ruth.hunter@qub.ac.uk), Centre for Public Health, Institute of Clinical Sciences, Block B, Queen's University Belfast, Royal Victoria Hospital, Grosvenor Road, Belfast, BT12 6BA, 02890958944, Queen's University Belfast, Belfast, UK

*Corresponding authors; ῀Joint last author with equal contribution.

**Supplement 1: Study procedures, study flow diagram, baseline characteristics, and glossary of terms**

**Procedures**

A parental opt-out procedure was used with all participants providing their informed consent. Pupils who consented to participate took part in a baseline assessment consisting of a series of game theory experiments and completion of a self-report survey. Following the baseline assessment, each school took part in one of two previously tested smoking prevention interventions: ASSIST^1–3^ (N=4 schools in Northern Ireland [NI], N=4 Bogotá) or Dead Cool^4–6^ (N=3 NI, N=4 Bogotá) over a single school semester (approximately 10 weeks). Briefly, the ASSIST intervention is designed to train the most influential pupils in the year group, nominated in a Peer Questionnaire completed by all participants prior to the baseline assessment, to use informal contacts with their peers (i.e. other pupils in their school year group) to encourage them not to smoke. Dead Cool is underpinned by more conventional classroom pedagogy, including training of school teachers in programme delivery and provision of programme resources (lesson plans, pupil work books, fact sheets and a DVD) to enhance pupils' knowledge of potential influences on smoking behavior from family, friends and the media. Following intervention delivery in each school, all participants took part in a follow-up assessment, again completing the game theory experiments and a self-report survey. Prior to implementation in Colombia, all study materials (experiments, surveys, intervention materials) underwent a 'cultural adaptation' process, including translation into Spanish language, using a previously published framework.^7,8^ Participation in the study's experiments required a monetary payment to be made to each individual pupil. In NI the payment was made in cash, however due to Colombian ethical regulations the payment was made using gift cards to pupils in Bogotá.

Ethics approval was granted from the School of Medicine, Dentistry and Biomedical Sciences Ethics Committee at Queen's University Belfast (QUB) on September 21, 2018 and from the Research Ethics Committee at Universidad de los Andes (Uniandes) on September 17, 2018.

**Data collection**

The baseline assessment consisted of two separate sessions, held approximately one week apart, with each class in the school year group in each school. Sessions lasted approximately 50 minutes. Participating pupils completed the game theory experiments during the first session and the self-report survey during the second session. Experiments and surveys were collected on iPads using the platform Qualtrics (web-based platform in NI and offline version in Bogotá) (Qualtrics, Provo, Utah, USA). At the start of each session, participants were assured that any information provided would be treated as confidential. They were also instructed not to communicate with other participants and to direct any questions to a researcher. In NI, poster boards were used at iPad stations to discourage communication between participants. In both countries, instructions were delivered onscreen with key portions read aloud by the experimenter. The experimenter read out introductory instructions at the start of the experiment, and at the start of Parts 1, 2 and 4. Pupils were invited to ask any questions. Dummy screens were inserted at the end of Parts 1 and 3 instructing pupils to wait until all of their classmates were ready to proceed to the next part so that instructions could be read together. Parts 2 and 3 were otherwise self-paced, and pupils were invited to raise their hand to have any further questions answered privately. The same procedures were used during the follow-up assessment.

**Game Theory Experiments**

The game theory experiments consisted of a series of incentivized tasks which were based on published works in the field of behavioral economics,^9–11^ and designed by the original producers (Kimbrough, Krupka) and other experts in the field (Kumar, Ramalingam). There were four parts to the experiment: (1) a Rule-Following (RF) task measuring each individual participant's sensitivity to the effects of social norms; (2) a series of coordination games attempting to elicit *injunctive* social norms unrelated and related to smoking and vaping behaviors; (3) a series of coordination games attempting to elicit *descriptive* social norms related to smoking and vaping behaviors; (4) a willingness-to-pay task designed to measure each individual participant's support for cultivating anti-smoking norms. These are outlined in more detail below. The current paper uses data from Parts 1-3. The full experimental protocol is available in supplement 2.

At the start of experimental sessions, participants were informed that they would receive a participation fee of £5.00 (NI; *COP* $5.000 in Bogotá), and that they could earn money in each part of the experiment (maximum £35 in NI, *COP* $50.000 in Bogotá) depending on the answers they provided and those provided by other pupils in their year group. They were told that the researchers would determine their payment by performing two sets of randomizations for each part of the experiment: (1) to determine whether payment was based on answers provided at baseline or follow-up; (2) to determine which question of each part would result in payment.

***Part 1: Identifying individual-level norms sensitivity***

Part 1 of the experiment consisted of an individual decision task (a variant of the RF Task)^10,11^ measuring participants' preferences for following established rules and social norms, without peer interaction. The task instructs participants to follow an explicitly stated arbitrary rule when doing so provides them with no monetary benefits, but actually imposes explicit monetary costs directly proportional to the degree of rule-following. We employed the version of the RF task introduced by Kimbrough and Vostroknutov (2018).^10^ Specifically, participants were asked to sequentially allocate 50 balls across two buckets (one blue and one yellow). They were told that "The rule is to put the balls in the blue bucket". They were also informed that they would receive £0.05 (NI; *COP* $100 Bogotá) for every ball they put in the blue bucket and £0.10 (NI; *COP COP* $200 Bogotá) for every ball they put in the yellow bucket. Lastly, they were informed that they would be given five minutes to allocate the 50 balls between the two buckets and that any balls which were not allocated by the end of the five minutes were worth nothing.^[[1]](#footnote-1)^ No other information was provided. Therefore, assuming a participant allocated all 50 balls, the minimum amount that he/she could earn was £2.50 (NI; *COP* $5.000 Bogotá) if he/she followed the rule completely and allocated all 50 balls to the blue bucket. The maximum amount that could be earned was £5.00 (NI; *COP* $10.000 in Bogotá) if he/she completely ignored the rule and allocated all 50 balls to the yellow bucket. The central premise is that the more a participant cares intrinsically about rule-following the more willing he/she will be to incur the costs of doing so.^11^ The extent of rule-following in the RF task provides a measure of individual norm-following proclivity, and this norm sensitivity measure has been shown to correlate with willingness to follow norms of cooperation, reciprocity and prosocial behavior across decision contexts.^11^ To avoid introducing any potential biases due to preference for bucket placement, participants were randomized to a version of the RF task with the blue bucket on the left (n=778), or a version with the blue bucket on the right (n=798).

***Parts 2-3: Measuring injunctive and descriptive social norms***

Parts 2 and 3 of the experiment consisted of a series of incentivized coordination games which used methods employed by Krupka and Weber^9^ to elicit injunctive and descriptive social norms around smoking and vaping. Injunctive norms reflect shared beliefs among members of a population about what actions people *ought* *to* take; descriptive norms reflect shared beliefs among members of a population about what actions people *actually do* take.^9^

In Part 2, participants were asked to rate the social appropriateness of various actions that others might take on a six-point Likert scale: "extremely socially inappropriate", "very socially inappropriate", "somewhat socially inappropriate", "somewhat socially appropriate", "very socially appropriate", "extremely socially appropriate". Situation 1 aimed to elicit pro-sociality injunctive norms by asking participants to co-ordinate with others in their year group to rate the social appropriateness of a range of actions one might take in a standard Dictator game. The Dictator game is commonly used as a measure of social preferences, in particular, altruism.^12^ Such norms are unlikely to be affected by interventions targeted at altering smoking behavior. Eight items (situations 2-9) were asked to assess smoking- and vaping-related injunctive social norms. In Part 3, participants were asked to estimate the proportion of peers in their school year group who would be accepting of certain behaviors on a six-point Likert scale: "none of my peers", "only a few of my peers", "some of my peers", "a lot of my peers", "most of my peers", "all of my peers". Two items were asked to assess smoking- and vaping-related descriptive social norms.

The principal feature of this part of the experiment is that participants are provided with incentives to *match* their ratings/estimates to other participants' in their school year group on the day as opposed to providing personal opinions. For example, participants are informed that they will receive £10 (NI; *COP* $15.000 Bogotá) if the answer they provide for a randomly selected question matches the most common answer in the school year group. Assuming that a norm exists, and in the absence of peer interaction, participants attempting to match others' responses in order to win the incentive will anticipate the extent to which others will rate an action as socially appropriate or inappropriate (or anticipate the extent to which others will estimate that a large or small proportion of their peers would be accepting of certain behaviors), and respond accordingly.^[[2]](#footnote-2)^ Therefore, in Part 2 of the experiment participants play a coordination game in which the incentive elicits an empirical measure of injunctive social norms as collective perceptions of the social appropriateness of various behaviors. In Part 3 they play a coordination game in which the incentive elicits an empirical measure of descriptive social norms as collective perceptions of the rate of acceptance of certain behaviors.

As proposed by the original authors, the components elicited in Parts 1-3 of the experiment can be examined within the context of a norm-dependent utility framework to further our understanding of how the existence of social norms, and individuals' norms sensitivities, can influence behavior in social settings.^9–11^ Within this framework, behavioral heterogeneity in a given social context is proposed as being related to the fact that people suffer disutility from violating norms and that those individuals differ in sensitivity to own-norm violations.

u(a_k_) = V {ᴫ(a_k_)} + γN(a_k_) (1)

In the above framework, social norms are modelled quantitatively, such that a decision maker’s "pay-off", u(a_k_), from each action, V {ᴫ(a_k_)}, is traded off against the normative appropriateness of each action according to the parameter γ≥0, representing the degree to which the individual cares about adhering to social norms, with the function N capturing the social norm. N_g_(a_k_) denotes the social norms for group g, which are estimated from the coordination games in Parts 2 and 3 of the experiment. γ is the parameter reflecting individual sensitivity to the norm, estimated using the total number of balls allocated to the blue "rule-following" bucket in the RF task of Part 1.

***Part 4: Measuring willingness to pay to support anti-smoking norms***

Part 4 of the experiment gives us a chance to test the implications of this model. Participants are given an endowment of £5 (NI; *COP* $10.000 Bogotá) and asked to decide how much of the £5 to donate to the organizations responsible for ASSIST/Dead Cool, depending on which programme their school was taking part in, and how much to keep for themselves. They are provided with a description of ASSIST/Dead Cool as "a smoking prevention programme which aims to prevent the uptake of smoking among adolescents your age". Therefore, in the same way that a willingness to incur a cost to follow the rule in the RF task reveals a respect for following norms in general, the extent of a participant's willingness to incur a cost to make a higher donation to a programme whose aim is to encourage smoking reduction by others reveals their support for anti-smoking norms. Since a donation may be taken as revealing a participant's belief that such smoking prevention programmes are normatively appealing and effective, this task may be taken as providing evidence for the behavioral impact of an injunctive anti-smoking social norm.

To connect this to the model (1), we need only assume that V is increasing in the participant’s own payoff; then when facing the decision about whether to donate to the anti-smoking intervention charity, subjects trade off their own higher payoff from keeping the money for themselves against the normative appropriateness of donating to help prevent smoking. The theory implies that when norms are stronger, or an individual’s γ is larger, the amount donated will be higher.

Further details on the smoking- and vaping-related scenarios assessed in Parts 2 and 3 of the experiment and numerical coding of responses are provided in table 1 of the manuscript. Responses to the experiment items from Part 2 were coded such that numerical responses ran between -1 (extremely socially inappropriate) and +1 (extremely socially appropriate) following procedures adopted in Krupka and Weber (2013).^9^ Similarly, responses to experiment items from Part 3 were coded such that numerical responses ran between -1 (none of my peers) and +1 (all of my peers).

1. Campbell, R. *et al.* An informal school-based peer-led intervention for smoking prevention in adolescence (ASSIST): a cluster randomised trial. *Lancet (London, England)* **371**, 1595–1602 (2008).

2. Starkey, F., Moore, L., Campbell, R., Sidaway, M. & Bloor, M. Rationale, design and conduct of a comprehensive evaluation of a school-based peer-led anti-smoking intervention in the UK: the ASSIST cluster randomised trial. *BMC Public Health* **5**, 43 (2005).

3. Audrey, S., Cordall, K., Moore, L., Cohen, D. & Campbell, R. The development and implementation of a peer-led intervention to prevent smoking among secondary school students using their established social networks. *Health Educ. J.* **63**, 266–284 (2004).

4. Dunne, L., Thurston, A., Gildea, A., Kee, F. & Lazenbatt, A. Protocol: A randomised controlled trial evaluation of Cancer Focus NI’s ‘Dead Cool’ smoking prevention programme in post-primary schools. *Int. J. Educ. Res.* **75**, 24–30 (2016).

5. Thurston, A. *et al.* *A randomised controlled trial evaluation of Cancer Focus Northern Ireland’s ‘Dead Cool’ smoking prevention programme in Northern Ireland post-primary schools*. (2016).

6. Thurston, A. *et al.* A randomized controlled efficacy trial of a smoking prevention programme with Grade 8 students in high schools. *Int. J. Educ. Res.* **93**, 23–32 (2019).

7. Barrera, M. & Castro, F. G. A heuristic framework for the cultural adaptation of interventions. *Clin. Psychol. Sci. Pract.* **13**, 311–316 (2006).

8. Barrera, M., Castro, F. G., Strycker, L. A. & Toobert, D. J. Cultural adaptations of behavioral health interventions: a progress report. *J. Consult. Clin. Psychol.* **81**, 196–205 (2013).

9. Krupka, E. L. & Weber, R. A. Identifying social norms using coordination games: why does dictator game sharing vary? *J. Eur. Econ. Assoc.* **11**, 495–524 (2013).

10. Kimbrough, E. O. & Vostroknutov, A. A portable method of eliciting respect for social norms. *Econ. Lett.* **168**, 147–150 (2018).

11. Kimbrough, E. O. & Vostroknutov, A. Norms Make Preferences Social. *J. Eur. Econ. Assoc.* **14**, 608–638 (2016).

12. Levitt, S. D. & List, J. A. What do laboratory experiments measuring social preferences reveal about the real world? *J. Econ. Perspect.* **21**, 153–174 (2007).

13. Prinsen, C. A. C. *et al.* How to select outcome measurement instruments for outcomes included in a “Core Outcome Set” – a practical guideline. *Trials* **17**, 449 (2016).

14. Bicchieri, C., Muldoon, R. & Sontuoso, A. ‘Social norms’. in *The Stanford Encyclopedia of Philosophy* (ed. Zalta, E. N.) (2018).

**Study flow diagram**

**CONSORT 2010 Flow Diagram**

Left the school (n=9)

Non-attendance to school (n=8)

Withdrawn (n=2)

Participated in follow-up (n=678)

(n=281 NI, n=397 Bogotá)

Participated in follow-up (n=924)

(n=458 NI, n=466 Bogotá)

Received **Dead Cool** intervention (n=690)

(n=285 NI, n=405 Bogotá)

Received **ASSIST** intervention (n=962)

(n=480 NI, n=482 Bogotá)

Selected (N=15)

(N=7 NI, N=8 Bogotá)

Entered the school (n=7)

## Follow-Up

Entered the school (n=0)

Left the school (n=9)

Non-attendance to school (n=17)

Withdrawn (n=12)

**Excluded (n=56)**

♦  Declined to participate (n=52)

♦  Left the school (n=3)

♦  No response (n=1)

**Excluded (n=109)**

♦  Declined to participate (n=76)

♦  Left the school (n=21)

♦  No response (n=12)

Assessed for eligibility

(n=746 children)

Assessed for eligibility

(n=1071 children)

Allocated to **Dead Cool** intervention (N=7)

(N=3 NI, N=4 Bogotá)

Allocated to **ASSIST** intervention (N=8)

(N=4 NI, N=4 Bogotá)

## Allocation

**Excluded (N=57)**

♦  Declined to participate (N=6)

♦  No answer (N=18)

♦  Unsuitable (N=28)

♦  Offered alternative (N=5)

Assessed for eligibility

(N=72 schools)

## Enrollment

## Analysis

**Analysed (n=687)**

♦  Experiment and survey (n=646)

♦  Experiment only (n=25)

♦  Survey only (n=16)

**Analysed (n=949)**

♦  Experiment and survey (n=867)

♦  Experiment only (n=38)

♦  Survey only (n=44)

**Baseline pupil characteristics for MECHANISMS schools. Mean (SD) unless otherwise stated**

|  | **Northern Ireland (N=7)** | **Colombia (N=8)** | **All schools (N=15)** |
| --- | --- | --- | --- |
| Intervention, N |  |  |  |
| *ASSIST schools* | 4 | 4 | 8 |
| *Dead Cool schools* | 3 | 4 | 7 |
| No. of classes, N | 36 | 32 | 68 |
| No. of pupils, n | 825 | 999 | 1824 |
| Participation, n (%) | 764 (92.6%) | 892 (89.3%) | 1656 (90.8%) |
| School MDM (1-890)^a^ | 355 (219) |  |  |
| School INSE (1-4)^b^ |  | 2.4 (0.5) |  |
| Individual MDM (1-890)^a^ | 366 (238) |  |  |
| Individual DANE SES (0-6)^c^ |  | 2.1 (0.9) |  |
| Sex, n(%) |  |  |  |
| *Boys* | 335 (43.8%) | 436 (48.9%) | 771 (46.6%) |
| *Girls* | 355 (46.5%) | 431 (48.3%) | 786 (47.5%) |
| *Prefer not to say* | 11 (1.4%) | 5 (0.6%) | 16 (1.0%) |
| Age, n (%) |  |  |  |
| *11 years old* | 1 (0.1%) | 26 (2.9%) | 27 (1.6%) |
| *12 years old* | 279 (36.5%) | 320 (35.9%) | 599 (36.2%) |
| *13 years old* | 414 (54.2%) | 313 (35.1%) | 727 (43.9%) |
| *14 years old* | 7 (0.9%) | 146 (16.4%) | 153 (9.2%) |
| *15 or more years old* | - | 77 (8.6%) | 77 (4.6%) |
| Ethnicity, n (%)^d^ |  |  |  |
| *White British* | 171 (22.4%) | - | 171 (10.3%) |
| *White Irish* | 474 (62.0%) | - | 474 (28.6%) |
| *Colombian: No ethnic minority* | - | 753 (84.4%) | 753 (45.5%) |
| *Ethnic minority* | 54 (7.1%) | 119 (13.3%) | 173 (10.4%) |
| Family |  |  |  |
| *Lives with mother, n (%)* | 677 (88.6%) | 747 (83.7%) | 1424 (86.0%) |
| *Lives with father, n (%)* | 529 (69.2%) | 443 (49.7%) | 599 (36.2%) |
| *Number of family members in household* | 2.6 (0.7) | 2.6 (0.9) | 2.6 (0.8) |

^a^Multiple deprivation measure (NI only; 1=most deprived to 890=least deprived). Calculated based on ranking of NI postcodes for seven domains of deprivation including income; employment; health and disability; education, skills and training; access to services; living environment; crime and disorder. Provided by Northern Ireland Statistics and Research Agency (NISRA).

^b^Socio-economic level index (Bogotá only; 1=Lower; 2=Middle-low; 3=Middle-high; 4=Higher). Calculated each year using a sample from each school, based on the characteristics of the home and its infrastructure, some household assets, the relationship of the children with their parents, among other characteristics. Schools are then classified into four levels according to the average of the responses of the pupils enrolled in them. Provided by the Instituto Colombiano para el Fomento de la Educación Superior (ICFES; “Colombian Institute for the Promotion of Higher Education”).

^c^Socioeconomic level index of individual pupils (Bogotá only; 0=Informal settlement; 1=Lowest; 2=Low; 3=Middle-Low; 4=Middle; 5=Middle-High; 6=High), according to the Departamento Administrativo Nacional de Estadística (DANE; "National Administrative Department of Statistics").

^d^White Irish, White British (NI only); Colombian: No ethnic minority (Bogotá only).

**Glossary of terms**

**Akaike Information Criterion (AIC):** A parsimony fit index. These statistics are generally used when comparing non-nested or non-hierarchical models estimated with the same data and indicates to the researcher which of the models is the most parsimonious. Smaller values suggest a good fitting, parsimonious model however because these indices are not normed to a 0-1 scale it is difficult to suggest a cut-off other than that the model that produces the lowest value is the most superior.

**Bayesian Information Criterion (BIC):** The BIC and adjusted BICs are parsimony fit indices, closely related to the AIC, which impose a greater penalty term for the number of parameters in the model. Smaller values suggest a good fitting, parsimonious model.

**Chi-square statistic:** The chi-square statistic can be used to assess the absolute fit of the model to the data, assuming correct model specification. A non-significant result (p>0.05) indicates good model fit. However, it can be overly influenced by sample size, correlations, variance unrelated to the model, and multivariate non-normality.

**Comparative Fit Index (CFI):** The CFI compares the chi-square statistic from the specified model with the chi-square statistic from the null model, in which all of the variables are uncorrelated. Values range between 0 and 1. CFI values of ≥0.96 indicate good model fit.

**Confirmatory factor analysis (CFA):** CFA is a statistical technique to determine whether measures of a construct are consistent with a researcher’s understanding of the nature of the construct, or factor, by testing whether the data fits a hypothesized measurement model. Model fit is assessed by reference to a number of goodness-of-fit indices.

**Construct validity:** Construct validity refers to the extent to which inferences from test scores can be made in relation to the construct of interest and is determined based on integration of any evidence with a bearing on interpretation or meaning of test scores. Some methods of investigating construct validity include appraising theoretically expected relationships among individual items, between test scores and other measures (including external criteria, e.g. by comparison with a 'gold standard' or other outcome measure expected to show an association with the construct) or comparing scores between groups expected to differ on the underlying construct.

**Coordination game:** A coordination game is a type of simultaneous game used in game theory when players benefit from coordinating their activities by making the same decisions. In the MECHANISMS study, coordination games are played in Parts 2 (injunctive norms) and 3 (descriptive norms) of the incentivized norms elicitation experiments, in which participants are asked to ‘coordinate’ with others in their school year group to rate the social appropriateness of various smoking-related activities (Part 2) or the proportion of their school year group who would be accepting of a close friend smoking or vaping (Part 3). To encourage them to think about the social norm instead of providing personal opinions, they are informed that they will receive a cash (or cash equivalent) payment if the answer they provide to a randomly selected question matches the most common answer provided in the school year group on the day.

**Descriptive norms:** Descriptive norms reflect shared beliefs among members of a population about what actions people *actually do* take.

**Differential item functioning (DIF):** DIF occurs when an item on a test or questionnaire has different measurement properties for one group versus another, irrespective of mean differences on the overall latent construct. Individual items can be tested for DIF by regressing them onto the grouping variable whilst controlling for differences at the latent variable level.

**Expected parameter change (EPC):** The EPC is associated with the modification index, and shows the predicted value of the freely estimated parameter.

**Factorial validity:** Factorial validity examines the extent to which the underlying putative structure of a scale is recoverable in a set of test scores.

**Full Information Maximum Likelihood (FIML):** A method to deal with missing data. It estimates parameters directly using all the information that is already contained in the incomplete data set. FIML obtains parameter estimates by maximizing the likelihood function of the incomplete data.

**Game theory:** Game theory is a branch of behavioral economics that has developed well-defined mathematical models for describing and understanding cooperation and competition amongst individuals and groups.

**Incentivized experiments:** Methods derived from the field of behavioral economics to elicit normative beliefs and norm-sensitivities using cash (and cash equivalent) payments.

**Injunctive norms:** Injunctive norms reflect shared beliefs among members of a population about what actions people *ought to* take.

**Missing completely at random (MCAR):** A type of mechanism causing missing data. If the mechanism causing missing data depends neither on observed data nor on missing data, the data are said to be MCAR. MCAR causes enlarged standard errors due to the reduced sample size, but does not cause bias (‘systematic error’ that is overestimation of benefits and underestimation of harms). In this situation, the incomplete datasets are representative for the entire dataset.

**Modification index (MI):** MIs correspond to a reduction in the chi-square value (absolute model fit statistic) when a specific parameter is freed. A reduction of 3.84 with one degree of freedom represents a significant improvement in absolute model fit.

**Multiple indicators multiple causes (MIMIC):** MIMIC models can be used to examine differences on latent variables by regressing them onto an observed grouping variable.

**Normative beliefs:** Normative beliefs refer to the perceived behavioral expectations of important referent individuals or groups.

**Norm-sensitivity:** Individuals’ norms sensitivities represent the degree to which they experience utility or disutility from norm violations or gain from norm adherence.

**Root Mean Square Error of Approximation (RMSEA):** The RMSEA compares the observed sample covariance matrix with the hypothesized covariance model. It indicates how well the model, with unknown but optimally chosen parameter estimates would fit the sample covariance matrix. The RMSEA favors parsimony and will choose the model with the least number of parameters. It is also possible for a confidence interval to be calculated around its value. RMSEA values of ≤0.06 indicate good model fit.

**Social desirability bias:** Social desirability bias is a type of response bias that is the tendency of survey respondents to answer questions in a manner that will be viewed favorably by others (e.g. researchers collecting data).

**Social norms:** Individuals’ beliefs regarding the actions and beliefs of others in a reference group.

**Standardized Root Mean Square Residual (SRMR):** The SRMR is the square root of the difference between the residuals of the sample covariance matrix and the hypothesized covariance model. It is standardized to range from 0-1. SRMR values of ≤0.09 indicate good model fit.

**Structural equation modelling (SEM):** A broad framework for data analysis which permits testing of hypotheses regarding the structure of relationships between a set of variables.

**Tucker-Lewis Index (TLI):** The TLI, or Non-Normed Fit Index (NNFI), is related to the CFI, comparing the chi-square statistic from the specified model with the chi-square statistic from the null model, in which all of the variables are uncorrelated. The TLI overcomes the issue of sensitivity to sample size that occurs with the CFI, which underestimates fit for samples less than 200. The TLI prefers simpler models, and due to its non-normed nature, values outside of the range 0-1 are possible. TLI values of ≥0.95 indicate good model fit.

**Supplement 2: English and Spanish language versions of the experimental protocol**

What is your name and your form class?

First name ________________________________________________

Surname ________________________________________________

Form class ________________________________________________


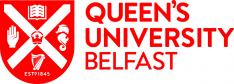


**Experimental Instructions**

**General information**

This is a study about decision-making. You will be paid a fee of £5 for taking part, as outlined below. In addition, you may receive some extra money based on your choices and the choices made by others during the study.

If you have any questions during the session, please raise your hand and wait for a researcher to come to you.  Please do not talk or try to communicate with other participants during the experiment.  It is important that everyone taking part makes his or her own decisions.

This is an on-going study, which has received funding from the UK Medical Research Council to cover all current and future costs. You can be certain that all participants who complete the study will be paid as described in the instructions. If you have any concerns, please contact:

**Dr. Ruth Hunter**
Centre for Public Health/UKCRC Centre of Excellence for Public Health (NI)
School of Medicine, Dentistry and Biomedical Sciences
Institute of Clinical Science B, Royal Victoria Hospital
Grosvenor Road, Belfast, BT 12 6BJ
E-mail: ruth.hunter@qub.ac.uk; 
Tel: +44 (0) 28 90978944
 
**There are four parts to today’s study.**

**You can earn money in each part.**

Your earnings from today will **not** be paid to you today. We will come back to your school at the end of the programme in ten weeks’ time. At that time, we would like you to participate in another study. There will be four parts to that study, and you can earn money in each part of that study too.

After you have participated in the study at the end of the program we will determine for each part whether you receive earnings from today or from the study at the end of the programme. For each part, we will toss a coin to determine this. We will record your choices in both today’s study and the study at the end of the program. You will be able to review your choices from both experiments when you learn your payment, if you wish.

**Part 1**

In Part 1 of this study, you will decide how to allocate 50 balls between two buckets. Your task is to put each of the balls, one-by-one, into one of the two buckets: the blue bucket or the yellow bucket. The balls will appear to the left-hand side of your screen, and you can allocate each ball by clicking and dragging it to the bucket of your choice. For each ball you put in the blue bucket, you will receive 5 pence, and for each ball you put in the yellow bucket, you will receive 10 pence.

The rule is to put the balls in the blue bucket.

Once the experiment begins, you will have 5 minutes to put the balls into the buckets. When you are finished, please click on the next button and wait quietly for further instructions from the experimenter. Any balls that have not been placed in a bucket at the end of the 5 minutes are worth nothing. Your earnings from Part 1 will be based on your decisions: it is the sum of earnings from the blue and yellow buckets.

This is the end of the instructions for Part 1. If you have any questions, please raise your hand and a researcher will answer them privately. Otherwise, please wait quietly until all of your classmates are ready and click on the next button to begin the experiment.


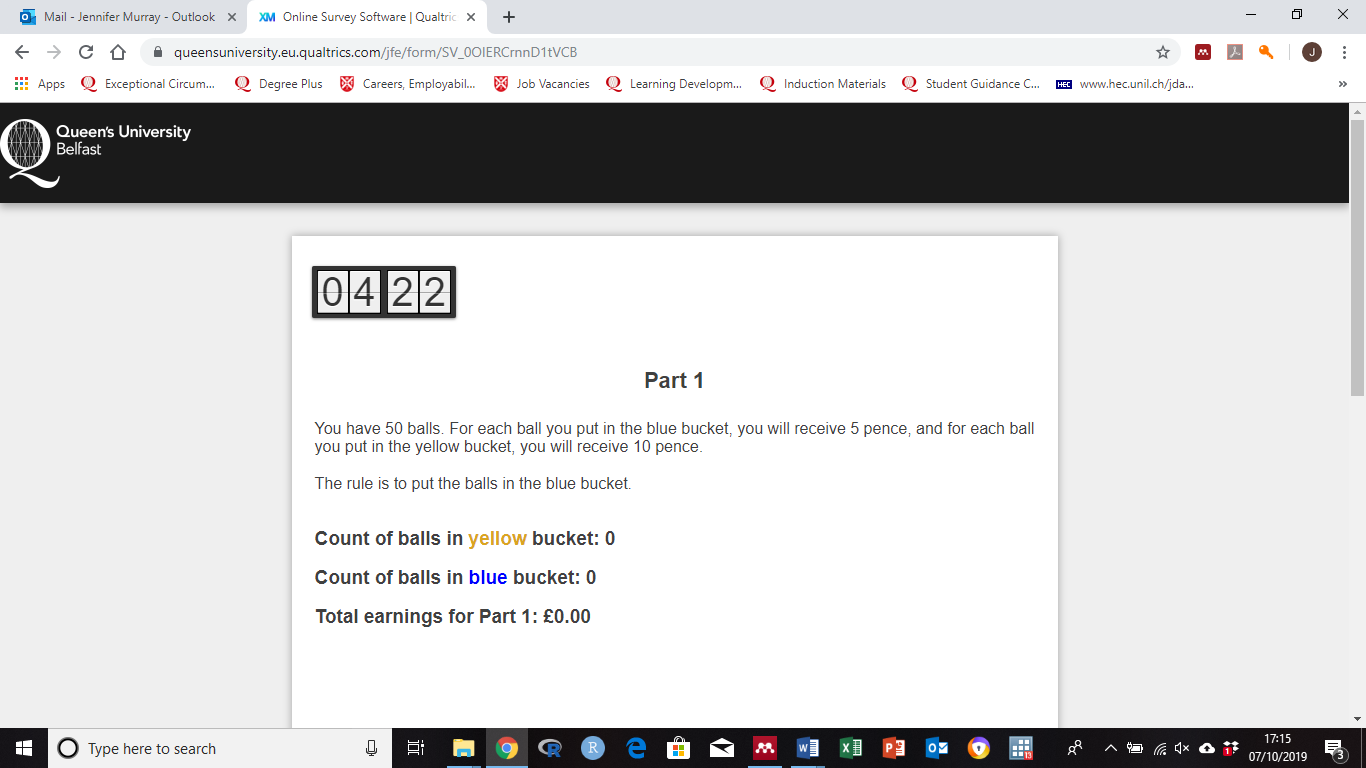


**The 50 balls can be re-located individually to either the blue or yellow bucket by mouse click and drag-and-drop.**

**Updated as balls are dragged in or out of the yellow bucket.**

**Updated as balls are dragged in or out of the blue bucket.**

**Timer indicating five-minute count-down for completing Rule-Following task.**


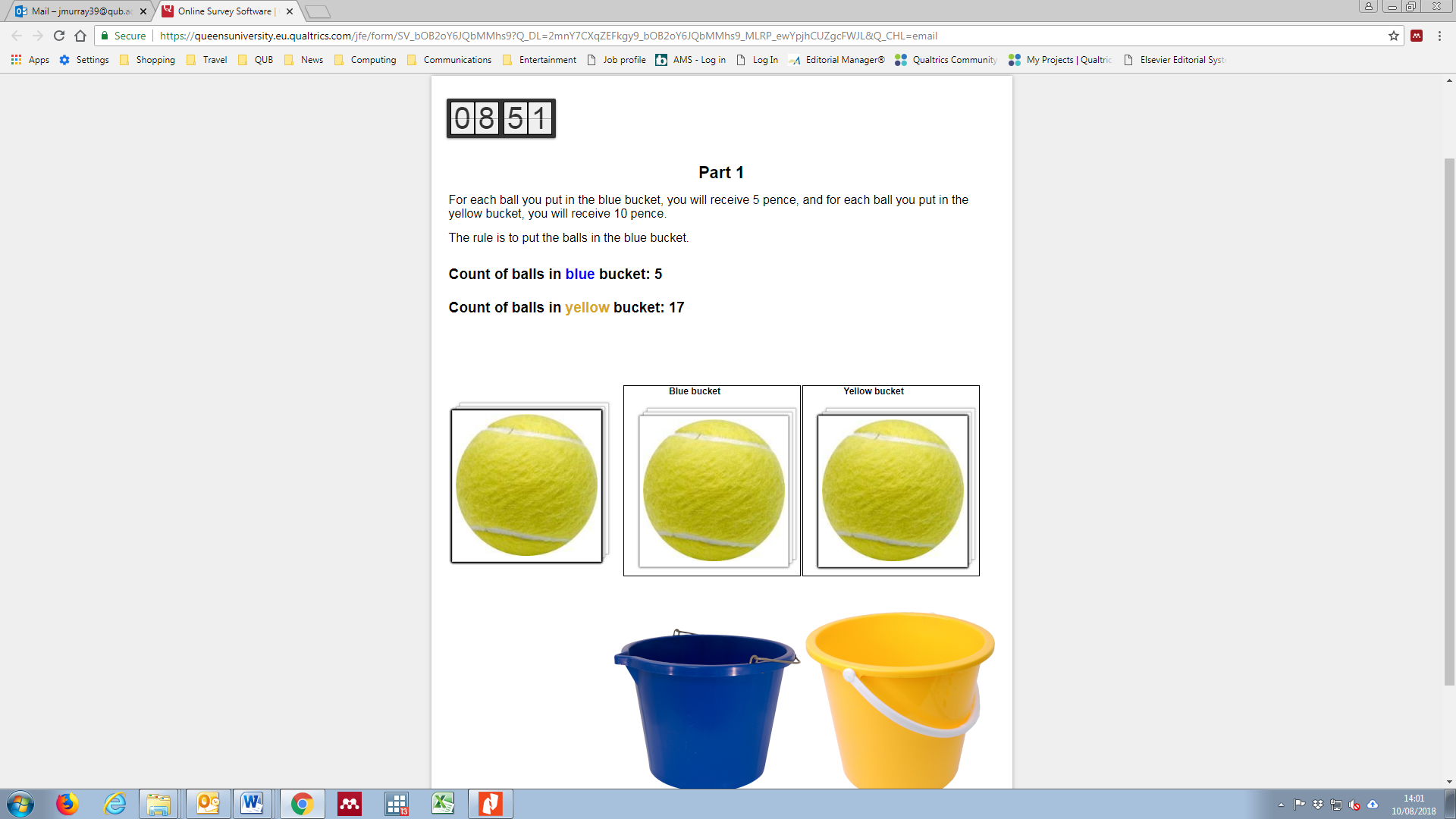


**N.B. Participants were randomised to this version of the experiment or to a version that had the buckets in reverse order to overcome any potential bias due to positioning of buckets.**

**Part 2**

On the following screens, you will read descriptions of a series of situations. These descriptions correspond to situations in which one person must make a decision or has taken an action. For each situation, you will be given a description of the decision faced or action taken by this person.

After you read the description of the situation, you will be asked to evaluate the decision or action taken. You will be asked to decide whether taking that decision or action would be "socially appropriate" and "consistent with moral or proper social behaviour" or "socially inappropriate" and "inconsistent with moral or proper social behaviour". By socially appropriate, we mean behaviour that most people in your school year group agree is the "correct" or "ethical" thing to do. Another way to think about what we mean is that if the person in the situation were to select a socially inappropriate choice, then someone else in your school year group might be angry with that person for doing so.

In each of your responses, we would like you to answer as truthfully as possible, based on your opinions of what constitutes socially appropriate or socially inappropriate behaviour.

To give you an idea of how the experiment will proceed, we will go through an example and show you how you will indicate your responses. On the next screen you will see an example of a situation.

**Part 2**

**Example Situation**

A person is at a local coffee shop near school. While there, the person notices that someone has left a wallet at one of the tables. The person must decide what to do. This person has four possible choices: take the wallet, ask others nearby if the wallet belongs to them, leave the wallet where it is, or give the wallet to the shop manager. The person can choose one of these four options.

The table below presents a list of the possible choices available to this person. For each of the choices, you will be asked to indicate whether you believe choosing that option is extremely socially inappropriate, very socially inappropriate, somewhat socially inappropriate, somewhat socially appropriate, very socially appropriate, or extremely socially appropriate. To indicate your response, you would select the corresponding option.

**The person's choice...**

|  | Extremely socially inappropriate | Very socially inappropriate | Somewhat socially inappropriate | Somewhat socially appropriate | Very socially appropriate | Extremely socially appropriate |
| --- | --- | --- | --- | --- | --- | --- |
| Take the wallet |  |  |  |  |  |  |
| Ask others nearby if the wallet belongs to them |  |  |  |  |  |  |
| Leave the wallet where it is |  |  |  |  |  |  |
| Give the wallet to the shop manager |  |  |  |  |  |  |

**Please make sure that you have placed one tick in each row.**

If this were one of the situations for this study, you would consider each of the possible choices above and, for that choice, indicate the extent to which you believe taking that action would be socially appropriate" and "consistent with moral or proper social behaviour" or "socially inappropriate" and "inconsistent with moral or proper social behaviour".  Recall that by socially appropriate we mean behaviour that most people agree is the "correct" or "ethical" thing to do.

**Part 2**

For example, suppose you thought that taking the wallet was extremely socially inappropriate, asking others nearby if the wallet belongs to them was somewhat socially appropriate, leaving the wallet where it is was somewhat socially inappropriate, and giving the wallet to the shop manager was extremely socially appropriate.  Then you would indicate your responses as follows:


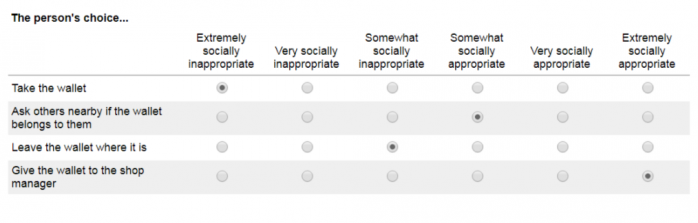
Are there any questions about this example situation or about how to indicate your responses?  On the following pages, there are several situations, all dealing with decisions that another person might have to make. 

You will indicate your appropriateness rating by selecting the corresponding option.

At the end of the experiment today, we will randomly select one of the situations. For this situation, we will also randomly select one of the possible choices that Individual A could make. Thus, we will select both a situation and one possible choice at random. For the choice selected, we will find out which response was selected by most people in your school year group today. 

If you give the same response as that most frequently given by other people in your school year group, then your earning from Part 2 will be £10.  This amount will be paid to you, in cash, at the conclusion of the study in ten weeks. For instance, if we were to select the example situation above and the possible choice "Leave the wallet where it is", and if your response had been "somewhat socially inappropriate", then your earning from Part 2 would be £10, if this was the response selected by most other people in your school year group today. Otherwise your earning from Part 2 would be £0.

You are now going to complete some similar questions to this example on your own. You can go at your own pace.

***If you have any questions from this point on, please raise your hand and wait for the researcher to come to you.***

**Part 2**

**Situation 1**

Consider two hypothetical individuals from your school year group – Individual A and Individual B. Suppose that Individual A is randomly paired with another person in your school year group, Individual B in an experiment. The pairing is anonymous, meaning that neither individual will ever know the identity of the other individual with whom he or she is paired.

In this hypothetical experiment, Individual A will make a choice, the researcher will record this choice, and then both individuals will be informed of the choice and paid money based on the choice made by Individual A, as well as a small participation fee. Suppose that neither individual will receive any other money for participating in the experiment.

In each pair, Individual A will receive £10.  Individual A will then have the opportunity to give any amount of his or her £10 to Individual B. That is, Individual A can give any of the £10 he or she receives to Individual B. For instance, Individual A may decide to give £0 to Individual B and keep £10 for him or herself. Or Individual A may decide to give £10 to Individual B and keep £0 for him or herself. Individual A may also choose to give any other amount between £0 and £10 to Individual B. This choice will determine how much money each will receive, privately and in cash, at the end of the experiment.

The table below gives a list of the possible choices available to Individual A. For each of the choices, please indicate whether you believe choosing that option is extremely socially inappropriate, very socially inappropriate, somewhat socially inappropriate, somewhat socially appropriate, very socially appropriate, or extremely socially appropriate. To indicate your response, please select the corresponding option.

**Remember that you will earn money (£10) if your response to a randomly selected question is the same as the most common response provided in your school year group today.**

**Individual A's choice...**

|  | Extremely socially inappropriate | Very socially inappropriate | Somewhat socially inappropriate | Somewhat socially appropriate | Very socially appropriate | Extremely socially appropriate |
| --- | --- | --- | --- | --- | --- | --- |
| Give £0 to Individual B (Individual A gets £10, Individual B gets £0) |  |  |  |  |  |  |
| Give £1 to Individual B (Individual A gets £9, Individual B gets £1) |  |  |  |  |  |  |
| Give £2 to Individual B (Individual A gets £8, Individual B gets £2) |  |  |  |  |  |  |
| Give £3 to Individual B (Individual A gets £7, Individual B gets £3) |  |  |  |  |  |  |
| Give £4 to Individual B (Individual A gets £6, Individual B gets £4) |  |  |  |  |  |  |
| Give £5 to Individual B (Individual A gets £5, Individual B gets £5) |  |  |  |  |  |  |
| Give £6 to Individual B (Individual A gets £4, Individual B gets £6) |  |  |  |  |  |  |
| Give £7 to Individual B (Individual A gets £3, Individual B gets £7) |  |  |  |  |  |  |
| Give £8 to Individual B (Individual A gets £2, Individual B gets £8) |  |  |  |  |  |  |
| Give £9 to Individual B (Individual A gets £1, Individual B gets £9) |  |  |  |  |  |  |
| Give £10 to Individual B (Individual A gets £0, Individual B gets £10) |  |  |  |  |  |  |

**If you have any questions, please raise your hand and wait for the experimenter.**

**Part 2**

**Situation 2**

**A parent is smoking in their own home in front of their children who are under the age of 5.**

Please indicate whether you believe the parent smoking at home in front of their young children is extremely socially inappropriate, very socially inappropriate, somewhat socially inappropriate, somewhat socially appropriate, very socially appropriate, or extremely socially appropriate. To indicate your response, please select the corresponding option.

**Remember that you will earn money (£10) if your response to a randomly selected question is the same as the most common response provided in your school year group today.**

**A parent smoking at home in front of their young children.**

- Extremely socially inappropriate
- Very socially inappropriate
- Somewhat socially inappropriate
- Somewhat socially appropriate
- Very socially appropriate
- Extremely socially appropriate

**Part 2**

**Situation 3**

**An adult is smoking in a car with children under the age of 16 in the car.**

Please indicate whether you believe the adult smoking in the car with children in the car is extremely socially inappropriate, very socially inappropriate, somewhat socially inappropriate, somewhat socially appropriate, very socially appropriate, or extremely socially appropriate. To indicate your response, please select the corresponding option.

**Remember that you will earn money (£10) if your response to a randomly selected question is the same as the most common response provided in your school year group today.**

**Adult smoking in a car with children on board.**

- Extremely socially inappropriate
- Very socially inappropriate
- Somewhat socially inappropriate
- Somewhat socially appropriate
- Very socially appropriate
- Extremely socially appropriate

**Part 2**

**Situation 4**

**Someone sells cigarettes to a teenager who looks younger than 16 without requesting proof of age.**

Please indicate whether you believe someone selling cigarettes without proof of age is extremely socially inappropriate, very socially inappropriate, somewhat socially inappropriate, somewhat socially appropriate, very socially appropriate, or extremely socially appropriate. To indicate your response, please select the corresponding option.

**Remember that you will earn money (£10) if your response to a randomly selected question is the same as the most common response provided in your school year group today.**

**Someone selling cigarettes without proof of age.**

- Extremely socially inappropriate
- Very socially inappropriate
- Somewhat socially inappropriate
- Somewhat socially appropriate
- Very socially appropriate
- Extremely socially appropriate

**Part 2**

**Situation 5**

**In a recent superhero movie the lead actor is seen smoking in the opening scene.**

Please indicate whether you believe the movie showing the lead character smoking is extremely socially inappropriate, very socially inappropriate, somewhat socially inappropriate, somewhat socially appropriate, very socially appropriate, or extremely socially appropriate. To indicate your response, please select the corresponding option.

**Remember that you will earn money (£10) if your response to a randomly selected question is the same as the most common response provided in your school year group today.**

**A movie showing the lead character smoking.**

- Extremely socially inappropriate
- Very socially inappropriate
- Somewhat socially inappropriate
- Somewhat socially appropriate
- Very socially appropriate
- Extremely socially appropriate

**Part 2**

**Situation 6**

**An older student in your school is smoking outside school, for example, at a bus stop.**

Please indicate whether you believe an older student smoking outside school is extremely socially inappropriate, very socially inappropriate, somewhat socially inappropriate, somewhat socially appropriate, very socially appropriate, or extremely socially appropriate. To indicate your response, please select the corresponding option.

**Remember that you will earn money (£10) if your response to a randomly selected question is the same as the most common response provided in your school year group today.**

**An older student from your school smoking outside school.**

- Extremely socially inappropriate
- Very socially inappropriate
- Somewhat socially inappropriate
- Somewhat socially appropriate
- Very socially appropriate
- Extremely socially appropriate

**Part 2**

**Situation 7**

**A pupil from your school is using an e-cigarette while walking to school.**

Please indicate whether you believe the pupil using an e-cigarette is extremely socially inappropriate, very socially inappropriate, somewhat socially inappropriate, somewhat socially appropriate, very socially appropriate, or extremely socially appropriate. To indicate your response, please select the corresponding option.

**Remember that you will earn money (£10) if your response to a randomly selected question is the same as the most common response provided in your school year group today.**

**A school student smoking an e-cigarette.**

- Extremely socially inappropriate
- Very socially inappropriate
- Somewhat socially inappropriate
- Somewhat socially appropriate
- Very socially appropriate
- Extremely socially appropriate

**Part 2**

**Situation 8**

**A pupil from your school shares a photograph of him/herself using an e-cigarette on social media (e.g. Facebook, Instagram).**

Please indicate whether you believe the pupil sharing an image of e-cigarette use is extremely socially inappropriate, very socially inappropriate, somewhat socially inappropriate, somewhat socially appropriate, very socially appropriate, or extremely socially appropriate. To indicate your response, please select the corresponding option.

**Remember that you will earn money (£10) if your response to a randomly selected question is the same as the most common response provided in your school year group today.**

**A student sharing a photo of his/her e-cigarette use.**

- Extremely socially inappropriate
- Very socially inappropriate
- Somewhat socially inappropriate
- Somewhat socially appropriate
- Very socially appropriate
- Extremely socially appropriate

**Part 2**

**Situation 9**

**A pupil from your school is chewing tobacco.**

Please indicate whether you believe the pupil chewing tobacco is extremely socially inappropriate, very socially inappropriate, somewhat socially inappropriate, somewhat socially appropriate, very socially appropriate, or extremely socially appropriate. To indicate your response, please select the corresponding option.

**Remember that you will earn money (£10) if your response to a randomly selected question is the same as the most common response provided in your school year group today.**

**A school pupil chewing tobacco.**

- Extremely socially inappropriate
- Very socially inappropriate
- Somewhat socially inappropriate
- Somewhat socially appropriate
- Very socially appropriate
- Extremely socially appropriate

**This is the end of Part 2 of the experiment.**

In Part 3 of today's experiment you will be asked some questions about the behaviour of your peers. By peers, we mean “other students in your school year group”. After today we will randomly select a question from part 3. If you give the same response as that most frequently given by other people in your school year group, then your earning from Part 3 will be £10. This amount will be paid to you, in cash, at the conclusion of the study in ten weeks. Please click on the next button when you are ready to proceed.

**Part 3**

**Question 1**

**What share of your school year group would be accepting of one of their close friends smoking?**

*Please indicate what proportion of students in your school year group (your peers) you believe would be accepting of one of their close friends smoking: All of my peers; most of my peers; a lot of my peers; some of my peers; only a few of my peers; none of my peers. To indicate your response, please select the corresponding option.*

**Remember that you will earn money (£10) if your response to a randomly selected question is the same as the most common response provided in your school year group today.**

**The proportion of my peers who would be accepting of a close friend smoking.**

- All of my peers
- Most of my peers
- A lot of my peers
- Some of my peers
- Only a few of my peers
- None of my peers

**Part 3**

**Question 2**

**What share of your school year group would be accepting of one of their close friends vaping (using an e-cigarette)?**

Please indicate what proportion of students *in your school year group (your peers)* you believe would be accepting of one of their close friends using an e-cigarette: All of my peers; most of my peers; a lot of my peers; some of my peers; only a few of my peers; none of my peers. To indicate your response, please select the corresponding option.

**Remember that you will earn money (£10) if your response to a randomly selected question is the same as the most common response provided in your school year group today.**

**The proportion of my peers who would be accepting of a close friend vaping.**

- All of my peers
- Most of my peers
- A lot of my peers
- Some of my peers
- Only a few of my peers
- None of my peers

**This is the end of Part 3 of the experiment.**

Please wait for the experimenter to tell you when to proceed to Part 4.

**Part 4**

You will be given 10 virtual tokens. Each token is worth 50 pence. That means you will receive tokens worth £5.

You will then have the opportunity to give any amount of your £5 to the ASSIST Programme.

ASSIST is a smoking prevention programme which aims to prevent the uptake of smoking among adolescents your age.

You can give any of the £5 you receive to ASSIST. For instance, you may decide to give £0 to ASSIST and keep £5 for yourself. Or you may decide to give £5 to ASSIST and keep £0 for yourself. You may also choose to give any other amount between £0 and £5 to ASSIST.

The value of any tokens you do not give to ASSIST will be your earnings for this Part. That is, each token that you do not give to ASSIST will increase your own payment for Part 4 by 50 pence.

**How many tokens do you want to give to ASSIST?**

- 0 (you earn £5.00)
- 1 (you earn £4.50)
- 2 (you earn £4.00)
- 3 (you earn £3.50)
- 4 (you earn £3.00)
- 5 (you earn £2.50)
- 6 (you earn £2.00)
- 7 (you earn £1.50)
- 8 (you earn £1.00)
- 9 (you earn £0.50)
- 10 (you earn £0.00)

**
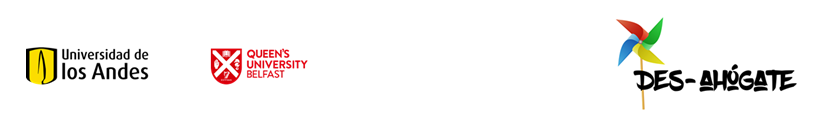
**

**Instrucciones para los experimentos**

**Información general**

Este es un estudio sobre toma de decisiones. Por participar te daremos un monto de $ 5.000 en una tarjeta de regalo. Además, puedes recibir un dinero extra en esta tarjeta en función de tus elecciones y las decisiones tomadas por otros durante el estudio.

Si tienes alguna pregunta durante la sesión, levanta la mano y espera a la persona encargada. Es muy importante que no hables ni trates de comunicarte con otros estudiantes durante el experimento. También es importante que todos los participantes tomen sus propias decisiones.

Este es un estudio que recibe financiación del Consejo de Investigación Médica del Reino Unido y cubrirá todos los gastos actuales y futuros. Por eso, puedes estar seguro de que a todos los estudiantes que participan se les pagará como se describe en las instrucciones en una tarjeta de regalo. Si tienes alguna duda, comunícate con el encargado en el salón o con Sharon Sánchez en la Facultad de Medicina de la Universidad de los Andes al teléfono 3394949 ext.3803 o al correo sc.sanchez@uniandes.edu.co.

**Hay cuatro partes en el estudio de hoy. Puedes ganar dinero en cada parte.**

Tus ganancias NO se pagarán hoy. La tarjeta de regalo será entregada al final del programa en 16 semanas. En ese momento volveremos para realizar otro estudio similar a este en el que podrás ganar dinero en cada parte. Tus ganancias dependen de tus respuestas en la sesión de experimentos de hoy y en la sesión del final del programa. Ten en cuenta que recibirás ganancias solo por una de las sesiones de experimentos, que se elegirá al azar lanzando una moneda. Para poder determinar tus ganancias, registraremos tus respuestas tanto en la sesión de hoy como en la sesión al final del programa. Si lo deseas, al final podrás verificar que el pago asignado corresponde a las respuestas que realizaste durante las dos sesiones.

**Parte 1**

En la parte 1 de este estudio, decidirás cómo encestar 50 pelotas entre dos baldes. Tu tarea es colocar cada una de las pelotas, una a una, en uno de los dos baldes: el balde azul o el balde amarillo. Las pelotas aparecerán en la parte izquierda de la pantalla, y puedes encestar cada pelota haciendo clic y arrastrándola al balde de tu elección. Por cada pelota que pongas en el balde azul, recibirás $100, y por cada pelota que pongas en el balde amarillo, recibirás $200.

La regla es poner las pelotas en el balde azul.

Una vez que comience el experimento, tendrás 5 minutos para poner las pelotas en los baldes. Cuando hayas terminado, espera en silencio hasta que el tiempo se acabe. Las bolas que no hayan sido encestadas en ningún balde no valen nada. Tus ganancias de la parte 1 se basarán en tus decisiones: es la suma de los pagos correspondientes a las pelotas que se encuentren en los baldes azules y amarillos.

Este es el final de las instrucciones para la parte 1. Si tienes alguna pregunta, levanta la mano y el encargado las contestará en privado. De lo contrario, espera en silencio hasta que todo el mundo haya terminado. **Solo cuando el encargado lo indique**, da clic en la siguiente pestaña para comenzar el experimento.

**Parte 1**

Tienes 50 pelotas. Por cada pelota que pongas en el balde azul, recibirás $100, y por cada bola que pongas en el balde amarillo, recibirás $200.


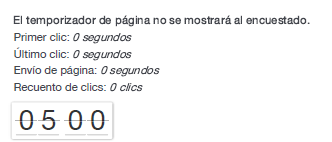


La regla es poner las bolas en el balde azul.

Arrastra y suelta cada pelota dentro del espacio correspondiente. Clasifique los elementos arrastrándolos y colocándolos en su lugar.


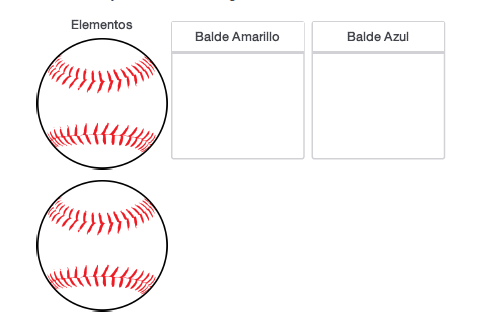


**Este es el final de la Parte 1 del experimento.**

Por favor, espera a que el encargado te indique cuándo empezar con la parte 2.

**Parte 2**

En las siguientes pantallas, se describirán varias situaciones. En estas situaciones una persona debe tomar una decisión o ha realizado una acción. Para cada situación, se describe la decisión o acción tomada por esta persona.

Después de leer la situación, se te pedirá que evalúes la decisión o acción tomada por esa persona. Debes decidir si lo que la persona hace sería “socialmente apropiado” y “consistente con un comportamiento moralmente aceptable o socialmente apropiado” o si el comportamiento de la persona sería “socialmente inapropiado” e “incompatible con una conducta moralmente aceptable o socialmente apropiada”. Por socialmente apropiado, nos referimos al comportamiento que la mayoría de las personas en tu grado escolar está de acuerdo que es lo “correcto” o “ético”. Otra forma de pensar sobre lo que queremos decir es que, si la persona en la situación descrita elije una opción socialmente inapropiada, alguien más en el grado escolar podría estar enojado con esa persona por hacerlo.

En cada una de tus respuestas, nos gustaría que respondas lo más sinceramente posible, en función de tus opiniones sobre lo que es un comportamiento socialmente apropiado o socialmente inapropiado.

Para explicarte cómo procederá el experimento, veremos un ejemplo y te mostraremos cómo podrías indicar tus respuestas. Recuerda que este es solo un ejemplo, tú podrás marcar cada respuesta de acuerdo con lo que opines. No hay respuestas buenas y malas. En la siguiente pantalla, verás un ejemplo de una situación y el encargado del salón lo explicará para todos.

**Parte 2**

**Ejemplo de situación.**

Una persona está en una cafetería cerca del colegio. Mientras está allí, la persona se da cuenta de que alguien ha dejado una billetera en una de las mesas. La persona debe decidir qué hacer. Esta persona tiene cuatro posibles opciones: llevarse la billetera, preguntar a otras personas que estén cerca si la billetera es suya, dejar la billetera donde está o darle la billetera al administrador de la tienda. La persona debe elegir una de las cuatro opciones.

*La siguiente tabla presenta una lista de las posibles opciones disponibles para esta persona. Para cada una de las opciones, debes indicar si crees que elegir esa opción es extremadamente inapropiado, socialmente muy inapropiado, socialmente algo inapropiado, socialmente algo apropiado, socialmente muy apropiado, extremadamente apropiado. Para indicar tu respuesta, marca la casilla correspondiente a la opción deseada:*

| **La persona elige:** | *Extremadamente inapropiado* | *Socialmente muy inapropiado* | *Socialmente algo inapropiado* | *Socialmente algo apropiado* | *Socialmente muy apropiado* | *Extremadamente apropiado* |
| --- | --- | --- | --- | --- | --- | --- |
| Llevarse la billetera | 🞆 | 🞆 | 🞆 | 🞆 | 🞆 | 🞆 |
| Preguntar a personas cercanas si la billetera es suya. | 🞆 | 🞆 | 🞆 | 🞆 | 🞆 | 🞆 |
| Dejar la billetera donde está | 🞆 | 🞆 | 🞆 | 🞆 | 🞆 | 🞆 |
| Darle la billetera al administrador de la tienda. | 🞆 | 🞆 | 🞆 | 🞆 | 🞆 | 🞆 |

**Por favor, asegúrate de seleccionar la opción deseada**

Si esta fuera una de las situaciones para este estudio, tú considerarías cada una de las posibles opciones anteriores y, para esa elección, indicarías hasta qué punto crees que tomar esa acción sería “socialmente apropiado” y “consistente con un comportamiento moralmente aceptable o socialmente apropiado” o si el comportamiento de la persona sería “socialmente inapropiado” e “incompatible con una conducta moralmente aceptable o socialmente apropiada. Recuerda que por “socialmente apropiado” nos referimos al comportamiento con el que la mayoría de las personas está de acuerdo, es lo “correcto” o “ético”.

**Parte 2**

Por ejemplo, supongamos que pensaste que tomar la billetera era *Extremadamente inapropiado*, preguntar a otras personas cercanas si la billetera era suya era *socialmente algo apropiado*, dejar la billetera donde estaba era *socialmente algo inapropiado* y darle la billetera al administrador de la tienda era *Extremadamente apropiado*. Entonces, tu habrías indicado tus respuestas de la siguiente manera:


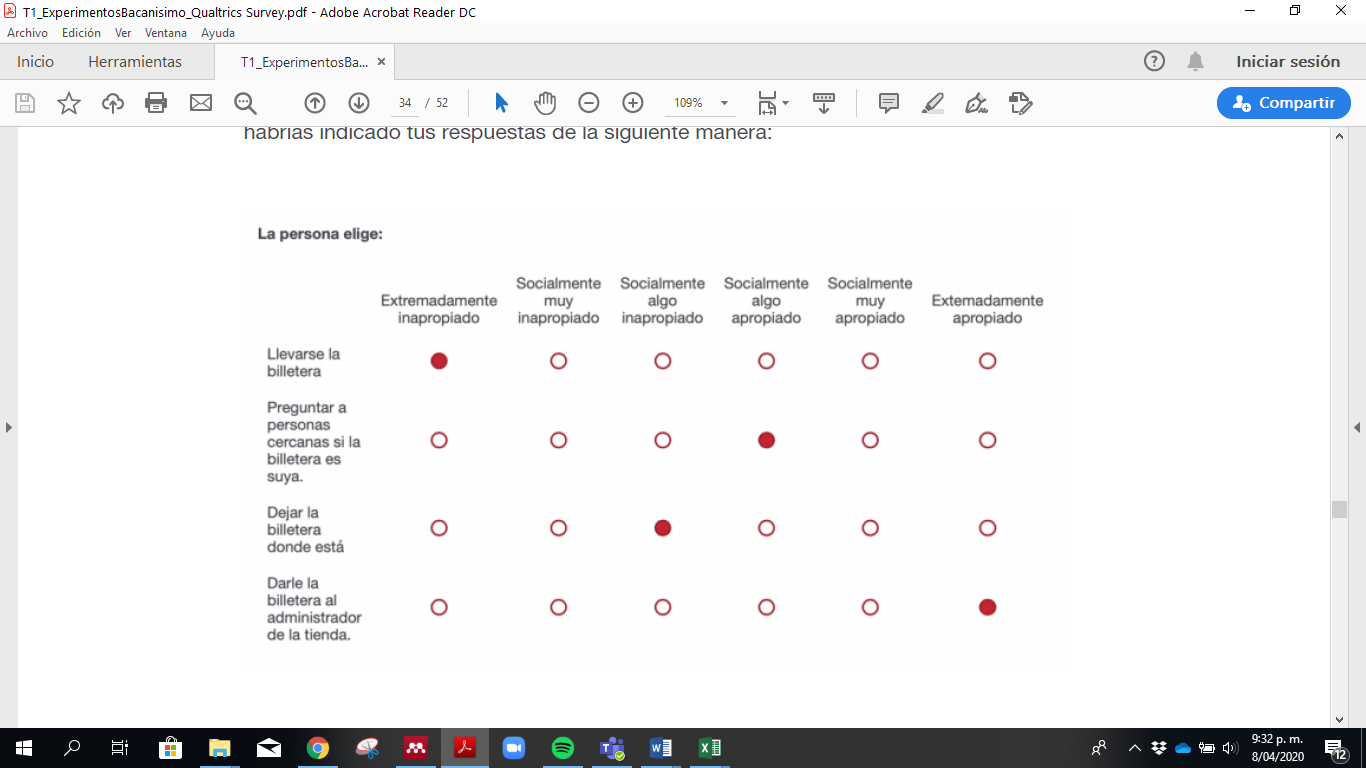


¿Hay alguna pregunta sobre este ejemplo o sobre cómo indicar tus respuestas? En las siguientes páginas, hay varias situaciones, todas relacionadas con decisiones que una persona podría tener que hacer.

Para cada situación, podrás marcar qué tan idóneo te parece cada acción en el recuadro correspondiente.

Al final del experimento de hoy, con un juego de lotería seleccionaremos al azar una de las situaciones. Para esta situación, también seleccionaremos al azar una de las opciones posibles que la persona podría hacer. Por lo tanto, seleccionaremos una situación y una posible elección al azar. Para la opción seleccionada, descubriremos qué respuesta fue seleccionada hoy por la mayoría de tus compañeros en el grado escolar.

Si tú escribes la misma respuesta que la mayoría de tus compañeros de grado, podrás recibir $15.000 por la parte 2 del experimento. Este premio se te entregará en la tarjeta de regalo al final del programa 16 semanas para EntreParceros. Por ejemplo, si en el ejemplo anterior, seleccionáramos al azar para premiar la opción “deje la billetera donde está”, y si tu respuesta fuese la misma que la mayoría de tus compañeros, entonces tu ganancia sería de $15.000, si esta fue la respuesta seleccionada por la mayoría de tus compañeros de grado el día de hoy. De lo contrario, su ganancia de la parte 2 sería $0.

Ahora, vas a completar algunas preguntas similares a este ejemplo por tu cuenta. Puedes ir a tu propio ritmo.

**Si tienes alguna pregunta a partir de este punto, levanta la mano y espera a que el encargado acuda.**

**Parte 2**

**Situación 1.**

Imagina dos personas hipotéticas de tu grado escolar que son ubicados en parejas al azar: Individuo A e Individuo B. El emparejamiento es anónimo, es decir, nadie sabe quién es la pareja de quién.

En este experimento hipotético, el individuo A hará una elección sobre dar dinero al otro, el encargado del curso registrará esta elección, y luego se la informará a ambas personas. Supongamos que ninguna persona recibirá ningún otro dinero diferente a lo que haga en esta situación hipotética.

En la pareja, el individuo A recibe $10.000. El individuo A tendrá entonces la oportunidad de dar cualquier cantidad de sus $10.000 al individuo B. Es decir, el individuo A puede darle al individuo B lo que quiera de los $10.000 que recibió. Por ejemplo, el individuo A puede decidir darle al individuo B $0 y mantener $10.000 para él o ella. O el individuo A puede decidir darle al individuo B los $10.000 y mantener $0 para él o ella. El individuo A también puede optar por dar cualquier otra cantidad entre $0 y $10.000 al individuo B.

Recuerda que tu respuesta sobre lo socialmente apropiado de las acciones del individuo A determinará la ganancia en tu tarjeta de regalo al final del estudio.

*La siguiente tabla presenta una lista de las posibles opciones disponibles para esta persona. Para cada una de las opciones, por favor, indica si crees que elegir esa opción es extremadamente inapropiado, socialmente muy inapropiado, socialmente algo inapropiado, socialmente algo apropiado, socialmente muy apropiado, extremadamente apropiado. Para indicar tu respuesta, marca la casilla correspondiente.*

Recuerda que ganarás un premio ($15.000) en la tarjeta de regalo si tu respuesta a una pregunta seleccionada al azar coincide con la respuesta más común brindada hoy por tus compañeros del grado escolar. Es decir, **para que sea más probable ganar el premio, debes responder según lo que tú crees que la mayoría de tus compañeros piensan.**

El individuo A elige:

| El individuo A elige: | *Extremadamente inapropiado* | *Socialmente muy inapropiado* | *Socialmente algo inapropiado* | *Socialmente algo apropiado* | *Socialmente muy apropiado* | *Extremadamente apropiado* |
| --- | --- | --- | --- | --- | --- | --- |
| Darle $0 al individuo B  (Individuo A obtiene $10.000, Individuo B obtiene $0) | 🞆 | 🞆 | 🞆 | 🞆 | 🞆 | 🞆 |
| Darle $1.000 al individuo B  (Individuo A obtiene $9.000, Individuo B obtiene $1.000 | 🞆 | 🞆 | 🞆 | 🞆 | 🞆 | 🞆 |
| Darle $2.000 al individuo B  (Individuo A obtiene $8.000, Individuo B obtiene $2.000) | 🞆 | 🞆 | 🞆 | 🞆 | 🞆 | 🞆 |
| Darle $3.000 al individuo B  (Individuo A obtiene $7.000, Individuo B obtiene $3.000) | 🞆 | 🞆 | 🞆 | 🞆 | 🞆 | 🞆 |
| Darle $4.000 al individuo B  (Individuo A obtiene $6.000, Individuo B obtiene $4.000) | 🞆 | 🞆 | 🞆 | 🞆 | 🞆 | 🞆 |
| Darle $5.000 al individuo B  (Individuo A obtiene $5.000, Individuo B obtiene $5.000) | 🞆 | 🞆 | 🞆 | 🞆 | 🞆 | 🞆 |
| Darle $6.000 al individuo B  (Individuo A obtiene $4.000, Individuo B obtiene $6.000) | 🞆 | 🞆 | 🞆 | 🞆 | 🞆 | 🞆 |
| Darle $5.000 al individuo B  (Individuo A obtiene $3.000, Individuo B obtiene $7.000) | 🞆 | 🞆 | 🞆 | 🞆 | 🞆 | 🞆 |
| Darle $8.000 al individuo B  (Individuo A obtiene $2.000, Individuo B obtiene $8.000) | 🞆 | 🞆 | 🞆 | 🞆 | 🞆 | 🞆 |
| Darle $9.000 al individuo B  (Individuo A obtiene $1.000, Individuo B obtiene $9.000) | 🞆 | 🞆 | 🞆 | 🞆 | 🞆 | 🞆 |
| Darle $10.000 al individuo B  (Individuo A obtiene $0, Individuo B obtiene $10.000) | 🞆 | 🞆 | 🞆 | 🞆 | 🞆 | 🞆 |

**Si tienes alguna pregunta, por favor levanta la mano y espera por el encargado del salón.**

**Parte 2**

**Situación 2.**

Un padre o una madre fuma en su propia casa frente a sus hijos menores de 5 años.

*Indica si crees que el padre fumando en la casa delante de sus hijos pequeños es extremadamente inapropiado, socialmente muy inapropiado, socialmente algo inapropiado, socialmente algo apropiado, socialmente muy apropiado, extremadamente apropiado. Para indicar tu respuesta marca la casilla correspondiente.*

Recuerda que ganarás un premio ($15.000) en la tarjeta de regalo si tu respuesta a una pregunta seleccionada al azar coincide con la respuesta más común brindada hoy por tus compañeros del grado escolar. Es decir, **para que sea más probable ganar el premio, debes responder según lo que tú crees que la mayoría de tus compañeros piensan.**

Un padre o una madre fuma en su propia casa frente a sus hijos menores de 5 años.

| *Extremadamente inapropiado* | *Socialmente muy inapropiado* | *Socialmente algo inapropiado* | *Socialmente algo apropiado* | *Socialmente muy apropiado* | *Extremadamente apropiado* |
| --- | --- | --- | --- | --- | --- |
| 🞆 | 🞆 | 🞆 | 🞆 | 🞆 | 🞆 |

**Parte 2**

**Situación 3.**

Un adulto fuma en un carro con personas menores de 16 años en el auto.

*Indica si crees que un adulto fumando en un carro con niños menores de 16 años en el carro es extremadamente inapropiado, socialmente muy inapropiado, socialmente algo inapropiado, socialmente algo apropiado, socialmente muy apropiado, extremadamente apropiado. Para indicar tu respuesta marca la casilla correspondiente.*

Recuerda que ganarás un premio ($15.000) en la tarjeta de regalo si tu respuesta a una pregunta seleccionada al azar coincide con la respuesta más común brindada hoy por tus compañeros del grado escolar. Es decir, **para que sea más probable ganar el premio, debes responder según lo que tú crees que la mayoría de tus compañeros piensan.**

Un adulto fuma en un carro con personas menores de 16 años en el auto.

| *Extremadamente inapropiado* | *Socialmente muy inapropiado* | *Socialmente algo inapropiado* | *Socialmente algo apropiado* | *Socialmente muy apropiado* | *Extremadamente apropiado* |
| --- | --- | --- | --- | --- | --- |
| 🞆 | 🞆 | 🞆 | 🞆 | 🞆 | 🞆 |

**Parte 2**

**Situación 4.**

Alguien vende cigarrillos a un adolescente que parece tener menos de 16 años sin pedirle la cédula para comprobar su edad.

*Indica si crees que alguien venda cigarrillos a un adolescente que parece tener menos de 16 años sin solicitar prueba de edad es extremadamente inapropiado, socialmente muy inapropiado, socialmente algo inapropiado, socialmente algo apropiado, socialmente muy apropiado, extremadamente apropiado.*

Recuerda que ganarás un premio ($15.000) en la tarjeta de regalo si tu respuesta a una pregunta seleccionada al azar coincide con la respuesta más común brindada hoy por tus compañeros del grado escolar. Es decir, **para que sea más probable ganar el premio, debes responder según lo que tú crees que la mayoría de tus compañeros piensan.**

Alguien vende cigarrillos a un adolescente que parece tener menos de 16 años sin pedirle la cédula para comprobar su edad.

| *Extremadamente inapropiado* | *Socialmente muy inapropiado* | *Socialmente algo inapropiado* | *Socialmente algo apropiado* | *Socialmente muy apropiado* | *Extremadamente apropiado* |
| --- | --- | --- | --- | --- | --- |
| 🞆 | 🞆 | 🞆 | 🞆 | 🞆 | 🞆 |

**Parte 2**

**Situación 5.**

En una película reciente de superhéroes, se ve al actor principal fumando en la primera escena.

*Indica si crees que en una película mostrando al actor principal fumando en la primera escena* *es extremadamente inapropiado, socialmente muy inapropiado, socialmente algo inapropiado, socialmente algo apropiado, socialmente muy apropiado, extremadamente apropiado.*

Recuerda que ganarás un premio ($15.000) en la tarjeta de regalo si tu respuesta a una pregunta seleccionada al azar coincide con la respuesta más común brindada hoy por tus compañeros del grado escolar. Es decir, **para que sea más probable ganar el premio, debes responder según lo que tú crees que la mayoría de tus compañeros piensan.**

En una película reciente de superhéroes, se ve al actor principal fumando en la primera escena.

| *Extremadamente inapropiado* | *Socialmente muy inapropiado* | *Socialmente algo inapropiado* | *Socialmente algo apropiado* | *Socialmente muy apropiado* | *Extremadamente apropiado* |
| --- | --- | --- | --- | --- | --- |
| 🞆 | 🞆 | 🞆 | 🞆 | 🞆 | 🞆 |

**Parte 2**

**Situación 6.**

Un estudiante mayor de tu colegio está fumando fuera del colegio, por ejemplo, en un paradero de bus.

*Indica si crees que un estudiante mayor de tu colegio está fumando fuera del colegio es extremadamente inapropiado, socialmente muy inapropiado, socialmente algo inapropiado, socialmente algo apropiado, socialmente muy apropiado, extremadamente apropiado. Para indicar tu respuesta marca la casilla correspondiente.*

Recuerda que ganarás un premio ($15.000) en la tarjeta de regalo si tu respuesta a una pregunta seleccionada al azar coincide con la respuesta más común brindada hoy por tus compañeros del grado escolar. Es decir, **para que sea más probable ganar el premio, debes responder según lo que tú crees que la mayoría de tus compañeros piensan.**

Un estudiante mayor de tu colegio está fumando fuera del colegio, por ejemplo, en un paradero de bus.

| *Extremadamente inapropiado* | *Socialmente muy inapropiado* | *Socialmente algo inapropiado* | *Socialmente algo apropiado* | *Socialmente muy apropiado* | *Extremadamente apropiado* |
| --- | --- | --- | --- | --- | --- |
| 🞆 | 🞆 | 🞆 | 🞆 | 🞆 | 🞆 |

**Parte 2**

**Situación 7.**

Un estudiante de tu colegio usa un cigarrillo electrónico mientras camina hacia el colegio.

*Indica si crees que el alumno usando un cigarrillo electrónico mientras camina hacia el colegio* *es extremadamente inapropiado, socialmente muy inapropiado, socialmente algo inapropiado, socialmente algo apropiado, socialmente muy apropiado, extremadamente apropiado. Para indicar tu respuesta marca la casilla correspondiente.*

Recuerda que ganarás un premio ($15.000) en la tarjeta de regalo si tu respuesta a una pregunta seleccionada al azar coincide con la respuesta más común brindada hoy por tus compañeros del grado escolar. Es decir, **para que sea más probable ganar el premio, debes responder según lo que tú crees que la mayoría de tus compañeros piensan.**

Un estudiante de tu colegio usa un cigarrillo electrónico mientras camina hacia el colegio.

| *Extremadamente inapropiado* | *Socialmente muy inapropiado* | *Socialmente algo inapropiado* | *Socialmente algo apropiado* | *Socialmente muy apropiado* | *Extremadamente apropiado* |
| --- | --- | --- | --- | --- | --- |
| 🞆 | 🞆 | 🞆 | 🞆 | 🞆 | 🞆 |

**Parte 2**

**Situación 8.**

Un estudiante de tu colegio comparte una fotografía de sí mismo utilizando un cigarrillo electrónico en redes sociales (Ej. Facebook o Instagram).

*Indica si crees que un alumno de tu colegio compartiendo una fotografía de sí mismo utilizando un cigarrillo electrónico en redes sociales (Ej. Facebook o Instagram*) *es extremadamente inapropiado, socialmente muy inapropiado, socialmente algo inapropiado, socialmente algo apropiado, socialmente muy apropiado, extremadamente apropiado.*

Recuerda que ganarás un premio ($15.000) en la tarjeta de regalo si tu respuesta a una pregunta seleccionada al azar coincide con la respuesta más común brindada hoy por tus compañeros del grado escolar. Es decir, **para que sea más probable ganar el premio, debes responder según lo que tú crees que la mayoría de tus compañeros piensan.**

Un estudiante de tu colegio comparte una fotografía de sí mismo utilizando un cigarrillo electrónico en redes sociales (Ej. Facebook o Instagram).

| *Extremadamente inapropiado* | *Socialmente muy inapropiado* | *Socialmente algo inapropiado* | *Socialmente algo apropiado* | *Socialmente muy apropiado* | *Extremadamente apropiado* |
| --- | --- | --- | --- | --- | --- |
| 🞆 | 🞆 | 🞆 | 🞆 | 🞆 | 🞆 |

**Parte 2**

**Situación 9.**

Un estudiante de tu colegio está masticando tabaco.

*Indica si crees que un estudiante de tu colegio masticando tabaco es extremadamente inapropiado, socialmente muy inapropiado, socialmente algo inapropiado, socialmente algo apropiado, socialmente muy apropiado, extremadamente apropiado. Para indicar tu respuesta marca la casilla correspondiente.*

Recuerda que ganarás un premio ($15.000) en la tarjeta de regalo si tu respuesta a una pregunta seleccionada al azar coincide con la respuesta más común brindada hoy por tus compañeros del grado escolar. Es decir, **para que sea más probable ganar el premio, debes responder según lo que tú crees que la mayoría de tus compañeros piensan.**

Un estudiante de tu colegio está masticando tabaco.

| *Extremadamente inapropiado* | *Socialmente muy inapropiado* | *Socialmente algo inapropiado* | *Socialmente algo apropiado* | *Socialmente muy apropiado* | *Extremadamente apropiado* |
| --- | --- | --- | --- | --- | --- |
| 🞆 | 🞆 | 🞆 | 🞆 | 🞆 | 🞆 |

**Este es el final de la Parte 2 del experimento.**

En la Parte 3 del experimento de hoy responderás varias preguntas sobre el comportamiento de tus compañeros, es decir, otros estudiantes en tu mismo grado escolar. Al final del estudio, con un juego de lotería seleccionaremos al azar una de las situaciones. Para esta situación, también seleccionaremos al azar una de las opciones posibles. Por lo tanto, seleccionaremos una situación y una posible elección al azar. Para la opción seleccionada, descubriremos qué respuesta fue seleccionada por la mayoría de tus compañeros en el grado escolar.

Si tú escribes la misma respuesta que la mayoría de tus compañeros de grado, podrás recibir $15.000 por la parte 3 del experimento. Este premio se te entregará en la tarjeta de regalo al final del programa 16 semanas para EntreParceros.

Por favor, da click en el botón cuando estés listo para empezar con la Parte 3.

**Parte 3**

**Pregunta 1.**

¿Cuántos de tus compañeros del grado aceptarían que uno de sus amigos cercanos fumara?

*Indica qué proporción de estudiantes del grado crees que aceptaría que uno de sus amigos cercanos fumara: todos mis compañeros, la mayoría de mis compañeros, muchos de mis compañeros, algunos de mis compañeros, solo unos pocos de mis compañeros, ninguno de mis compañeros. Para indicar tu respuesta marca la casilla correspondiente.*

Recuerda que ganarás un premio ($15.000) en la tarjeta de regalo si tu respuesta a una pregunta seleccionada al azar coincide con la respuesta más común brindada hoy por tus compañeros del grado escolar. Es decir, **para que sea más probable ganar el premio, debes responder según lo que tú crees que la mayoría de tus compañeros piensan.**

¿Cuántos de tus compañeros del grado aceptarían que uno de sus amigos cercanos fumara?

| *Todos mis compañeros* | *La mayoría de mis compañeros* | *Muchos de mis compañeros* | *Algunos de mis compañeros* | *Solo unos pocos de mis compañeros* | *Ninguno de mis compañeros* |
| --- | --- | --- | --- | --- | --- |
| 🞆 | 🞆 | 🞆 | 🞆 | 🞆 | 🞆 |

**Parte 3**

**Pregunta 2.**

¿Cuántos de tus compañeros del grado aceptarían que uno de tus amigos cercanos usara un cigarrillo electrónico?

*Indica qué proporción de estudiantes del grado crees que aceptaría que uno de sus amigos cercanos usara un cigarrillo electrónico: todos mis compañeros, la mayoría de mis compañeros, muchos de mis compañeros, algunos de mis compañeros, solo unos pocos de mis compañeros, ninguno de mis compañeros. Para indicar tu respuesta marca la casilla correspondiente.*

Recuerda que ganarás un premio ($15.000) en la tarjeta de regalo si tu respuesta a una pregunta seleccionada al azar coincide con la respuesta más común brindada hoy por tus compañeros del grado escolar. Es decir, **para que sea más probable ganar el premio, debes responder según lo que tú crees que la mayoría de tus compañeros piensan.**

¿Cuántos de tus compañeros del grado aceptarían que uno de tus amigos cercanos usara un cigarrillo electrónico?

| *Todos mis compañeros* | *La mayoría de mis compañeros* | *Muchos de mis compañeros* | *Algunos de mis compañeros* | *Solo unos pocos de mis compañeros* | *Ninguno de mis compañeros* |
| --- | --- | --- | --- | --- | --- |
| 🞆 | 🞆 | 🞆 | 🞆 | 🞆 | 🞆 |

**Este es el final de la Parte 3 del experimento**

Por favor, espera a que el encargado del salón te indique que puedes seguir con la Parte 4.

**Parte 4.**

Se te darán 10 fichas virtuales equivalentes a $10.000. Es decir, recibirás 10 fichas de $1.000 cada una. A continuación, tendrás la oportunidad de dar cualquier cantidad de tus $10.000 al programa Des-ahógate [reemplazar con el nombre del programa asignado a la institución Des-ahógate – EntreParceros. El programa de prevención Des-ahógate EntreParceros pretende evitar el consumo de tabaco entre adolescentes de tu edad.

Puedes dar cualquier cantidad de los $10.000 que recibiste a Des-ahógate - EntreParceros. Por ejemplo, puedes decidir dar $0 a Des-ahógate EntreParceros y mantener $10.000 para ti. O puedes decidir dar $10.000 a Des-ahógate EntreParceros y mantener $0 para ti. También puedes optar por dar cualquier otra cantidad entre $0 y $10.000 a Des-ahógate EntreParceros.

La cantidad de dinero que no le des a Des-ahógate EntreParceros hará parte de tus ganancias en esta parte. Recuerda que cada ficha equivale a $1.000.

¿Cuántas **fichas** quieres donar a Des-ahógate EntreParceros?

| 🞆 | 0 (tú ganas $10.000) |
| --- | --- |
| 🞆 | 1 (tú ganas $9.000) |
| 🞆 | 2 (tú ganas $8.000) |
| 🞆 | 3 (tú ganas $7.000) |
| 🞆 | 4 (tú ganas $6.000) |
| 🞆 | 5 (tú ganas $5.000) |
| 🞆 | 6 (tú ganas $4.000) |
| 🞆 | 7 (tú ganas $3.000) |
| 🞆 | 8 (tú ganas $2.000) |
| 🞆 | 9 (tú ganas $1.000) |
| 🞆 | 10 (tú ganas $0) |

Gracias por participar de este experimento.

Tus respuestas serán guardas para determinar tus ganancias.

Recuerda que en 16 semanas semanas volveremos para hacer otro estudio igual a este.

**Supplement 3: Syntax used to generate results**

**Confirmatory factor analysis models**

model1 <- 'ExptInj =~ p2sit2 + p2sit3 + p2sit4 + p2sit5 + p2sit6 + p2sit7 + p2sit8 + p2sit9

p2sit2 ~~ p2sit3

p2sit7 ~~ p2sit8

p2sit2 ~~ p2sit4'

fit1 <- cfa(model = model1, data = inData, estimator="MLR", missing="fiml")

summary(fit1,fit.measures=TRUE,standardized=TRUE)

fit1.2 <- cfa(model = model1, data = inData,std.lv = TRUE, estimator="MLR", missing="fiml")

summary(fit1.2,fit.measures=TRUE,standardized=TRUE,rsquare=TRUE)

modificationIndices(fit1.2)

model2 <- 'SurveyInj =~ injunc1 + injunc2 + injunc3 + injunc4 + injunc5 + injunc6 + injunc7

injunc2 ~~ injunc3

injunc4 ~~ injunc5

injunc6 ~~ injunc7'

fit2 <- cfa(model = model2, data = inData, estimator="MLR", missing="fiml")

summary(fit2,fit.measures=TRUE,standardized=TRUE)

fit2.2 <- cfa(model = model2, data = inData,std.lv = TRUE, estimator="MLR", missing="fiml")

summary(fit2.2,fit.measures=TRUE,standardized=TRUE,rsquare=TRUE)

modificationIndices(fit2.2)

model3 <- 'ExptDesc =~ a*p3q1 + a*p3q2'

fit3 <- cfa(model = model3, data = inData, estimator="MLR", missing="fiml")

summary(fit3,fit.measures=TRUE,standardized=TRUE)

fit3.2 <- cfa(model = model3, data = inData,std.lv = TRUE, estimator="MLR", missing="fiml")

summary(fit3.2,fit.measures=TRUE,standardized=TRUE,rsquare=TRUE)

modificationIndices(fit3.2)

model4 <- 'SurveyDesc =~ desc1 + desc2 + desc3 + desc4 + desc5

desc2 ~~ desc3

desc4 ~~ desc5'

fit4 <- cfa(model = model4, data = inData, estimator="MLR", missing="fiml")

summary(fit4,fit.measures=TRUE,standardized=TRUE)

fit4.2 <- cfa(model = model4, data = inData,std.lv = TRUE, estimator="MLR", missing="fiml")

summary(fit4.2,fit.measures=TRUE,standardized=TRUE,rsquare=TRUE)

modificationIndices(fit4.2)

model5 <- 'ExptInj =~ p2sit2 + p2sit3 + p2sit4 + p2sit5 + p2sit6 + p2sit7 + p2sit8 + p2sit9

SurveyInj =~ injunc1 + injunc2 + injunc3 + injunc4 + injunc5 + injunc6 + injunc7

ExptDesc =~ a*p3q1 + a*p3q2

SurveyDesc =~ desc1 + desc2 + desc3 + desc4 + desc5

p2sit2 ~~ p2sit3

p2sit7 ~~ p2sit8

p2sit2 ~~ p2sit4

injunc2 ~~ injunc3

injunc4 ~~ injunc5

injunc6 ~~ injunc7

desc2 ~~ desc3

desc4 ~~ desc5

ExptInj ~~ SurveyInj

ExptInj ~~ ExptDesc

ExptInj ~~ SurveyDesc

SurveyInj ~~ ExptDesc

SurveyInj ~~ SurveyDesc

ExptDesc ~~ SurveyDesc'

fit5 <- cfa(model = model5, data = inData, estimator="MLR", missing="fiml")

summary(fit5,fit.measures=TRUE,standardized=TRUE)

fit5.2 <- cfa(model = model5, data = inData,std.lv = TRUE, estimator="MLR", missing="fiml")

summary(fit5.2,fit.measures=TRUE,standardized=TRUE,rsquare=TRUE)

modificationIndices(fit5.2)

model6 <- 'ExptInj =~ p2sit2 + p2sit3 + p2sit4 + p2sit5 + p2sit6 + p2sit7 + p2sit8 + p2sit9

SurveyInj =~ injunc1 + injunc2 + injunc3 + injunc4 + injunc5 + injunc6 + injunc7

ExptDesc =~ a*p3q1 + a*p3q2

SurveyDesc =~ desc1 + desc2 + desc3 + desc4 + desc5

p2sit2 ~~ p2sit3

p2sit7 ~~ p2sit8

p2sit2 ~~ p2sit4

injunc2 ~~ injunc3

injunc4 ~~ injunc5

injunc6 ~~ injunc7

desc2 ~~ desc3

desc4 ~~ desc5

Norm =~ ExptInj + ExptDesc + SurveyInj + SurveyDesc'

fit6 <- cfa(model = model6, data = inData, estimator="MLR", missing="fiml")

summary(fit6,fit.measures=TRUE,standardized=TRUE)

fit6.2 <- cfa(model = model6, data = inData,std.lv = TRUE, estimator="MLR", missing="fiml")

summary(fit6.2,fit.measures=TRUE,standardized=TRUE,rsquare=TRUE)

modificationIndices(fit6.2)

**Multiple indicators multiple causes models**

model7 <- 'ExptInj =~ p2sit2 + p2sit3 + p2sit4 + p2sit5 + p2sit6 + p2sit7 + p2sit8 + p2sit9

SurveyInj =~ injunc1 + injunc2 + injunc3 + injunc4 + injunc5 + injunc6 + injunc7

ExptDesc =~ a*p3q1 + a*p3q2

SurveyDesc =~ desc1 + desc2 + desc3 + desc4 + desc5

p2sit2 ~~ p2sit3

p2sit7 ~~ p2sit8

p2sit2 ~~ p2sit4

injunc2 ~~ injunc3

injunc4 ~~ injunc5

injunc6 ~~ injunc7

desc2 ~~ desc3

desc4 ~~ desc5

Norm =~ ExptInj + ExptDesc + SurveyInj + SurveyDesc

Norm ~ Country'

fit7 <- cfa(model = model7, data = inData, estimator="MLR", missing="fiml")

summary(fit7,fit.measures=TRUE,standardized=TRUE)

fit7.2 <- cfa(model = model7, data = inData,std.lv = TRUE, estimator="MLR", missing="fiml")

summary(fit7.2,fit.measures=TRUE,standardized=TRUE,rsquare=TRUE)

model8 <- 'ExptInj =~ p2sit2 + p2sit3 + p2sit4 + p2sit5 + p2sit6 + p2sit7 + p2sit8 + p2sit9

SurveyInj =~ injunc1 + injunc2 + injunc3 + injunc4 + injunc5 + injunc6 + injunc7

ExptDesc =~ a*p3q1 + a*p3q2

SurveyDesc =~ desc1 + desc2 + desc3 + desc4 + desc5

p2sit2 ~~ p2sit3

p2sit7 ~~ p2sit8

p2sit2 ~~ p2sit4

injunc2 ~~ injunc3

injunc4 ~~ injunc5

injunc6 ~~ injunc7

desc2 ~~ desc3

desc4 ~~ desc5

Norm =~ ExptInj + ExptDesc + SurveyInj + SurveyDesc

ExptInj ~ Country

SurveyInj ~ Country

ExptDesc ~ Country

SurveyDesc ~ Country'

fit8 <- cfa(model = model8, data = inData, estimator="MLR", missing="fiml")

summary(fit8,fit.measures=TRUE,standardized=TRUE)

fit8.2 <- cfa(model = model8, data = inData,std.lv = TRUE, estimator="MLR", missing="fiml")

summary(fit8.2,fit.measures=TRUE,standardized=TRUE,rsquare=TRUE)

**Differential item functioning analyses**

model9 <- 'ExptInj =~ p2sit2 + p2sit3 + p2sit4 + p2sit5 + p2sit6 + p2sit7 + p2sit8 + p2sit9

SurveyInj =~ injunc1 + injunc2 + injunc3 + injunc4 + injunc5 + injunc6 + injunc7

ExptDesc =~ a*p3q1 + a*p3q2

SurveyDesc =~ desc1 + desc2 + desc3 + desc4 + desc5

p2sit2 ~~ p2sit3

p2sit7 ~~ p2sit8

p2sit2 ~~ p2sit4

injunc2 ~~ injunc3

injunc4 ~~ injunc5

injunc6 ~~ injunc7

desc2 ~~ desc3

desc4 ~~ desc5

Norm =~ ExptInj + ExptDesc + SurveyInj + SurveyDesc

ExptInj ~ Country

SurveyInj ~ Country

ExptDesc ~ Country

SurveyDesc ~ Country

p2sit2 ~ 0*Country

p2sit3 ~ 0*Country

p2sit4 ~ 0*Country

p2sit5 ~ 0*Country

p2sit6 ~ 0*Country

p2sit7 ~ 0*Country

p2sit8 ~ 0*Country

p2sit9 ~ 0*Country

injunc1 ~ 0*Country

injunc2 ~ 0*Country

injunc3 ~ 0*Country

injunc4 ~ 0*Country

injunc5 ~ 0*Country

injunc6 ~ 0*Country

injunc7 ~ 0*Country

p3q1 ~ 0*Country

p3q2 ~ 0*Country

desc1 ~ 0*Country

desc2 ~ 0*Country

desc3 ~ 0*Country

desc4 ~ 0*Country

desc5 ~ 0*Country'

fit9 <- cfa(model = model9, data = inData, estimator="MLR", missing="fiml")

summary(fit9,fit.measures=TRUE,standardized=TRUE)

fit9.2 <- cfa(model = model9, data = inData,std.lv = TRUE, estimator="MLR", missing="fiml")

summary(fit9.2,fit.measures=TRUE,standardized=TRUE,rsquare=TRUE)

modificationIndices(fit9.2, power=TRUE)

**Measurement models controlling for differential item functioning**

model10 <- 'ExptInj =~ p2sit2 + p2sit3 + p2sit4 + p2sit5 + p2sit6 + p2sit7 + p2sit8 + p2sit9

SurveyInj =~ injunc1 + injunc2 + injunc3 + injunc4 + injunc5 + injunc6 + injunc7

ExptDesc =~ a*p3q1 + a*p3q2

SurveyDesc =~ desc1 + desc2 + desc3 + desc4 + desc5

p2sit2 ~~ p2sit3

p2sit7 ~~ p2sit8

p2sit2 ~~ p2sit4

injunc2 ~~ injunc3

injunc4 ~~ injunc5

injunc6 ~~ injunc7

desc2 ~~ desc3

desc4 ~~ desc5

Norm =~ ExptInj + ExptDesc + SurveyInj + SurveyDesc

ExptInj ~ Country

SurveyInj ~ Country

ExptDesc ~ Country

SurveyDesc ~ Country

p2sit2 ~ Country

p2sit5 ~ Country

injunc1 ~ Country

injunc4 ~ Country

desc2 ~ Country

desc3 ~ Country'

fit10 <- cfa(model = model10, data = inData, estimator="MLR", missing="fiml")

summary(fit10,fit.measures=TRUE,standardized=TRUE)

fit10.2 <- cfa(model = model10, data = inData,std.lv = TRUE, estimator="MLR", missing="fiml")

summary(fit10.2,fit.measures=TRUE,standardized=TRUE,rsquare=TRUE)

**Structural equation models**

model11 <- 'ExptInj =~ p2sit2 + p2sit3 + p2sit4 + p2sit5 + p2sit6 + p2sit7 + p2sit8 + p2sit9

SurveyInj =~ injunc1 + injunc2 + injunc3 + injunc4 + injunc5 + injunc6 + injunc7

ExptDesc =~ a*p3q1 + a*p3q2

SurveyDesc =~ desc1 + desc2 + desc3 + desc4 + desc5

p2sit2 ~~ p2sit3

p2sit7 ~~ p2sit8

p2sit2 ~~ p2sit4

injunc2 ~~ injunc3

injunc4 ~~ injunc5

injunc6 ~~ injunc7

desc2 ~~ desc3

desc4 ~~ desc5

Norm =~ ExptInj + ExptDesc + SurveyInj + SurveyDesc

ExptInj ~ Country

SurveyInj ~ Country

ExptDesc ~ Country

SurveyDesc ~ Country

p2sit2 ~ Country

p2sit5 ~ Country

injunc1 ~ Country

injunc4 ~ Country

desc2 ~ Country

desc3 ~ Country

SmokePast ~ Norm + Country'

fit11 <- cfa(model = model11, data = inData, estimator="MLR", missing="fiml")

summary(fit11,fit.measures=TRUE,standardized=TRUE)

fit11.2 <- cfa(model = model11, data = inData,std.lv = TRUE, estimator="MLR", missing="fiml")

summary(fit11.2,fit.measures=TRUE,standardized=TRUE,rsquare=TRUE)

model12 <- 'ExptInj =~ p2sit2 + p2sit3 + p2sit4 + p2sit5 + p2sit6 + p2sit7 + p2sit8 + p2sit9

SurveyInj =~ injunc1 + injunc2 + injunc3 + injunc4 + injunc5 + injunc6 + injunc7

ExptDesc =~ a*p3q1 + a*p3q2

SurveyDesc =~ desc1 + desc2 + desc3 + desc4 + desc5

p2sit2 ~~ p2sit3

p2sit7 ~~ p2sit8

p2sit2 ~~ p2sit4

injunc2 ~~ injunc3

injunc4 ~~ injunc5

injunc6 ~~ injunc7

desc2 ~~ desc3

desc4 ~~ desc5

Norm =~ ExptInj + ExptDesc + SurveyInj + SurveyDesc

ExptInj ~ Country

SurveyInj ~ Country

ExptDesc ~ Country

SurveyDesc ~ Country

p2sit2 ~ Country

p2sit5 ~ Country

injunc1 ~ Country

injunc4 ~ Country

desc2 ~ Country

desc3 ~ Country

SmokePast ~ ExptInj + SurveyInj + ExptDesc + SurveyDesc + Country'

fit12 <- cfa(model = model12, data = inData, estimator="MLR", missing="fiml")

summary(fit12,fit.measures=TRUE,standardized=TRUE)

fit12.2 <- cfa(model = model12, data = inData,std.lv = TRUE, estimator="MLR", missing="fiml")

summary(fit12.2,fit.measures=TRUE,standardized=TRUE,rsquare=TRUE)

model13 <- 'ExptInj =~ p2sit2 + p2sit3 + p2sit4 + p2sit5 + p2sit6 + p2sit7 + p2sit8 + p2sit9

SurveyInj =~ injunc1 + injunc2 + injunc3 + injunc4 + injunc5 + injunc6 + injunc7

ExptDesc =~ a*p3q1 + a*p3q2

SurveyDesc =~ desc1 + desc2 + desc3 + desc4 + desc5

p2sit2 ~~ p2sit3

p2sit7 ~~ p2sit8

p2sit2 ~~ p2sit4

injunc2 ~~ injunc3

injunc4 ~~ injunc5

injunc6 ~~ injunc7

desc2 ~~ desc3

desc4 ~~ desc5

Norm =~ ExptInj + ExptDesc + SurveyInj + SurveyDesc

ExptInj ~ Country

SurveyInj ~ Country

ExptDesc ~ Country

SurveyDesc ~ Country

p2sit2 ~ Country

p2sit5 ~ Country

injunc1 ~ Country

injunc4 ~ Country

desc2 ~ Country

desc3 ~ Country

Intent ~ Norm + Country'

fit13 <- cfa(model = model13, data = inData, estimator="MLR", missing="fiml")

summary(fit13,fit.measures=TRUE,standardized=TRUE)

fit13.2 <- cfa(model = model13, data = inData,std.lv = TRUE, estimator="MLR", missing="fiml")

summary(fit13.2,fit.measures=TRUE,standardized=TRUE,rsquare=TRUE)

model14 <- 'ExptInj =~ p2sit2 + p2sit3 + p2sit4 + p2sit5 + p2sit6 + p2sit7 + p2sit8 + p2sit9

SurveyInj =~ injunc1 + injunc2 + injunc3 + injunc4 + injunc5 + injunc6 + injunc7

ExptDesc =~ a*p3q1 + a*p3q2

SurveyDesc =~ desc1 + desc2 + desc3 + desc4 + desc5

p2sit2 ~~ p2sit3

p2sit7 ~~ p2sit8

p2sit2 ~~ p2sit4

injunc2 ~~ injunc3

injunc4 ~~ injunc5

injunc6 ~~ injunc7

desc2 ~~ desc3

desc4 ~~ desc5

Norm =~ ExptInj + ExptDesc + SurveyInj + SurveyDesc

ExptInj ~ Country

SurveyInj ~ Country

ExptDesc ~ Country

SurveyDesc ~ Country

p2sit2 ~ Country

p2sit5 ~ Country

injunc1 ~ Country

injunc4 ~ Country

desc2 ~ Country

desc3 ~ Country

Intent ~ ExptInj + SurveyInj + ExptDesc + SurveyDesc + Country'

fit14 <- cfa(model = model14, data = inData, estimator="MLR", missing="fiml")

summary(fit14,fit.measures=TRUE,standardized=TRUE)

fit14.2 <- cfa(model = model14, data = inData,std.lv = TRUE, estimator="MLR", missing="fiml")

summary(fit14.2,fit.measures=TRUE,standardized=TRUE,rsquare=TRUE)

model15 <- 'ExptInj =~ p2sit2 + p2sit3 + p2sit4 + p2sit5 + p2sit6 + p2sit7 + p2sit8 + p2sit9

SurveyInj =~ injunc1 + injunc2 + injunc3 + injunc4 + injunc5 + injunc6 + injunc7

ExptDesc =~ a*p3q1 + a*p3q2

SurveyDesc =~ desc1 + desc2 + desc3 + desc4 + desc5

p2sit2 ~~ p2sit3

p2sit7 ~~ p2sit8

p2sit2 ~~ p2sit4

injunc2 ~~ injunc3

injunc4 ~~ injunc5

injunc6 ~~ injunc7

desc2 ~~ desc3

desc4 ~~ desc5

Norm =~ ExptInj + ExptDesc + SurveyInj + SurveyDesc

ExptInj ~ Country

SurveyInj ~ Country

ExptDesc ~ Country

SurveyDesc ~ Country

p2sit2 ~ Country

p2sit5 ~ Country

injunc1 ~ Country

injunc4 ~ Country

desc2 ~ Country

desc3 ~ Country

COreading ~ Norm + Country'

fit15 <- cfa(model = model15, data = inData, estimator="MLR", missing="fiml")

summary(fit15,fit.measures=TRUE,standardized=TRUE)

fit15.2 <- cfa(model = model15, data = inData,std.lv = TRUE, estimator="MLR", missing="fiml")

summary(fit15.2,fit.measures=TRUE,standardized=TRUE,rsquare=TRUE)

model16 <- 'ExptInj =~ p2sit2 + p2sit3 + p2sit4 + p2sit5 + p2sit6 + p2sit7 + p2sit8 + p2sit9

SurveyInj =~ injunc1 + injunc2 + injunc3 + injunc4 + injunc5 + injunc6 + injunc7

ExptDesc =~ a*p3q1 + a*p3q2

SurveyDesc =~ desc1 + desc2 + desc3 + desc4 + desc5

p2sit2 ~~ p2sit3

p2sit7 ~~ p2sit8

p2sit2 ~~ p2sit4

injunc2 ~~ injunc3

injunc4 ~~ injunc5

injunc6 ~~ injunc7

desc2 ~~ desc3

desc4 ~~ desc5

Norm =~ ExptInj + ExptDesc + SurveyInj + SurveyDesc

ExptInj ~ Country

SurveyInj ~ Country

ExptDesc ~ Country

SurveyDesc ~ Country

p2sit2 ~ Country

p2sit5 ~ Country

injunc1 ~ Country

injunc4 ~ Country

desc2 ~ Country

desc3 ~ Country

COreading ~ ExptInj + SurveyInj + ExptDesc + SurveyDesc + Country'

fit16 <- cfa(model = model16, data = inData, estimator="MLR", missing="fiml")

summary(fit16,fit.measures=TRUE,standardized=TRUE)

fit16.2 <- cfa(model = model16, data = inData,std.lv = TRUE, estimator="MLR", missing="fiml")

summary(fit16.2,fit.measures=TRUE,standardized=TRUE,rsquare=TRUE)

**Supplement 4: Summary statistics and histograms showing response distributions for all variables**

**Baseline and follow-up summary statistics.**

|  | **Northern Ireland (N=7)** | | **Colombia (N=8)** | | **All schools (N=15)** | |
| --- | --- | --- | --- | --- | --- | --- |
|  | **Baseline** | **Follow-up** | **Baseline** | **Follow-up** | **Baseline** | **Follow-up** |
| Experiment, n | 696 | 684 | 880 | 852 | 1576 | 1536 |
| Survey, n | 701 | 654 | 872 | 846 | 1573 | 1500 |
| **Experiment Part 1 (rule-following task)** | | | | | | |
| Blue bucket (1-50)^a^ | | | | | | |
| *Mean (SD)* | 28.8 (19.2) | 29.0 (20.3) | 31.6 (16.9) | 32.7 (17.7) | 30.4 (18.0) | 31.1 (19.0) |
| *Median (IQR)* | 26.0 (11.5 to 50.0) | 27.0 (2.0 to 50.0) | 30.0 (22.0 to 50.0) | 35.0 (23.0 to 50.0) | 28.0 (21.0 to 50.0) | 33.0 (19.5 to 50.0) |
| Yellow bucket (1-50)^a^ | | | | | | |
| *Mean (SD)* | 21.2 (19.2) | 21.0 (20.3) | 18.4 (16.9) | 17.3 (17.7) | 19.6 (18.0) | 18.9 (19.0) |
| *Median (IQR)* | 24.0 (0.0 to 38.5) | 23.0 (0.0 to 48.0) | 20.0 (0.0 to 28.0) | 15.0 (0.0 to 27.0) | 22.0 (0.0 to 29.0) | 17.0 (0.0 to 30.5) |
| **Experiment Part 2 (injunctive social norms)^b^** | | | | | | |
| Situation 2 (Parent smoking in front of young children) | | | | | | |
| *Mean (SD)* | -0.8 (0.3) | -0.8 (0.4) | -0.9 (0.2) | -0.9 (0.3) | -0.9 (0.3) | -0.9 (0.3) |
| *Median (IQR)* | -1.0 (-1.0 to -0.6) | -1.0 (-1.0 to -0.6) | -1.0 (-1.0 to -1.0) | -1.0 (-1.0 to -1.0) | -1.0 (-1.0 to -1.0) | -1.0 (-1.0 to -1.0) |
| *Modal response, n (%)* | 498 (71.7%) | 449 (65.7%) | 806 (91.6%) | 706 (82.9%) | 1304 (82.8%) | 1155 (75.2%) |
| Situation 3 (Adult smoking in car with under 16's onboard) | | | | | | |
| *Mean (SD)* | -0.7 (0.4) | -0.7 (0.4) | -0.7 (0.3) | -0.7 (0.3) | -0.7 (0.4) | -0.7 (0.3) |
| *Median (IQR)* | -0.6 (-1.0 to -0.6) | -0.6 (-1.0 to -0.6) | -0.8 (-1.0 to -0.6) | -0.6 (-1.0 to -0.6) | -0.6 (-1.0 to -0.6) | -0.6 (-1.0 to -0.6) |
| *Modal response, n (%)* | 316 (45.5%) | 314 (45.9%) | 444 (50.5%) | 439 (51.5%) | 760 (48.3%) | 753 (49.0%) |
| Situation 4 (Selling cigarettes to someone who looks younger than 16 without asking for proof of age) | | | | | | |
| *Mean (SD)* | -0.9 (0.3) | -0.8 (0.3) | -0.9 (0.3) | -0.8 (0.3) | -0.9 (0.3) | -0.8 (0.3) |
| *Median (IQR)* | -1.0 (-1.0 to -1.0) | -1.0 (-1.0 to -0.6) | -1.0 (-1.0 to -1.0) | -1.0 (-1.0 to -0.6) | -1.0 (-1.0 to -1.0) | -1.0 (-1.0 to -0.6) |
| *Modal response, n (%)* | 537 (77.2%) | 482 (70.8%) | 676 (76.8%) | 529 (62.1%) | 1213 (77.0%) | 1011 (65.9%) |
| Situation 5 (Lead actor smoking in opening scene of a recent superhero movie) | | | | | | |
| *Mean (SD)* | -0.3 (0.4) | -0.3 (0.4) | -0.5 (0.4) | -0.4 (0.4) | -0.4 (0.4) | -0.4 (0.4) |
| *Median (IQR)* | -0.2 (-0.6 to -0.2) | -0.2 (-0.6 to -0.2) | -0.6 (-1.0 to -0.2) | -0.2 (-1.0 to -0.2) | -0.2 (-0.6 to -0.2) | -0.2 (-0.6 to -0.2) |
| *Modal response, n (%)* | 295 (42.4%) | 334 (48.8%) | 364 (41.4%) | 369 (43.3%) | 659 (41.8%) | 703 (45.8%) |
| Situation 6 (Older student smoking outside school) | | | | | | |
| *Mean (SD)* | -0.6 (0.4) | -0.5 (0.4) | -0.5 (0.4) | -0.5 (0.4) | -0.6 (0.4) | -0.5 (0.4) |
| *Median (IQR)* | -0.6 (-1.0 to -0.2) | -0.6 (-1.0 to -0.2) | -0.6 (-1.0 to -0.2) | -0.6 (-1.0 to -0.2) | -0.6 (-1.0 to -0.2) | -0.6 (-1.0 to -0.2) |
| *Modal response, n (%)* | 258 (37.3%) | 273 (40.0%) | 349 (39.7%) | 413 (48.5%) | 607 (38.6%) | 686 (44.7%) |
| Situation 7 (School pupil using an e-cigarette whilst walking to school) | | | | | | |
| *Mean (SD)* | -0.5 (0.4) | -0.5 (0.4) | -0.6 (0.4) | -0.5 (0.4) | -0.5 (0.4) | -0.5 (0.4) |
| *Median (IQR)* | -0.6 (-1.0 to -0.2) | -0.6 (-0.6 to -0.2) | -0.6 (-1.0 to -0.2) | -0.6 (-1.0 to -0.2) | -0.6 (-1.0 to -0.2) | -0.6 (-1.0 to -0.2) |
| *Modal response, n (%)* | 263 (37.8%) | 273 (40.0%) | 373 (42.4%) | 333 (39.1%) | 636 (40.4%) | 606 (39.5%) |
| Situation 8 (School pupil sharing a photograph of his/her e-cigarette use on social media) | | | | | | |
| *Mean (SD)* | -0.5 (0.4) | -0.5 (0.4) | -0.5 (0.4) | -0.5 (0.4) | -0.5 (0.4) | -0.5 (0.4) |
| *Median (IQR)* | -0.6 (-1.0 to -0.2) | -0.6 (-0.6 to -0.2) | -0.6 (-1.0 to -0.2) | -0.6 (-1.0 to -0.2) | -0.6 (-1.0 to -0.2) | -0.6 (-0.8 to -0.2) |
| *Modal response, n (%)* | 255 (36.7%) | 266 (38.9%) | 389 (44.2%) | 346 (40.6%) | 644 (40.9%) | 612 (39.8%) |
| Situation 9 (School pupil chewing tobacco) | | | | | | |
| *Mean (SD)* | -0.8 (0.4) | -0.7 (0.4) | -0.8 (0.3) | -0.8 (0.3) | -0.8 (0.3) | -0.7 (0.3) |
| *Median (IQR)* | -1.0 (-1.0 to -0.6) | -1.0 (-1.0 to -0.6) | -1.0 (-1.0 to -0.6) | -1.0 (-1.0 to -0.6) | -1.0 (-1.0 to -0.6) | -1.0 (-1.0 to -0.6) |
| *Modal response, n (%)* | 427 (61.4%) | 355 (52.0%) | 591 (67.2%) | 503 (59.0%) | 1018 (64.6%) | 858 (55.9%) |
| **Experiment Part 3 (descriptive social norms)^c^** | | | | | | |
| Q1 (Proportion of peers who would be accepting of a close friend smoking) | | | | | | |
| *Mean (SD)* | -0.5 (0.5) | -0.4 (0.5) | -0.5 (0.5) | -0.4 (0.5) | -0.5 (0.5) | -0.4 (0.5) |
| *Median (IQR)* | -0.6 (-1.0 to -0.2) | -0.6 (-0.6 to -0.2) | -0.6 (-1.0 to -0.2) | -0.6 (-0.6 to -0.2) | -0.6 (-1.0 to -0.2) | -0.6 (-0.6 to -0.2) |
| *Modal response, n (%)* | 268 (38.6%) | 248 (36.3%) | 367 (41.7%) | 390 (45.8%) | 635 (40.3%) | 638 (41.5%) |
| Q2 (Proportion of peers who would be accepting of a close friend vaping) | | | | | | |
| *Mean (SD)* | -0.3 (0.6) | -0.2 (0.6) | -0.5 (0.5) | -0.3 (0.6) | -0.4 (0.5) | -0.3 (0.6) |
| *Median (IQR)* | -0.6 (-0.6 to 0.2) | -0.2 (-0.6 to 0.2) | -0.6 (-1.0 to -0.2) | -0.6 (-0.6 to -0.2) | -0.6 (-1.0 to -0.2) | -0.2 (-0.6 to 0.2) |
| *Modal response, n (%)* | 220 (31.7%) | 206 (30.1%) | 403 (45.8%) | 304 (35.7%) | 623 (39.6%) | 510 (33.2%) |
| **Experiment Part 4 (number of tokens donated to ASSIST/Dead Cool; 0-10)** | | | | | | |
| *Mean (SD)* | 3.5 (3.1) | 3.1 (2.8) | 4.0 (2.5) | 3.8 (2.5) | 3.8 (2.8) | 3.4 (2.7) |
| *Median (IQR)* | 3.0 (1.0 to 5.0) | 3.0 (0.0 to 5.0) | 5.0 (2.0 to 5.0) | 5.0 (2.0 to 5.0) | 4.0 (1.0 to 5.0) | 4.0 (1.0 to 5.0) |
| **Survey: Smoking behavior, intentions, and attitudes** | | | | | | |
| Smoking behavior, n (%)^d^ | | | | | | |
| *Mean (SD)* | 3.8 (0.6) | 3.8 (0.7) | 3.7 (0.7) | 3.7 (0.7) | 3.8 (0.6) | 3.7 (0.7) |
| *Sometimes smoke* | 13 (1.9%) | 20 (3.1%) | 14 (1.6%) | 12 (1.4%) | 27 (1.7%) | 32 (2.1%) |
| *Previous smoker* | 22 (3.1%) | 27 (4.1%) | 71 (8.1%) | 79 (9.3%) | 93 (5.9%) | 106 (7.1%) |
| *Smoked once* | 48 (6.9%) | 46 (7.0%) | 73 (8.4%) | 91 (10.8%) | 121 (7.7%) | 137 (9.1%) |
| *Never smoked* | 618 (88.2%) | 561 (85.8%) | 714 (81.9%) | 664 (78.5%) | 1332 (84.7%) | 1225 (81.7%) |
| Intent to take up smoking in the next 6 months, n(%)^e^ | | | | | | |
| *Mean (SD)* | 5.7 (0.8) | 5.6 (0.9) | 5.5 (1.2) | 5.3 (1.3) | 5.6 (1.1) | 5.5 (1.2) |
| *I am a smoker* | 10 (1.4%) | 13 (2.0%) | 39 (4.5%) | 48 (5.7%) | 49 (3.1%) | 61 (4.1%) |
| *Definitely start smoking* | 1 (0.1%) | 1 (0.2%) | 2 (0.2%) | - | 3 (0.2%) | 1 (0.1%) |
| *Probably start smoking* | - | 3 (0.5%) | 8 (0.9%) | 14 (1.7%) | 8 (0.5%) | 17 (1.1%) |
| *Don’t know* | 49 (7.0%) | 54 (8.3%) | 92 (10.6%) | 115 (13.6%) | 141 (9.0%) | 169 (11.3%) |
| *Probably remain a non-smoker* | 45 (6.5%) | 42 (6.5%) | 59 (6.8%) | 76 (9.0%) | 104 (6.6%) | 118 (7.9%) |
| *Definitely remain a non-smoker* | 592 (84.9%) | 538 (82.6%) | 672 (77.1%) | 593 (70.1%) | 1264 (80.6%) | 1131 (75.6%) |
| **Survey: Self-reported injunctive social norms, n(%)^f^** | | | | | | |
| Most of the people who are important to me think that I… | | | | | | |
| *Mean (SD)* | 1.7 (0.7) | 1.7 (0.7) | 1.8 (0.6) | 1.7 (0.7) | 1.8 (0.7) | 1.7 (0.7) |
| *Definitely should smoke-Maybe should not smoke* | 101 (14.6%) | 105 (16.3%) | 128 (14.7%) | 144 (17.0%) | 229 (14.6%) | 249 (16.7%) |
| *Definitely should not smoke* | 593 (85.5%) | 540 (83.7%) | 744 (85.3%) | 702 (83.0%) | 1337 (85.4%) | 1242 (83.3%) |
| My mother thinks that I… | | | | | | |
| *Mean (SD)* | 1.9 (0.3) | 1.9 (0.4) | 1.9 (0.4) | 1.9 (0.5) | 1.9 (0.4) | 1.9 (0.4) |
| *Definitely should smoke-Maybe should not smoke* | 28 (4.0%) | 26 (4.0%) | 53 (6.1%) | 62 (7.3%) | 81 (5.2%) | 88 (5.9%) |
| *Definitely should not smoke* | 663 (95.4%) | 618 (95.7%) | 815 (93.5%) | 779 (92.1%) | 1478 (94.3%) | 1397 (93.6%) |
| *I don't have a mother* | 4 (0.6%) | 2 (0.3%) | 4 (0.5%) | 5 (0.6%) | 8 (0.5%) | 7 (0.5%) |
| My father thinks that I… | | | | | | |
| *Mean (SD)* | 1.8 (0.6) | 1.8 (0.6) | 1.7 (0.7) | 1.7 (0.7) | 1.7 (0.7) | 1.7 (0.7) |
| *Definitely should smoke-Maybe should not smoke* | 35 (5.1%) | 44 (6.8%) | 93 (10.7%) | 106 (12.5%) | 128 (8.2%) | 150 (10.1%) |
| *Definitely should not smoke* | 627 (90.6%) | 575 (89.2%) | 701 (80.4%) | 683 (80.7%) | 1328 (84.9%) | 1258 (84.4%) |
| *I don't have a father* | 30 (4.3%) | 26 (4.0%) | 78 (8.9%) | 57 (6.7%) | 108 (6.9%) | 83 (5.6%) |
| My brother(s) think(s) that I… | | | | | | |
| *Mean (SD)* | 1.4 (0.9) | 1.4 (0.9) | 1.4 (0.9) | 1.5 (0.8) | 1.4 (0.9) | 1.4 (0.9) |
| *Definitely should smoke-Maybe should not smoke* | 81 (11.7%) | 87 (13.5%) | 159 (18.2%) | 133 (15.7%) | 240 (15.4%) | 220 (14.8%) |
| *Definitely should not smoke* | 465 (67.3%) | 430 (66.7%) | 583 (66.9%) | 605 (71.5%) | 1048 (67.1%) | 1035 (69.4%) |
| *I don't have a brother* | 145 (21.0%) | 128 (19.8%) | 130 (14.9%) | 108 (12.8%) | 275 (17.6%) | 236 (15.8%) |
| My sister(s) think(s) that I… | | | | | | |
| *Mean (SD)* | 1.4 (0.9) | 1.4 (0.9) | 1.4 (0.9) | 1.4 (0.9) | 1.4 (0.9) | 1.4 (0.9) |
| *Definitely should smoke-Maybe should not smoke* | 54 (7.8%) | 63 (9.8%) | 125 (14.3%) | 112 (13.2%) | 179 (11.4%) | 175 (11.7%) |
| *Definitely should not smoke* | 476 (68.7%) | 432 (67.0%) | 570 (65.4%) | 586 (69.3%) | 1046 (66.8%) | 1018 (68.3%) |
| *I don't have a sister* | 163 (23.5%) | 150 (23.3%) | 177 (20.3%) | 148 (17.5%) | 340 (21.7%) | 298 (20.0%) |
| My friends think that I… | | | | | | |
| *Mean (SD)* | 1.5 (0.9) | 1.5 (0.9) | 1.3 (1.0) | 1.3 (0.9) | 1.4 (0.9) | 1.4 (0.9) |
| *Definitely should smoke-Maybe should not smoke* | 207 (29.8%) | 169 (26.2%) | 347 (39.8%) | 346 (40.9%) | 554 (35.4%) | 515 (34.5%) |
| *Definitely should not smoke* | 480 (69.2%) | 469 (72.7%) | 523 (60.0%) | 498 (58.9%) | 1003 (64.1%) | 967 (64.9%) |
| *I don't have a friend* | 7 (1.0%) | 7 (1.1%) | 2 (0.2%) | 2 (0.2%) | 9 (0.6%) | 9 (0.6%) |
| My best friend thinks that I… | | | | | | |
| *Mean (SD)* | 1.7 (0.7) | 1.6 (0.8) | 1.5 (0.9) | 1.5 (0.9) | 1.6 (0.8) | 1.6 (0.8) |
| *Definitely should smoke-Maybe should not smoke* | 115 (16.6%) | 116 (18.0%) | 220 (25.2%) | 211 (24.9%) | 335 (21.4%) | 327 (21.9%) |
| *Definitely should not smoke* | 568 (81.8%) | 520 (80.5%) | 607 (69.6%) | 592 (70.0%) | 1175 (75.0%) | 1112 (74.5%) |
| *I don't have a best friend* | 11 (1.6%) | 10 (1.6%) | 45 (5.2%) | 43 (5.1%) | 56 (3.6%) | 53 (3.6%) |
| **Survey: Self-reported descriptive social norms, n(%)^g^** | | | | | | |
| Does your best friend smoke? | | | | | | |
| *Mean (SD)* | 4.8 (0.8) | 4.7 (0.8) | 4.8 (0.7) | 4.8 (0.6) | 4.8 (0.7) | 4.8 (0.7) |
| *Very often-Rarely* | 72 (10.4%) | 83 (12.8%) | 80 (9.2%) | 80 (9.5%) | 152 (9.7%) | 163 (10.9%) |
| *Never/Don't know* | 603 (86.8%) | 533 (82.4%) | 704 (80.7%) | 686 (81.1%) | 1307 (83.4%) | 1219 (81.7%) |
| *I don't have a best friend* | 20 (2.9%) | 31 (4.8%) | 88 (10.1%) | 80 (9.5%) | 108 (6.9%) | 111 (7.4%) |
| Does your mother smoke? | | | | | | |
| *Mean (SD)* | 4.2 (1.4) | 4.3 (1.3) | 4.6 (1.0) | 4.6 (1.0) | 4.4 (1.2) | 4.5 (1.1) |
| *Very often-Rarely* | 206 (29.6%) | 181 (28.0%) | 159 (18.2%) | 144 (17.0%) | 365 (23.3%) | 325 (21.8%) |
| *Never/Don't know* | 486 (69.8%) | 461 (71.3%) | 708 (81.2%) | 697 (82.4%) | 1194 (76.2%) | 1158 (77.6%) |
| *I don't have a mother* | 4 (0.6%) | 5 (0.8%) | 5 (0.6%) | 5 (0.6%) | 9 (0.6%) | 10 (0.7%) |
| Does your father smoke? | | | | | | |
| *Mean (SD)* | 4.2 (1.4) | 4.2 (1.4) | 4.4 (1.2) | 4.4 (1.1) | 4.3 (1.3) | 4.3 (1.3) |
| *Very often-Rarely* | 204 (29.4%) | 189 (29.2%) | 217 (24.9%) | 209 (14.9%) | 421 (26.9%) | 398 (26.7%) |
| *Never/Don't know* | 453 (65.2%) | 421 (65.1%) | 570 (65.4%) | 571 (67.5%) | 1023 (65.3%) | 992 (66.4%) |
| *I don't have a father* | 38 (5.5%) | 37 (5.7%) | 85 (9.8%) | 66 (7.8%) | 123 (7.9%) | 103 (6.9%) |
| Do any of your brothers smoke? | | | | | | |
| *Mean (SD)* | 4.7 (0.9) | 4.7 (0.9) | 4.7 (0.8) | 4.7 (0.9) | 4.7 (0.9) | 4.7 (0.9) |
| *Very often-Rarely* | 47 (10.1%) | 79 (12.2%) | 105 (8.0%) | 111 (13.1%) | 175 (11.2%) | 190 (12.7%) |
| *Never/Don't know* | 451 (64.9%) | 415 (64.1%) | 592 (67.9%) | 588 (69.5%) | 1043 (66.6%) | 1003 (67.2%) |
| *I don't have a brother* | 174 (24.0%) | 153 (23.7%) | 175 (20.1%) | 147 (17.4%) | 349 (22.3%) | 300 (20.1%) |
| Do any of your sisters smoke? | | | | | | |
| *Mean (SD)* | 4.8 (0.7) | 4.8 (0.7) | 4.8 (0.7) | 4.8 (0.7) | 4.8 (0.7) | 4.8 (0.7) |
| *Very often-Rarely* | 47 (6.8%) | 51 (7.9%) | 70 (8.0%) | 68 (8.0%) | 117 (7.5%) | 119 (8.0%) |
| *Never/Don't know* | 458 (65.9%) | 415 (64.2%) | 563 (64.6%) | 558 (66.0%) | 1021 (65.2%) | 973 (65.2%) |
| *I don't have a sister* | 190 (27.3%) | 181 (28.0%) | 239 (27.4%) | 220 (26.0%) | 429 (27.4%) | 401 (26.9%) |
| **Survey: Psycho-social characteristics** | | | | | | |
| Need to Belong Scale^h^ | | | | | | |
| *Mean (SD)* | 3.1 (0.6) | - | 2.8 (0.6) | - | 3.0 (0.6) | - |
| *Median (IQR)* | 3.1 (2.7 to 3.5) | - | 2.8 (2.3 to 3.2) | - | 3.0 (2.5 to 3.4) | - |
| Fear of Negative Evaluation^i^ | | | | | | |
| *Mean (SD)* | 2.9 (0.7) | - | 2.6 (0.6) | - | 2.7 (0.7) | - |
| *Median (IQR)* | 2.8 (2.4 to 3.4) | - | 2.5 (2.3 to 2.9) | - | 2.7 (2.3 to 3.1) | - |
| Pro-social Behavior^j^ | | | | | | |
| *Mean (SD)* | 8.1 (2.1) | - | 7.3 (2.1) | - | 7.6 (2.1) | - |
| *Median (IQR)* | 9.0 (7.0 to 10.0) | - | 8.0 (6.0 to 9.0) | - | 8.0 (6.0 to 9.0) | - |
| Big 5 (Openness)^k^ | | | | | | |
| *Mean (SD)* | 2.4 (0.6) | - | 2.7 (0.7) | - | 2.6 (0.7) | - |
| *Median (IQR)* | 2.4 (2.0 to 2.9) | - | 2.7 (2.2 to 3.2) | - | 2.6 (2.1 to 3.1) | - |
| Big 5 (Extraversion)^k^ | | | | | | |
| *Mean (SD)* | 2.6 (0.8) | - | 2.7 (0.7) | - | 2.6 (0.7) | - |
| *Median (IQR)* | 2.6 (2.0 to 3.2) | - | 2.7 (2.2 to 3.2) | - | 2.6 (2.1 to 3.2) | - |
| Big 5 (Agreeableness)^k^ | | | | | | |
| *Mean (SD)* | 2.5 (0.6) | - | 2.6 (0.7) | - | 2.6 (0.7) | - |
| *Median (IQR)* | 2.5 (2.0 to 3.0) | - | 2.6 (2.1 to 3.1) | - | 2.5 (2.0 to 3.0) | - |
| Big 5 (Conscientiousness)^k^ | | | | | | |
| *Mean (SD)* | 2.3 (0.7) | - | 2.4 (0.6) | - | 2.4 (0.7) | - |
| *Median (IQR)* | 2.1 (1.9 to 2.7) | - | 2.3 (2.0 to 2.8) | - | 2.2 (1.9 to 2.8) | - |
| Big 5 (Stability)^k^ | | | | | | |
| *Mean (SD)* | 1.9 (0.8) | - | 2.1 (0.7) | - | 2.0 (0.7) | - |
| *Median (IQR)* | 1.9 (1.3 to 2.4) | - | 2.0 (1.6 to 2.5) | - | 2.0 (1.5 to 2.5) | - |
| **Smokerlyzer readings: Objective smoking behavior (expelled air carbon monoxide, ppm)^l^** | | | | | | |
| *Mean (SD)* | 1.5 (1.4) | 2.0 (1.0) | 3.4 (1.5) | 3.5 (1.7) | 2.5 (1.7) | 2.8 (1.6) |
| *Non-smoker (≤9 ppm), n (%)* | 590 (99.8%) | 591 (100.0%) | 643 (99.2%) | 614 (99.0%) | 1233 (99.5%) | 1205 (99.5%) |
| *Smoker (>9 ppm), n (%)* | 1 (0.2%) | 0 (0.0%) | 5 (0.8%) | 6 (1.0%) | 6 (0.5%) | 6 (0.5%) |

^a^Number of balls allocated to the blue (rule-following) or yellow (rule-breaking) buckets.

^b^-1=Extremely socially inappropriate; -0.6=Very socially inappropriate; -0.2=Somewhat socially inappropriate; 0.2=Somewhat socially appropriate; 0.6=Very socially appropriate; 1=Extremely socially appropriate.

^c^-1=None of my peers; -0.6=Only a few of my peers; -0.2=Some of my peers; +0.2=A lot of my peers; +0.6=Most of my peers; +1=All of my peers.

^d^1=Sometimes smoke; 2=Previous smoker; 3=Smoked once; 4=Never smoked.

^e^1=I am a smoker; 2=Definitely start smoking; 3=Probably start smoking; 4=Don't know; 5=Probably remain; 6=Definitely remain a non-smoker.

^f^-2=Definitely should smoke; -1=Maybe should smoke; 0=Don't know/neutral; +1=Maybe should not smoke; +2=Definitely should not smoke. "I don't have…" responses set to 0.

^g^1=Very often; 2=Often; 3=Occasionally; 4=Rarely; 5=Never/Don't know. “I don’t have…” responses set to 5.

^h^Average of 10 items, coded 1-5. Not available for two Colombian schools (excluded from analysis).

^i^Average of 12 items, coded 1-5. Not available for two Colombian schools (excluded from analysis).

^j^Sum of five items, coded 0-2.

^k^Average of 10 items, coded 0-4.

^l^Not available for one Northern Irish school and two Colombian schools (excluded from analysis).

**Experiment: Part 1 (Number of balls allocated to blue and yellow buckets, blue=rule-following)**

**Experiment: Part 2, Situation 2 (Parent smoking at home in front of young children)**

**Experiment: Part 2, Situation 3 (Adult smoking in car with children onboard)**

**Experiment: Part 2, Situation 4 (Someone selling cigarettes without proof of age)**

**Experiment: Part 2, Situation 5 (Movie showing the lead character smoking)**

**Experiment: Part 2, Situation 6 (Older student smoking outside school)**

**Experiment: Part 2, Situation 7 (School student smoking an e-cigarette)**

**Experiment: Part 2, Situation 8 (School student sharing a photo of his/her e-cigarette use)**

**Experiment: Part 2, Situation 9 (School student chewing tobacco)**

**Experiment: Part 3, Q1 (Proportion of peers accepting of a close friend smoking)**

**Experiment: Part 3, Q2 (Proportion of peers accepting of a close friend vaping)**

**Experiment Part 4: Number of tokens donated**

**Survey: Injunctive norms 1 (Most of the people who are important to me think that I…)**

**Survey: Injunctive norms 2 (My mother thinks that I…)**

**Survey: Injunctive norms 3 (My father thinks that I…)**

**Survey: Injunctive norms 4 (My brother(s) think(s) that I…)**

**Survey: Injunctive norms 5 (My sister(s) think(s) that I…)**

**Survey: Injunctive norms 6 (My friends think that I…)**

**Survey: Injunctive norms 7 (My best friend thinks that I…)**

**Survey: Descriptive norms 1 (Does your best friend smoke?)**

**Survey: Descriptive norms 2 (Does your mother smoke?)**

**Survey: Descriptive norms 3 (Does your father smoke?)**

**Survey: Descriptive norms 4 (Do any of your brothers smoke?)**

**Survey: Descriptive norms 5 (Do any of your sisters smoke?)**

**Survey: Past smoking behavior (1=Sometimes smoke; 2=Previous smoker; 3=Smoked once; 4=Never smoked)**

**Survey: Smoking intentions (1=I am a smoker; 2=Definitely start smoking; 3=Probably start smoking; 4=Don't know; 5=Probably remain; 6=Definitely remain a non-smoker)**

**Smokerlyzer readings: Objective smoking behavior (expelled air carbon monoxide, parts per million; ppm). Not available for one Northern Irish school and two Colombian schools.**

**Supplement 5: Spearman's rank-order correlations and results of mixed-effects regressions**

**Methods for correlational analyses and mixed-effects regressions**

As a first step towards establishing a basis for construct validity, Spearman's rank-order correlations were computed, examining the association between individual items from the experiments and survey, separately for injunctive and descriptive norm items. Spearman's rank-order correlations were also computed examining the associations between self-reported anti-smoking behavior and intentions, and objectively measured smoking behavior. Next, individual items from the experiment and survey were examined for an association with self-reported anti-smoking behavior and intentions using mixed-effects ordered logistic regressions. Individual items from the experiment and survey were examined for an association with objectively measured smoking behavior derived from the Smokerlyzer readings using mixed-effects linear regressions with robust standard errors (i.e. examining individual items in relation to an external criteria). In each model either anti-smoking behavior, anti-smoking intentions, or objectively measured smoking behavior (expelled air carbon monoxide in parts per million; ppm) was the dependent variable, and individual injunctive or descriptive norm items from the experiment or survey were the independent variables. Models were also adjusted for sex (boy, girl/Prefer not to say), intervention (ASSIST, Dead Cool) and country (NI, Colombia). Models including items from the experiment were adjusted for rule-following (number of balls allocated to the blue bucket in Part 1). Each model had three levels, including random effects modelling classes within schools. For the mixed-effects ordered logistic regression models (including self-reported anti-smoking behavior or intentions as the dependent variable), analyses were conducted using Stata's 'meologit' command. Reported results are odds ratios (ORs) and 95% confidence intervals (CIs). The proportional odds assumption was tested for each model using Stata's 'omodel' module which performs an approximate likelihood-ratio test of proportionality of odds across response categories (p<0.05 indicated a potential violation of the proportional odds assumption). These models were re-run as mixed-effects linear regressions using Stata's 'mixed' command with no major differences to the results (data not presented). For the mixed effects linear regression models (including objective smoking behavior as the dependent variable), analyses were conducted using Stata’s ‘mixed’ command with the ‘vce(robust)’ option specified. Reported results are regression coefficients (*b*) and 95% CIs. Results should be interpreted with caution due to multiple testing. However, our objectives were to provide a preliminary appraisal of whether there were theoretically expected relationships between individual item scores and between individual items and relevant external criteria.

**Results of correlational analyses and mixed-effects regressions**

Correlation matrices showing Spearman's rank-order correlations between individual items from the experiments and survey are shown below. All correlations between items from different instruments (experiment vs. survey; located in the bottom left-hand side of tables) with p<0.05 are negative. Most correlations between individual items from the same instrument (experiment or survey) are positive with p<0.01. A correlation matrix is also shown with Spearman's rank-order correlations between self-reported anti-smoking behavior and intentions, and objectively measured smoking behavior. The correlations between self-reported anti-smoking behavior and objective smoking behavior are negative with p<0.01. The correlation between self-reported anti-smoking behavior and self-reported anti-smoking intentions is positive with p<0.01.

These results are as expected since numerically the experiment and survey norms are coded in intuitively the opposite direction. Experiment injunctive norms items are coded such that higher numerical values equate to higher perceptions that situations involving smoking or vaping are socially appropriate. By contrast, survey injunctive norm items are coded such that higher numerical values equate to higher perceptions that others who are important to you think that you should **not** smoke. Experiment descriptive norms are coded such that higher numerical values equate to higher perceptions that a greater number of your peers would be accepting of smoking or vaping behaviors by others. By contrast, survey descriptive norms are coded such that higher numerical values equate to higher perceptions that others who are important to you engage in smoking behaviors **less** often. Self-reported anti-smoking behaviors and intentions from the survey are coded such that higher numerical values equate to **less** smoking behavior in the past or greater intentions **not** to smoke. Higher values on the objectively measured smoking behavior variable indicate readings with greater levels of expelled air carbon monoxide in ppm (a reading of >9ppm indicates smoking behavior within the past 24 hours).

Results of mixed-effects ordered logistic regressions and mixed effects linear regressions are also shown below. The following items from the experiment showed an association with self-reported anti-smoking behavior (p<0.05): P2Sit4, P2Sit7, P2Sit8, P2Sit9, P3Q1, P3Q2 (ORs 0.46-0.62). The following experiment items showed an association with self-reported anti-smoking intentions (p<0.05): P2Sit3, P2Sit4, P2Sit6, P2Sit7, P2Sit8, P2Sit9, P3Q1, P3Q2 (ORs 0.45-0.69). None of the individual items from the experiment showed associations with objectively measured smoking behavior. The results of the ordered logistic regression models may be interpreted as proportional ORs representing the multiplicative change in odds of at least a one-unit increase in response category to the dependent variable (more self-reported anti-smoking behavior/greater intentions not to smoke) for a one-unit increase in response category to the relevant norm question (increasingly socially appropriate/increasing perceptions that more peers would be accepting of a close friend smoking or vaping), holding other variables constant. Since the OR values are all <1.0 for self-reported outcomes, this indicates that pupils who gave higher social appropriateness ratings for situations involving smoking or vaping, and pupils who thought that more peers would be accepting of a close friend smoking or vaping were more likely to give answers geared away from anti-smoking behavior and intentions. The results of these models also indicate that pupils who were more rule-following in Part 1 were more likely to give answers geared towards anti-smoking behavior and intentions (ORs>1.0, p<0.01 for self-reported outcomes). This is as expected since there were already anti-smoking norms established in the schools at baseline and rule-following individuals are expected to be more sensitive to norms. Finally, these models indicate that Colombian pupils were more likely to provide answers geared away from anti-smoking behavior and intentions or to have higher levels of expelled air carbon monoxide in their Smokerlyzer readings (ORs<1.0, p<0.01 for self-reported outcomes; *b*s>0, p<0.01 for objectively measured outcomes), and that pupils in Dead Cool schools had lower levels of expelled air carbon monoxide in their Smokerlyzer readings at baseline (p<0.05).

Most of the injunctive and descriptive norm items from the survey showed associations with self-reported anti-smoking behavior and anti-smoking intentions (ORs 1.25-2.49, p<0.01). These results may be interpreted as proportional ORs representing the multiplicative change in odds of at least a one-unit increase in response category to the dependent variable (more self-reported anti-smoking behavior/greater intentions not to smoke) for a one-unit increase in response category to the relevant norm question (increasing perceptions that important others think you should not smoke/increasing perceptions that important others do not smoke often), holding other variables constant. Since the OR values are all >1.0, this indicates that pupils who had higher perceptions that others who are important to them think they should not smoke or that others who are important to them do not smoke often were more likely to give answers geared towards anti-smoking behavior and intentions. There was an association between item IN7 and objectively measured smoking behavior (*b*=-0.08, p<0.05). Again, these models showed that Colombian pupils were more likely to provide answers geared away from anti-smoking behavior and intentions or to have higher levels of expelled air carbon monoxide in their Smokerlyzer readings (ORs<1.0, p<0.05 for self-reported outcomes; *b*s>0, p<0.01 for objectively measured outcomes), and that pupils in Dead Cool schools had lower levels of expelled air carbon monoxide in their Smokerlyzer readings at baseline (p<0.05).

In summary, individual items from the experiment and survey are showing theoretically expected inter-relationships and associations with self-reported anti-smoking behavior and intentions. Most items showed non-significant associations with objectively measured smoking behavior derived from the Smokerlyzer readings.

**Spearman's rank-order correlations between experiment and survey measures of injunctive norms for smoking and vaping**

|  | | **Expt. measures** | | | | | | | | **Survey measures** | | | | | | |
| --- | --- | --- | --- | --- | --- | --- | --- | --- | --- | --- | --- | --- | --- | --- | --- | --- |
|  |  | **(1)** | **(2)** | **(3)** | **(4)** | **(5)** | **(6)** | **(7)** | **(8)** | **(9)** | **(10)** | **(11)** | **(12)** | **(13)** | **(14)** | **(15)** |
|  |  | **P2S2** | **P2S3** | **P2S4** | **P2S5** | **P2S6** | **P2S7** | **P2S8** | **P2S9** | **IN1** | **IN2** | **IN3** | **IN4** | **IN5** | **IN6** | **IN7** |
| **Expt.** | **(1)** | 1.00 |  |  |  |  |  |  |  |  |  |  |  |  |  |  |
|  | **(2)** | 0.29*** | 1.00 |  |  |  |  |  |  |  |  |  |  |  |  |  |
|  | **(3)** | 0.18*** | 0.24*** | 1.00 |  |  |  |  |  |  |  |  |  |  |  |  |
|  | **(4)** | 0.21*** | 0.24*** | 0.20*** | 1.00 |  |  |  |  |  |  |  |  |  |  |  |
|  | **(5)** | 0.13*** | 0.27*** | 0.27*** | 0.28*** | 1.00 |  |  |  |  |  |  |  |  |  |  |
|  | **(6)** | 0.18*** | 0.31*** | 0.28*** | 0.33*** | 0.45*** | 1.00 |  |  |  |  |  |  |  |  |  |
|  | **(7)** | 0.11*** | 0.24*** | 0.27*** | 0.35*** | 0.43*** | 0.56*** | 1.00 |  |  |  |  |  |  |  |  |
|  | **(8)** | 0.16*** | 0.23*** | 0.24*** | 0.21*** | 0.28*** | 0.33*** | 0.36*** | 1.00 |  |  |  |  |  |  |  |
| **Survey** | **(9)** | -0.03 | -0.04 | -0.13*** | -0.03 | -0.11*** | -0.12*** | -0.15*** | -0.04* | 1.00 |  |  |  |  |  |  |
|  | **(10)** | -0.01 | -0.02 | -0.04 | -0.001 | -0.04* | -0.06** | -0.07*** | 0.01 | 0.35*** | 1.00 |  |  |  |  |  |
|  | **(11)** | 0.02 | -0.05** | -0.07*** | -0.03 | -0.06** | -0.06** | -0.08*** | -0.03 | 0.23*** | 0.41*** | 1.00 |  |  |  |  |
|  | **(12)** | -0.04 | -0.09*** | -0.04 | -0.06** | -0.09*** | -0.10*** | -0.09*** | -0.07*** | 0.22*** | 0.26*** | 0.27*** | 1.00 |  |  |  |
|  | **(13)** | 0.003 | -0.06** | -0.06** | -0.01 | -0.05** | -0.05** | -0.05* | -0.01 | 0.19*** | 0.26*** | 0.27*** | 0.37*** | 1.00 |  |  |
|  | **(14)** | -0.01 | -0.07*** | -0.10*** | -0.12*** | -0.12*** | -0.15*** | -0.16*** | -0.10*** | 0.39*** | 0.26*** | 0.22*** | 0.32*** | 0.24*** | 1.00 |  |
|  | **(15)** | -0.01 | -0.08*** | -0.13*** | -0.07*** | -0.12*** | -0.11*** | -0.16*** | -0.10*** | 0.37*** | 0.29*** | 0.24*** | 0.28*** | 0.23*** | 0.63*** | 1.00 |

*******p<0.01; **p<0.05; *p<0.10.

**Spearman's rank-order correlations between experiment and survey measures of descriptive norms for smoking and vaping**

|  | | **Expt. measures** | | **Survey measures** | | | | |
| --- | --- | --- | --- | --- | --- | --- | --- | --- |
|  |  | **(1)** | **(2)** | **(3)** | **(4)** | **(5)** | **(6)** | **(7)** |
|  |  | **P3Q1** | **P3Q2** | **DN1** | **DN2** | **DN3** | **DN4** | **DN5** |
| **Expt.** | **(1)** | 1.00 |  |  |  |  |  |  |
|  | **(2)** | 0.73*** | 1.00 |  |  |  |  |  |
| **Survey** | **(3)** | -0.15*** | -0.16*** | 1.00 |  |  |  |  |
|  | **(4)** | -0.07*** | -0.10*** | 0.14*** | 1.00 |  |  |  |
|  | **(5)** | -0.07*** | -0.10*** | 0.11*** | 0.35*** | 1.00 |  |  |
|  | **(6)** | -0.04 | -0.04 | 0.16*** | 0.13*** | 0.10*** | 1.00 |  |
|  | **(7)** | -0.03 | -0.03 | 0.16*** | 0.17*** | 0.12*** | 0.23*** | 1.00 |

*******p<0.01; **p<0.05; *p<0.10.

**Spearman's rank-order correlations between self-reported anti-smoking behavior and intentions, and objectively measured smoking behavior**

|  | **Anti-smoking behavior** | **Anti-smoking intentions** | **Objective smoking behavior** |
| --- | --- | --- | --- |
|  | **(1)** | **(2)** | **(3)** |
| **(1)** | 1.00 |  |  |
| **(2)** | 0.40*** | 1.00 |  |
| **(3)** | -0.11*** | -0.08*** | 1.00 |

*******p<0.01; **p<0.05; *p<0.10.**Results of mixed-effects ordered logistic regressions showing relationship between anti-smoking behavior and responses to smoking norm questions Experiment Parts 2-3**

|  | **Dependent variable: Anti-smoking behavior** | | | | | | | | | |
| --- | --- | --- | --- | --- | --- | --- | --- | --- | --- | --- |
|  | **P2S2** | **P2S3** | **P2S4** | **P2S5** | **P2S6** | **P2S7** | **P2S8** | **P2S9** | **P3Q1** | **P3Q2** |
|  | *OR* (95% CI) | *OR* (95% CI) | *OR* (95% CI) | *OR* (95% CI) | *OR* (95% CI) | *OR* (95% CI) | *OR* (95% CI) | *OR* (95% CI) | *OR* (95% CI) | *OR* (95% CI) |
| **n** | 1512 | 1512 | 1513 | 1513 | 1509 | 1513 | 1512 | 1513 | 1513 | 1511 |
| **Norm^a^** | 1.04  (0.59, 1.84) | 0.84  (0.55, 1.27) | 0.62**  (0.42, 0.90) | 0.73*  (0.52, 1.03) | 0.74*  (0.52, 1.04) | 0.54***  (0.39, 0.74) | 0.50***  (0.36, 0.71) | 0.58***  (0.39, 0.86) | 0.46***  (0.35, 0.60) | 0.46***  (0.36, 0.60) |
| **Blue bucket** | 1.01***  (1.005, 1.02) | 1.01***  (1.005, 1.02) | 1.01***  (1.005, 1.02) | 1.01***  (1.005, 1.02) | 1.01***  (1.005, 1.02) | 1.01***  (1.005, 1.02) | 1.01***  (1.01, 1.02) | 1.01***  (1.005, 1.02) | 1.01***  (1.005, 1.02) | 1.01***  (1.004, 1.02) |
| **Sex** |  |  |  |  |  |  |  |  |  |  |
| *Girl/PNTS* | 1.06  (0.79, 1.42) | 1.08  (0.81, 1.45) | 1.05  (0.78, 1.41) | 1.04  (0.78, 1.40) | 1.08  (0.80, 1.44) | 1.03  (0.76, 1.38) | 1.07  (0.80, 1.44) | 1.04  (0.78, 1.40) | 1.2  (0.83, 1.50) | 1.06  (0.79, 1.42) |
| **Intervention** |  |  |  |  |  |  |  |  |  |  |
| *Dead Cool* | 1.28  (0.75, 2.18) | 1.27  (0.75, 2.18) | 1.26  (0.74, 2.13) | 1.30  (0.76, 2.23) | 1.27  (0.75, 2.16) | 1.25  (0.74, 2.12) | 1.24  (0.73, 2.13) | 1.30  (0.77, 2.19) | 1.19  (0.71, 1.99) | 1.22  (0.73, 2.02) |
| **Country** |  |  |  |  |  |  |  |  |  |  |
| *Colombia* | 0.47***  (0.28, 0.81) | 0.46***  (0.27, 0.79) | 0.47***  (0.28, 0.80) | 0.45***  (0.26, 0.77) | 0.48***  (0.28, 0.82) | 0.46***  (0.27, 0.77) | 0.48***  (0.28, 0.83) | 0.46***  (0.27, 0.78) | 0.44***  (0.26, 0.74) | 0.42***  (0.25, 0.70) |

^a^Note on interpretation: these are proportional odds ratios representing the multiplicative change in odds of **at least** a one-unit increase in response category to the smoking behavior question (more anti-smoking) for a one-unit increase in response category to the relevant norm question (increasingly socially appropriate/increasing perceptions that more peers would be accepting of a close friend smoking or vaping), holding other variables constant. P2 Norms coded (-1=Extremely socially inappropriate; -0.6=Very socially inappropriate; -0.2=Somewhat socially inappropriate; +0.2=Somewhat socially appropriate; +0.6=Very socially appropriate; +1=Extremely socially appropriate). P3 Norms coded (-1=None of my peers; -0.6=Only a few of my peers; -0.2=Some of my peers; +0.2=A lot of my peers; +0.6=Most of my peers; +1=All of my peers).

*******p<0.01; **p<0.05; *p<0.10.

**Results of mixed-effects ordered logistic regressions showing relationship between anti-smoking intentions and responses to smoking norm questions Experiment Parts 2-3**

|  | **Dependent variable: Anti-smoking intentions** | | | | | | | | | |
| --- | --- | --- | --- | --- | --- | --- | --- | --- | --- | --- |
|  | **P2S2** | **P2S3** | **P2S4** | **P2S5** | **P2S6** | **P2S7** | **P2S8** | **P2S9** | **P3Q1** | **P3Q2** |
|  | *OR* (95% CI) | *OR* (95% CI) | *OR* (95% CI) | *OR* (95% CI) | *OR* (95% CI) | *OR* (95% CI) | *OR* (95% CI) | *OR* (95% CI) | *OR* (95% CI) | *OR* (95% CI) |
| **n** | 1508 | 1508 | 1509 | 1509 | 1505 | 1509 | 1508 | 1509 | 1509 | 1507 |
| **Norm^a^** | 0.77  (0.48, 1.24) | 0.68**  (0.47, 0.98) | 0.62***  (0.44, 0.89) | 0.74*  (0.55, 1.01) | 0.69**  (0.51, 0.95) | 0.56***  (0.42, 0.76) | 0.45***  (0.33, 0.62) | 0.68**  (0.47, 0.99) | 0.53***  (0.41, 0.68) | 0.56***  (0.44, 0.71) |
| **Blue bucket** | 1.01**  (1.001, 1.02) | 1.01**  (1.001, 1.02) | 1.01**  (1.001, 1.02) | 1.01**  (1.001, 1.02) | 1.01**  (1.001, 1.02) | 1.01**  (1.001, 1.02) | 1.01**  (1.002, 1.02) | 1.01**  (1.001, 1.02) | 1.01**  (1.001, 1.02) | 1.01**  (1.001, 1.02) |
| **Sex** |  |  |  |  |  |  |  |  |  |  |
| *Girl/PNTS* | 0.93  (0.72, 1.22) | 0.92  (0.71, 1.21) | 0.91  (0.70, 1.19) | 0.91  (0.70, 1.19) | 0.93  (0.71, 1.21) | 0.90  (0.69, 1.17) | 0.93  (0.71, 1.22) | 0.91  (0.70, 1.19) | 0.96  (0.74, 1.26) | 0.92  (0.70, 1.20) |
| **Intervention** |  |  |  |  |  |  |  |  |  |  |
| *Dead Cool* | 1.08  (0.70, 1.67) | 1.08  (0.70, 1.66) | 1.07  (0.70, 1.62) | 1.09  (0.71, 1.68) | 1.05  (0.69, 1.61) | 1.05  (0.68, 1.62) | 1.03  (0.67, 1.60) | 1.09  (0.71, 1.67) | 1.02  (0.68, 1.54) | 1.04  (0.69, 1.58) |
| **Country** |  |  |  |  |  |  |  |  |  |  |
| *Colombia* | 0.48***  (0.31, 0.75) | 0.49***  (0.31, 0.75) | 0.50***  (0.33, 0.77) | 0.48***  (0.31, 0.74) | 0.50***  (0.33, 0.78) | 0.48***  (0.31, 0.75) | 0.51***  (0.33, 0.80) | 0.49***  (0.32, 0.76) | 0.48***  (0.31, 0.72) | 0.46***  (0.30, 0.70) |

^a^Note on interpretation: these are proportional odds ratios representing the multiplicative change in odds of **at least** a one-unit increase in response category to the smoking intentions question (greater intentions not to smoke) for a one-unit increase in response category to the relevant norm question (increasingly socially appropriate/increasing perceptions that more peers would be accepting of a close friend smoking or vaping), holding other variables constant. P2 Norms coded (-1=Extremely socially inappropriate; -0.6=Very socially inappropriate; -0.2=Somewhat socially inappropriate; +0.2=Somewhat socially appropriate; +0.6=Very socially appropriate; +1=Extremely socially appropriate). P3 Norms coded (-1=None of my peers; -0.6=Only a few of my peers; -0.2=Some of my peers; +0.2=A lot of my peers; +0.6=Most of my peers; +1=All of my peers).

*******p<0.01; **p<0.05; *p<0.10.

**Results of mixed-effects linear regressions showing relationship between objectively measured smoking behavior and responses to smoking norm questions Experiment Parts 2-3**

|  | **Dependent variable: Objective smoking behavior (expelled air carbon monoxide, ppm)^b^** | | | | | | | | | |
| --- | --- | --- | --- | --- | --- | --- | --- | --- | --- | --- |
|  | **P2S2** | **P2S3** | **P2S4** | **P2S5** | **P2S6** | **P2S7** | **P2S8** | **P2S9** | **P3Q1** | **P3Q2** |
|  | *b* (95% CI) | *b* (95% CI) | *b* (95% CI) | *b* (95% CI) | *b* (95% CI) | *b* (95% CI) | *b* (95% CI) | *b* (95% CI) | *b* (95% CI) | *b* (95% CI) |
| **n** | 1158 | 1157 | 1158 | 1158 | 1155 | 1158 | 1157 | 1158 | 1158 | 1157 |
| **Norm^a^** | 0.22  (-0.24, 0.69) | 0.22*  (-0.04, 0.49) | 0.18  (-0.09, 0.45) | 0.04  (-0.20, 0.28) | 0.06  (-0.06, 0.19) | -0.007  (-0.18, 0.17) | 0.02  (-0.21, 0.25) | 0.15  (-0.10, 0.40) | 0.19*  (-0.02, 0.39) | 0.15*  (-0.03, 0.33) |
| **Blue bucket** | -0.0002  (-0.005, 0.004) | 0.00001  (-0.004, 0.004) | -0.0001  (-0.005, 0.004) | -0.0001  (-0.005, 0.004) | -0.00005  (-0.005, 0.004) | -0.0001  (-0.005, 0.004) | -0.00002  (-0.005, 0.005) | 0.00002  (-0.004, 0.004) | -0.00007  (-0.005, 0.004) | 0.00001  (-0.005, 0.004) |
| **Sex** |  |  |  |  |  |  |  |  |  |  |
| *Girl/PNTS* | -0.02  (-0.18, 0.14) | -0.02  (-0.18, 0.14) | -0.01  (-0.17, 0.15) | -0.01  (-0.18, 0.15) | -0.01  (-0.17, 0.14) | -0.02  (-0.18, 0.14) | -0.02  (-0.17, 0.14) | -0.09  (-0.17, 0.15) | -0.03  (-0.18, 0.13) | -0.02  (-0.17, 0.14) |
| **Intervention** |  |  |  |  |  |  |  |  |  |  |
| *Dead Cool* | -0.52**  (-0.97, -0.07) | -0.50**  (-0.95, -0.06) | -0.51**  (-0.95, -0.06) | -0.51**  (-0.96, -0.07) | -0.51**  (-0.96, -0.05) | -0.51**  (-0.96, -0.07) | -0.51**  (-0.96, -0.05) | -0.51**  (-0.96, -0.07) | -0.49**  (-0.95, -0.03) | -0.49**  (-0.94, -0.03) |
| **Country** |  |  |  |  |  |  |  |  |  |  |
| *Colombia* | 1.83***  (1.41, 2.26) | 1.82***  (1.37, 2.27) | 1.81***  (1.36, 2.27) | 1.82***  (1.35, 2.28) | 1.82***  (1.36, 2.27) | 1.81***  (1.36, 2.27) | 1.82***  (1.37, 2.26) | 1.82***  (1.36, 2.27) | 1.82***  (1.36, 2.28) | 1.83***  (1.37, 2.29) |
| **Constant** | 1.93***  (1.37, 2.50) | 1.89***  (1.54, 2.23) | 1.89***  (1.51, 2.27) | 1.75***  (1.52, 1.99) | 1.76***  (1.54, 1.99) | 1.74***  (1.50, 1.98) | 1.74***  (1.50, 1.98) | 1.85***  (1.51, 2.19) | 1.82***  (1.60, 2.04) | 1.77***  (1.56, 1.98) |

^a^Note on interpretation: these are regression coefficients representing the increase in carbon monoxide reading (more smoking) for a one-unit increase in response category to the relevant norm question (increasingly socially appropriate/increasing perceptions that more peers would be accepting of a close friend smoking or vaping), holding other variables constant. P2 Norms coded (-1=Extremely socially inappropriate; -0.6=Very socially inappropriate; -0.2=Somewhat socially inappropriate; +0.2=Somewhat socially appropriate; +0.6=Very socially appropriate; +1=Extremely socially appropriate). P3 Norms coded (-1=None of my peers; -0.6=Only a few of my peers; -0.2=Some of my peers; +0.2=A lot of my peers; +0.6=Most of my peers; +1=All of my peers).

^b^Carbon monoxide readings not available for one Northern Irish school and two Colombian schools (excluded from analysis).

*******p<0.01; **p<0.05; *p<0.10.**Results of mixed-effects ordered logistic regressions showing relationship between anti-smoking behavior and responses to survey smoking norm questions**

|  | **Dependent variable: Anti-smoking behavior** | | | | | | | | | | | |
| --- | --- | --- | --- | --- | --- | --- | --- | --- | --- | --- | --- | --- |
|  | **IN1** | **IN2** | **IN3^b^** | **IN4** | **IN5** | **IN6^b^** | **IN7** | **DN1** | **DN2** | **DN3** | **DN4** | **DN5** |
|  | *OR*  (95% CI) | *OR*  (95% CI) | *OR*  (95% CI) | *OR*  (95% CI) | *OR*  (95% CI) | *OR*  (95% CI) | *OR*  (95% CI) | *OR*  (95% CI) | *OR*  (95% CI) | *OR*  (95% CI) | *OR*  (95% CI) | *OR*  (95% CI) |
| **n** | 1566 | 1567 | 1564 | 1563 | 1565 | 1566 | 1566 | 1567 | 1568 | 1567 | 1567 | 1567 |
| **Norm^a^** | 1.60***  (1.36, 1.89) | 1.86***  (1.42, 2.43) | 1.37***  (1.15, 1.64) | 1.14  (0.98, 1.32) | 1.14*  (0.99, 1.32) | 1.58***  (1.38, 1.81) | 1.64***  (1.42, 1.90) | 2.49***  (2.15, 2.88) | 1.41***  (1.27, 1.56) | 1.36***  (1.24, 1.49) | 1.53***  (1.35, 1.74) | 1.33***  (1.14, 1.55) |
| **Sex** |  |  |  |  |  |  |  |  |  |  |  |  |
| *Girl/PNTS* | 1.11  (0.84, 1.47) | 1.11  (0.84, 1.48) | 1.16  (0.88, 1.54) | 1.12  (0.85, 1.48) | 1.13  (0.85, 1.49) | 1.07  (0.80, 1.42) | 1.03  (0.78, 1.38) | 1.10  (0.82, 1.47) | 1.15  (0.87, 1.52) | 1.12  (0.84, 1.48) | 1.16  (0.87, 1.54) | 1.11  (0.84, 1.48) |
| **Intervention** |  |  |  |  |  |  |  |  |  |  |  |  |
| *Dead Cool* | 1.42  (0.86, 2.35) | 1.36  (0.81, 2.30) | 1.32  (0.78, 2.21) | 1.37  (0.82, 2.30) | 1.36  (0.81, 2.27) | 1.40  (0.88, 2.23) | 1.39  (0.85, 2.28) | 1.28  (0.76, 2.15) | 1.32  (0.81, 2.14) | 1.32  (0.81, 2.15) | 1.34  (0.83, 2.19) | 1.39  (0.84, 2.29) |
| **Country** |  |  |  |  |  |  |  |  |  |  |  |  |
| *Colombia* | 0.51***  (0.31, 0.85) | 0.54**  (0.32, 0.91) | 0.56**  (0.33, 0.93) | 0.53**  (0.31, 0.88) | 0.53**  (0.32, 0.89) | 0.59**  (0.37, 0.93) | 0.61**  (0.37, 0.99) | 0.50***  (0.30, 0.84) | 0.45***  (0.28, 0.74) | 0.49***  (0.30, 0.80) | 0.53***  (0.32, 0.86) | 0.53**  (0.32, 0.88) |

^a^Note on interpretation: these are proportional odds ratios representing the multiplicative change in odds of **at least** a one-unit increase in response category to the smoking behavior question (more anti-smoking) for a one-unit increase in response category to the relevant norm question (increasing perceptions that important others think you should not smoke/increasing perceptions that important others do not smoke often), holding other variables constant.

^b^Approximate likelihood-ratio test indicates potential violation of the proportional odds assumption (p<0.05).

*******p<0.01; **p<0.05; *p<0.10.

**Results of mixed-effects ordered logistic regressions showing relationship between anti-smoking intentions and responses to survey smoking norm questions.**

|  | **Dependent variable: Anti-smoking intentions** | | | | | | | | | | | |
| --- | --- | --- | --- | --- | --- | --- | --- | --- | --- | --- | --- | --- |
|  | **IN1^b^** | **IN2** | **IN3** | **IN4** | **IN5^b^** | **IN6** | **IN7^b^** | **DN1** | **DN2** | **DN3** | **DN4** | **DN5** |
|  | *OR*  (95% CI) | *OR*  (95% CI) | *OR*  (95% CI) | *OR*  (95% CI) | *OR*  (95% CI) | *OR*  (95% CI) | *OR*  (95% CI) | *OR*  (95% CI) | *OR*  (95% CI) | *OR*  (95% CI) | *OR*  (95% CI) | *OR*  (95% CI) |
| **n** | 1563 | 1564 | 1561 | 1560 | 1562 | 1563 | 1563 | 1564 | 1565 | 1564 | 1564 | 1564 |
| **Norm^a^** | 1.81***  (1.56, 2.11) | 1.86***  (1.44, 2.41) | 1.37***  (1.17, 1.62) | 1.25***  (1.09, 1.44) | 1.25***  (1.10, 1.43) | 1.75***  (1.54, 1.98) | 1.79***  (1.56, 2.04) | 1.85***  (1.61, 2.13) | 1.31***  (1.18, 1.45) | 1.25***  (1.15, 1.37) | 1.41***  (1.25, 1.60) | 1.29***  (1.11, 1.50) |
| **Sex** |  |  |  |  |  |  |  |  |  |  |  |  |
| *Girl/PNTS* | 0.99  (0.76, 1.28) | 0.99  (0.77, 1.29) | 1.03  (0.79, 1.33) | 0.99  (0.76, 1.28) | 0.999  (0.77, 1.30) | 0.93  (0.72, 1.21) | 0.90  (0.69, 1.18) | 0.98  (0.75, 1.27) | 1.03  (0.79, 1.33) | 0.99  (0.77, 1.29) | 1.03  (0.79, 1.34) | 0.999  (0.77, 1.30) |
| **Intervention** |  |  |  |  |  |  |  |  |  |  |  |  |
| *Dead Cool* | 1.20  (0.81, 1.76) | 1.14  (0.76, 1.71) | 1.12  (0.74, 1.71) | 1.16  (0.77, 1.74) | 1.15  (0.76, 1.72) | 1.21  (0.85, 1.73) | 1.19  (0.82, 1.73) | 1.11  (0.77, 1.59) | 1.13  (0.76, 1.67) | 1.14  (0.78, 1.68) | 1.15  (0.78, 1.71) | 1.16  (0.78, 1.72) |
| **Country** |  |  |  |  |  |  |  |  |  |  |  |  |
| *Colombia* | 0.49***  (0.33, 0.73) | 0.53***  (0.35, 0.80) | 0.53***  (0.35, 0.81) | 0.50***  (0.33, 0.75) | 0.51***  (0.34, 0.76) | 0.56***  (0.39, 0.80) | 0.59***  (0.40, 0.86) | 0.50***  (0.35, 0.72) | 0.46***  (0.31, 0.68) | 0.49***  (0.33, 0.72) | 0.51***  (0.35, 0.76) | 0.51***  (0.34, 0.77) |

^a^Note on interpretation: these are proportional odds ratios representing the multiplicative change in odds of **at least** a one-unit increase in response category to the smoking intentions question (greater intentions not to smoke) for a one-unit increase in response category to the relevant norm question (increasing perceptions that important others think you should not smoke/increasing perceptions that important others do not smoke often), holding other variables constant.

^b^Approximate likelihood-ratio test indicates potential violation of the proportional odds assumption (p<0.05).

*******p<0.01; **p<0.05; *p<0.10.

**Results of mixed-effects linear regressions showing relationship between objectively measured smoking behavior and responses to survey smoking norm questions**

|  | **Dependent variable: Objective smoking behavior (expelled air carbon monoxide, ppm)^b^** | | | | | | | | | | | |
| --- | --- | --- | --- | --- | --- | --- | --- | --- | --- | --- | --- | --- |
|  | **IN1** | **IN2** | **IN3** | **IN4** | **IN5** | **IN6** | **IN7** | **DN1** | **DN2** | **DN3** | **DN4** | **DN5** |
|  | *b*  (95% CI) | *b*  (95% CI) | *b*  (95% CI) | *b*  (95% CI) | *b*  (95% CI) | *b*  (95% CI) | *b*  (95% CI) | *b*  (95% CI) | *b*  (95% CI) | *b*  (95% CI) | *b*  (95% CI) | *b*  (95% CI) |
| **n** | 1202 | 1203 | 1200 | 1199 | 1201 | 1202 | 1202 | 1203 | 1204 | 1203 | 1203 | 1203 |
| **Norm^a^** | -0.09  (-0.26, 0.08) | -0.07  (-0.29, 0.14) | 0.05  (-0.03, 0.12) | -0.01  (-0.10, 0.07) | -0.04  (-0.13, 0.06) | -0.004  (-0.09, 0.08) | -0.08**  (-0.15, -0.005) | -0.05  (-0.17, 0.07) | -0.04  (-0.09, 0.02) | -0.01  (-0.06, 0.05) | -0.05  (-0.24, 0.13) | 0.0003  (-0.09, 0.09) |
| **Sex** |  |  |  |  |  |  |  |  |  |  |  |  |
| *Girl/PNTS* | -0.0001  (-0.15, 0.15) | -0.001  (-0.14, 0.14) | 0.01  (-0.13, 0.15) | 0.001  (-0.14, 0.15) | 0.002  (-0.14, 0.14) | 0.001  (-0.14, 0.15) | 0.01  (-0.14, 0.16) | -0.01  (-0.15, 0.14) | -0.01  (-0.15, 0.13) | -0.004  (-0.14, 0.14) | -0.01  (-0.15, 0.14) | -0.002  (-0.14, 0.14) |
| **Intervention** |  |  |  |  |  |  |  |  |  |  |  |  |
| *Dead Cool* | -0.49**  (-0.95, -0.04) | -0.49**  (-0.94, -0.04) | -0.50**  (-0.95, -0.05) | -0.49**  (-0.94, -0.04) | -0.49**  (-0.94, -0.04) | -0.50**  (-0.95, -0.05) | -0.49**  (-0.95, -0.04) | -0.49**  (-0.94, -0.03) | -0.48**  (-0.94, -0.03) | -0.50**  (-0.95, -0.05) | -0.49**  (-0.95, -0.03) | -0.50**  (-0.95, -0.04) |
| **Country** |  |  |  |  |  |  |  |  |  |  |  |  |
| *Colombia* | 1.81***  (1.36, 2.26) | 1.80***  (1.35, 2.25) | 1.82***  (1.37, 2.28) | 1.81***  (1.36, 2.26) | 1.80***  (1.36, 2.25) | 1.81***  (1.36, 2.25) | 1.79***  (1.35, 2.23) | 1.81***  (1.36, 2.26) | 1.82***  (1.37, 2.27) | 1.81***  (1.35, 2.27) | 1.81***  (1.35, 2.26) | 1.81***  (1.36, 2.26) |
| **Constant** | 1.88***  (1.54, 2.22) | 1.87***  (1.41, 2.34) | 1.63***  (1.33, 1.93) | 1.74***  (1.50, 1.98) | 1.77***  (1.52, 2.03) | 1.73***  (1.48, 1.99) | 1.85***  (1.60, 2.10) | 1.98***  (1.40, 2.57) | 1.87***  (1.50, 2.25) | 1.75***  (1.52, 1.99) | 1.98***  (1.10, 2.87) | 1.73***  (1.26, 2.19) |

^a^Note on interpretation: these are regression coefficients representing the increase in carbon monoxide reading (more smoking) for a one-unit increase in response category to the relevant norm question (increasing perceptions that important others think you should not smoke/increasing perceptions that important others do not smoke often), holding other variables constant.

^b^Carbon monoxide readings not available for one Northern Irish school and two Colombian schools (excluded from analysis).

*******p<0.01; **p<0.05; *p<0.10.

**Supplement 6: Model fit statistics and diagrams showing structure and standardized factor loadings from confirmatory factor analyses**

**Model fit statistics**

| **MODEL** | **Obs^a^** | **Χ^2^** | **df** | **CFI** | **TLI** | **RMSEA (90% CI)** | **SRMR** | **AIC** | **BIC** | **BIC (adjusted)** |
| --- | --- | --- | --- | --- | --- | --- | --- | --- | --- | --- |
| **1** | 1576 | 56.421**** | 17 | 0.977 | 0.962 | 0.047 (0.034, 0.061) | 0.026 | 8106.089 | 8250.880 | 8165.107 |
| **2** | 1567 | 61.719**** | 11 | 0.972 | 0.947 | 0.059 (0.045, 0.074) | 0.029 | 22216.848 | 22345.415 | 22269.172 |
| **3** | 1575 | - | - | 1.000 | 1.000 | 0.000 (0.000, 0.000) | 0.000 | 3478.691 | 3505.501 | 3489.617 |
| **4** | 1568 | 1.496 | 3 | 1.000 | 1.017 | 0.000 (0.000, 0.043) | 0.007 | 20434.250 | 20525.328 | 20471.323 |
| **5** | 1635 | 434.569**** | 196 | 0.959 | 0.952 | 0.030 (0.026, 0.034) | 0.033 | 54004.817 | 54431.369 | 54180.399 |
| **6** | 1635 | 440.713**** | 198 | 0.958 | 0.951 | 0.030 (0.026, 0.034) | 0.034 | 54010.895 | 54426.649 | 54182.033 |

^a^Missing data are imputed using full information maximum likelihood (FIML). Cases are included as long as the pupil completed at least one of the relevant items on the experiment or survey.

Χ^2^ = Chi-square Goodness of Fit statistic; df = degrees of freedom; CFI = Comparative Fit Index; TLI = Tucker Lewis Index; RMSEA = Root Mean Square Error of Approximation; CI = confidence interval; SRMR = Standardized Root Mean Square Residual; AIC = Akaike Information Criterion; BIC = Bayesian Information Criterion; *p<0.10; **p<0.05; ***p<0.01; ****p≤0.001.

**Model 1: Measurement model for experimental measure of injunctive norms, standardized factor loadings, *p<0.10; **p<0.05; ***p<0.01; ****p≤0.001**

P2S2

0.02****

0.09****

P2S3

0.02****

P2S9

P2S8

P2S7

P2S6

P2S5

P2S4

0.16****

0.16****

0.22****

0.26****

0.30****

0.28****

0.02****

0.18****

**Model 2: Measurement model for survey measure of injunctive norms, standardized factor loadings, *p<0.10; **p<0.05; ***p<0.01; ****p≤0.001**

IN1

0.04****

0.39****

IN2

IN7

0.22****

IN3

0.24****

0.33****

0.43****

0.13****

IN4

0.40****

IN5

0.52****

IN6

0.47****

**Model 3: Measurement model for experimental measure of descriptive norms, standardized factor loadings, *p<0.10; **p<0.05; ***p<0.01; ****p≤0.001**

P3Q1

P3Q2

0.44****

0.44****

**Model 4: Measurement model for survey measure of descriptive norms, standardized factor loadings, *p<0.10; **p<0.05; ***p<0.01; ****p≤0.001**

DN1

0.31****

DN2

0.36****

0.52****

0.37****

DN3

0.37****

DN4

0.32****

DN5

0.01

**Model 5: First-order measurement model with four correlated latent variables, standardized factor loadings,**

P2S2

***p<0.10; **p<0.05; ***p<0.01; ****p≤0.001**

0.02****

0.08****

P2S3

0.02****

0.16****

P2S4

0.16****

P2S5

0.22****

0.26****

P2S6

0.30****

P2S7

0.28****

0.02****

P2S8

-0.24****

0.18****

P2S9

IN1

0.39****

IN2

0.33****

0.21****

0.04****

IN3

0.30****

-0.18****

0.42****

IN4

0.14****

0.38****

IN5

0.55****

-0.21****

IN6

0.49****

0.21****

IN7

0.44****

P3Q1

0.28****

0.44****

-0.23****

0.05

0.35****

0.37****

0.27****

0.31****

0.39****

0.52****

DN5

DN4

DN3

DN2

DN1

P3Q2

**Model 6: Second-order measurement model with four first-order latent variables, standardized factor loadings,**

P2S2

***p<0.10; **p<0.05; ***p<0.01; ****p≤0.001**

0.02****

0.07****

P2S3

0.02****

0.13****

P2S4

0.13****

P2S5

0.18****

0.21****

P2S6

0.25****

P2S7

0.23****

0.02****

P2S8

0.15****

-0.69****

P2S9

IN1

0.35****

IN2

0.19****

0.04****

IN3

0.28****

0.47****

0.39****

IN4

0.34****

0.14****

IN5

0.50****

-0.67****

IN6

0.44****

0.22****

0.45****

IN7

0.37****

P3Q1

0.37****

DN5

DN4

DN3

DN2

DN1

P3Q2

0.25****

0.30****

0.36****

0.48****

0.32****

0.04

0.34****

**Supplement 7: Statistical information and decisions on whether individual items are demonstrating differential item functioning**

| **Parameter** | **MI** | **EPC** | **NCP** | **Power** | **Decision** |
| --- | --- | --- | --- | --- | --- |
| ***P2S2 on Country*** | 62.674 | -0.101 | 61.139 | 1.000 | DIF |
| ***P2S3 on Country*** | 0.211 | -0.007 | 39.587 | 1.000 | No DIF |
| ***P2S4 on Country*** | 8.375 | 0.044 | 43.540 | 1.000 | No DIF |
| ***P2S5 on Country*** | 49.588 | -0.141 | 24.945 | 0.999 | DIF |
| ***P2S6 on Country*** | 15.599 | 0.072 | 29.959 | 1.000 | No DIF |
| ***P2S7 on Country*** | 7.287 | -0.046 | 35.144 | 1.000 | No DIF |
| ***P2S8 on Country*** | 39.421 | 0.103 | 36.922 | 1.000 | DIF |
| ***P2S9 on Country*** | 0.556 | -0.012 | 41.864 | 1.000 | No DIF |
| ***IN1 on Country*** | 14.413 | 0.123 | 9.562 | 0.871 | DIF |
| ***IN2 on Country*** | 1.969 | 0.024 | 33.218 | 1.000 | No DIF |
| ***IN3 on Country*** | 14.034 | -0.119 | 9.947 | 0.884 | DIF |
| ***IN4 on Country*** | 7.568 | 0.114 | 5.800 | 0.673 | DIF |
| ***IN5 on Country*** | 0.081 | 0.012 | 5.299 | 0.634 | Inconclusive |
| ***IN6 on Country*** | 0.000 | -0.001 | 7.390 | 0.776 | Inconclusive |
| ***IN7 on Country*** | 13.719 | -0.122 | 9.188 | 0.858 | DIF |
| ***P3Q1 on Country*** | 13.100 | 0.068 | 28.050 | 1.000 | No DIF |
| ***P3Q2 on Country*** | 13.100 | -0.068 | 28.050 | 1.000 | No DIF |
| ***DN1 on Country*** | 3.854 | -0.078 | 6.362 | 0.713 | Inconclusive |
| ***DN2 on Country*** | 25.867 | 0.305 | 2.787 | 0.386 | DIF |
| ***DN3 on Country*** | 0.676 | 0.052 | 2.469 | 0.349 | Inconclusive |
| ***DN4 on Country*** | 6.085 | -0.109 | 5.090 | 0.616 | DIF |
| ***DN5 on Country*** | 7.700 | -0.103 | 7.256 | 0.768 | DIF |

MI: modification indices; EPC: expected parameter change; NCP: non-centrality parameter; DIF: differential item functioning.

**Supplement 8: Effects of sex and psycho-social variables on first-order and second-order norms latent variables, standardized regression coefficients**

| **Predictor** | **Β (SE)** | **p-value** |
| --- | --- | --- |
| **Experiment Injunctive Norms (first-order latent)** | | |
| Sex^a^ | -0.14 (0.07) | 0.046 |
| Need to Belong Scale^b^ | -0.29 (0.07) | <0.001 |
| Fear of Negative Evaluation^c^ | -0.10 (0.07) | 0.16 |
| Pro-social Behavior^d^ | -0.10 (0.02) | <0.001 |
| Big 5 (Openness)^e^ | -0.18 (0.06) | 0.001 |
| Big 5 (Extraversion)^e^ | -0.10 (0.05) | 0.06 |
| Big 5 (Agreeableness)^e^ | -0.48 (0.06) | <0.001 |
| Big 5 (Conscientiousness)^e^ | -0.36 (0.06) | <0.001 |
| Big 5 (Stability)^e^ | -0.19 (0.05) | <0.001 |
| Rule-following^f^ | -0.004 (0.002) | 0.05 |
| **Survey Injunctive Norms (first-order latent)** | | |
| Sex^a^ | 0.07 (0.07) | 0.30 |
| Need to Belong Scale^b^ | 0.04 (0.07) | 0.61 |
| Fear of Negative Evaluation^c^ | -0.04 (0.06) | 0.50 |
| Pro-social Behavior^d^ | 0.12 (0.02) | <0.001 |
| Big 5 (Openness)^e^ | 0.19 (0.05) | <0.001 |
| Big 5 (Extraversion)^e^ | 0.15 (0.05) | 0.002 |
| Big 5 (Agreeableness)^e^ | 0.40 (0.05) | <0.001 |
| Big 5 (Conscientiousness)^e^ | 0.36 (0.05) | <0.001 |
| Big 5 (Stability)^e^ | 0.17 (0.04) | <0.001 |
| Rule-following^f^ | 0.002 (0.002) | 0.38 |
| **Experiment Descriptive Norms (first-order latent)** | | |
| Sex^a^ | 0.08 (0.07) | 0.24 |
| Need to Belong Scale^b^ | -0.15 (0.07) | 0.02 |
| Fear of Negative Evaluation^c^ | 0.05 (0.06) | 0.42 |
| Pro-social Behavior^d^ | -0.07 (0.02) | <0.001 |
| Big 5 (Openness)^e^ | -0.06 (0.05) | 0.29 |
| Big 5 (Extraversion)^e^ | -0.01 (0.05) | 0.77 |
| Big 5 (Agreeableness)^e^ | -0.32 (0.06) | <0.001 |
| Big 5 (Conscientiousness)^e^ | -0.33 (0.06) | <0.001 |
| Big 5 (Stability)^e^ | -0.22 (0.05) | <0.001 |
| Rule-following^f^ | -0.001 (0.002) | 0.71 |
| **Survey Descriptive Norms (first-order latent)** | | |
| Sex^a^ | -0.03 (0.08) | 0.74 |
| Need to Belong Scale^b^ | 0.02 (0.08) | 0.79 |
| Fear of Negative Evaluation^c^ | -0.10 (0.08) | 0.19 |
| Pro-social Behavior^d^ | 0.07 (0.03) | 0.005 |
| Big 5 (Openness)^e^ | 0.18 (0.07) | 0.01 |
| Big 5 (Extraversion)^e^ | 0.03 (0.06) | 0.58 |
| Big 5 (Agreeableness)^e^ | 0.33 (0.06) | <0.001 |
| Big 5 (Conscientiousness)^e^ | 0.33 (0.07) | <0.001 |
| Big 5 (Stability)^e^ | 0.32 (0.07) | <0.001 |
| Rule-following^f^ | 0.005 (0.003) | 0.04 |
| **Anti-Smoking/Vaping Norms (second-order latent)** | | |
| Sex^a^ | 0.05 (0.08) | 0.52 |
| Need to Belong Scale^b^ | 0.25 (0.08) | 0.003 |
| Fear of Negative Evaluation^c^ | -0.02 (0.08) | 0.76 |
| Pro-social Behavior^d^ | 0.16 (0.02) | <0.001 |
| Big 5 (Openness)^e^ | 0.25 (0.07) | <0.001 |
| Big 5 (Extraversion)^e^ | 0.12 (0.05) | 0.03 |
| Big 5 (Agreeableness)^e^ | 0.71 (0.08) | <0.001 |
| Big 5 (Conscientiousness)^e^ | 0.62 (0.07) | <0.001 |
| Big 5 (Stability)^e^ | 0.37 (0.06) | <0.001 |
| Rule-following^f^ | 0.004 (0.002) | 0.06 |

^a^In all analyses, sex is coded (0=Boy; 1=Girl/Prefer not to say).

^b^Average of 10 items, coded 1-5. Not available for two Colombian schools (excluded from analysis).

^c^Average of 12 items, coded 1-5. Not available for two Colombian schools (excluded from analysis).

^d^Sum of five items, coded 0-2.

^e^Average of 10 items, coded 0-4.

^f^Number of balls allocated to the blue bucket in Part 1 of the experiments.

1. Only one participant failed to allocate all 50 balls during the baseline experiment. There were several changes made to the experimental protocol after baseline was completed in the first pilot school in Northern Ireland. The first version of the experiment included a forced waiting time for the RF task of seven minutes, the idea being to make sure that all pupils proceeded to the second part together. Subsequently it was decided to reduce the time allocated for the RF task from seven minutes to five minutes, to remove the forced waiting time and to insert dummy screens informing participants when to wait for further instructions from the experimenter. [↑](#footnote-ref-1)
2. The measurement property of content validity suggests that in order to be considered adequate, a measurement instrument should adequately reflect the underlying theoretical construct being measured^13^. Thus, one of the advantages of the Krupka-Weber method of eliciting social norms is that the structure of the game itself provides incentives for people to report their beliefs about others’ beliefs about social appropriateness. The existence of such shared “second-order” beliefs are a theoretical precondition for the existence of a social norm according to the work of Bicchieri^14^. [↑](#footnote-ref-2)
